# The Selection of the Best Derivatization Reagents for the Determination of Polyamines in Home-Made Wine Samples

**DOI:** 10.3390/ma16041474

**Published:** 2023-02-09

**Authors:** Anna Kmieciak, Aneta Jastrzębska, Karolina Szymańska, Marek P. Krzemiński, Tadeusz M. Muzioł, Marzanna Kurzawa, Edward Szłyk

**Affiliations:** 1 Department of Organic Chemistry, Faculty of Chemistry, Nicolaus Copernicus University in Toruń, Gagarin 7 Str., 87-100 Toruń, Poland; 2 Department of Analytical Chemistry and Applied Spectroscopy, Faculty of Chemistry, Nicolaus Copernicus University in Toruń, Gagarin 7 Str., 87-100 Toruń, Poland; 3 Department of Inorganic and Coordination Chemistry, Faculty of Chemistry, Nicolaus Copernicus University in Toruń, Gagarin 7 Str., 87-100 Toruń, Poland

**Keywords:** polyamines, 2-chloro-1,3-dinitro-5-(trifluoromethyl)benzene, 1-fluoro-2-nitro-4-(trifluoromethyl)benzene, 3,5-bis-(trifluoromethyl)phenyl isothiocyanate, NMR, XRD, HPLC

## Abstract

The procedures of putrescine, spermine, spermidine, and cadaverine derivatization using 2-chloro-1,3-dinitro-5-(trifluoromethyl)benzene, 1-fluoro-2-nitro-4-(trifluoromethyl) benzene, and 3,5-bis-(trifluoromethyl)phenyl isothiocyanate for chromatographic determination in home-made wine samples are compared in the present study. The procedures discussed were compared regarding simplicity, linearity, precision, and accuracy. The polyamines derivatives were isolated and characterized by X-ray crystallography and ^1^H, ^13^C, and ^19^F NMR spectroscopy. The obtained structures of aliphatic amines showed that all amino groups, four in spermine, two in putrescine and cadaverine, and three in spermidine, regardless of the applied reagent, were substituted. The applicability of the described procedures was tested during the chromatographic analysis of the compounds’ content in home-made wines. For this purpose, a simple and environmentally friendly sample preparation procedure was developed. The obtained results present the derivatization of polyamines with 1-fluoro-2-nitro-4-(trifluoromethyl)benzene as a better choice for the determination of these compounds in food samples.

## 1. Introduction

Polyamines (PAs), such as spermine (Spm), spermidine (Spd), and putrescine (Put), are polycationic biogenic amines required for both eukaryotic cell growth and differentiation. PAs are involved in various biological processes, notably cell proliferation and differentiation, and also have antioxidant properties [1,2]. It is well known that in the human organism, polyamine levels are mainly regulated by de novo synthesis. However, polyamine biosynthetic ability decreases with age, and, at present, it is a process that is difficult to control. On the other hand, polyamines in the organism can also be formed due to exogenous processes mainly caused by protein-rich food degradation. Polyamines can be found in all types of foods in a wide range of concentrations. Spd and Spm are naturally present in food, whereas Put could also have an amicrobial origin. The main polyamine in plant-based products is Spd, whereas Spm content is generally higher in animal-derived foods. However, their elevated concentrations indicate food spoilage, mainly due to microbial enzymatic activity [3]. Cadaverine (Cad) is a less common polyamine detected in protein-rich foods. Dietary polyamines are absorbed in the small intestine through transcellular or paracellular pathways and are distributed to the different tissues. Therefore, in order to control the polyamine concentration in the body, it is necessary to bear in mind the amount of these compounds ingested from food, since they can positively or negatively affect human health [4,5]. Special attention should be paid to wines because a certain number of polyamines are present in grapes, such as putrescine, spermidine, and spermine. They can also be formed by microorganisms during the winemaking process, e.g., cadaverine [6]. Spermine and spermidine, which are present in grapes, are degraded during alcoholic fermentation. In contrast, the concentration of putrescine can increase considerably during this process.

Among the discussed amines, Put and Cad are considered toxic for humans [7,8]. These compounds can lead to health problems, such as abnormal blood pressure, human allergic reactions, headaches, and tachycardia/worsening asthma. Moreover, they can improve the toxic reaction of histamine (Him) and tyramine (Tym) and interact with nitrites to generate nitrosamines. On the contrary, spermine and spermidine have no direct adverse health effects, but they can react with nitrite ions forming carcinogenic nitrosamines, along with the toxicity increase in other BAs [8]. Considering the influence of these compounds on human health, and the fact that ca. 600 million people yearly have health problems due to eating unsafe food [9], it seems reasonable to develop a rapid and straightforward method for their determination.

The number of publications on biogenic amines (including PAs) in food has systematically increases in recent decades. Many conventional, modern, and innovative methods for these compounds’ determination have been proposed and reviewed [7,10,11,12,13]. Nevertheless, most analytical methodologies for polyamines’ determination in food samples are complicated because the levels of these compounds are low. Moreover, a pre- or post-column derivatization process is applied due to the insufficient absorption properties of PAs in visible and ultraviolet wavelength ranges. The structures of BAs give rise to a crucial question regarding the reactivity of primary and secondary amino groups in spermidine and spermine. Putrescine and cadaverine have terminal α,ω-primary amino groups, while spermidine and spermine also have one and two secondary amino groups in their structure, respectively. To the best of our knowledge, the reactions of spermidine and spermine with derivatization reagents using nucleophilic aromatic substitution reactions and proving the structures of the obtained amine derivatives have not been presented and discussed.

In our previous papers, we described 2-chloro-1,3-dinitro-5-(trifluoromethyl)benzene (CNBF) [14], 1-fluoro-2-nitro-4-(trifluoromethyl)benzene (FNBT) [15], and 3,5-bis-(trifluoromethyl)phenyl isothiocyanate (BPI) [16] reagents for the chromatographic determination of monoamines: tyramine (Tym), phenylethylamine (Phe), histamine (Him), and tryptamine (Try) BAs in beverages samples. The developed syntheses resulted in the preparation of pure derivatives of Him, Tym, Phe, and Try without by-products. Despite the simple and effective synthesis conditions previously described by us, di-, tri, and tetraamines, such as putrescine (Put), cadaverine (Cad), spermidine (Spd), and spermine (Spm), were not tested.

Therefore, in this work, modified procedures for the chromatographic determination of polyamines Put, Cad, Spm, and Spd, using a derivatization reaction with CNBF, FNBT, and BPI, are studied. The scope of the research focuses on CNBF, FNBT, and BPI derivatization reaction conditions in order to obtain the best yield of polyamine derivatives. Obtained PA derivatives are tested for their purity, and their structures are confirmed by ^1^H, ^13^C, ^19^F NMR, and IR spectroscopy, and single-crystal X-ray crystallography. The linearity, repeatability, accuracy, detection, and quantification limits for the proposed analytical procedures are calculated and discussed. Moreover, the proposed reagents and derivatization strategies are compared concerning simplicity, reactivity, and application in food-sample analysis.

The developed procedure is applied for the determination of the discussed amines in home-made wines (four red, two white, chokeberry, elderberry, and apple wines) using the HPLC technique. Based on the literature, there are few publications [17,18] on the subject of biogenic amines’ content in home-made wines, especially in alcoholic beverages obtained from fermented fruits, such as chokeberry, elderberry, and apple. Additionally, in order to determine the quality of the studied wines, the content of the selected biogenic monoamines (Him, Tym, Try, and Phe) is discussed.

## 2. Experimental

### 2.1. Reagents and Apparatus

Analytical grade cadaverine (Cad), putrescine (Put), spermine (Spm), spermidine (Spd), tyramine (Tym), histamine (Him), tryptamine (Try), 2-phenylethylamine, 99% (Phe), 2-chloro-1,3-dinitro-5-(trifluoromethyl)benzene (CNBF), 1-fluoro-2-nitro-4-(trifluoromethyl)benzene (FNBT), 3,5-bis(trifluoromethyl)phenyl isothiocyanate (BPI), acetonitrile (HPLC grade), *N,N*-diisopropylethylamine (DIPEA)**,** acetone-*d6*, chloroform-*d* (CDCl_3_), tetrahydrofuran-*d8* (THF-*d8*), dimethyl sulfoxide-*d6* (DMSO-*d6*), hide powder, and polyvinyl pyrolidone (PVP 40) were purchased from Sigma Aldrich (Poland). Tetrahydrofuran, THF (for HPLC), acetone, ethyl acetate, ethanol (EtOH), methanol (MeOH), dichloromethane (DCM), and hexane were purchased from Alchem (Poland). Chitosan 85/1000/A1 was purchased from BioLog Heppe GmbH Gewerbegebiet Queis Max-Planck-Ring, whereas kappa-carrageenan, type I and pectin, amidated, low ester were obtained from Pol-Aura (Poland).

The HPLC system (Liquid Chromatograph LC-20AD, Shimadzu Corporation, Kyoto, Japan), equipped with an autosampler SIL- 20AC HT and a photodiode multi-wavelength detector (SPD-M20A Prominence Diode Array Detector, Shimadzu Corporation, Kyoto, Japan) was applied. Analyses were performed on a Gemini 5 um NX-C18 110A, LC column (250 × 4.6 mm) at 35 °C. The samples were purified by flash chromatography (Biotage Select Systems, Shimadzu Corporation, Kyoto, Japan). NMR spectra were recorded on a Bruker Avance III 400 MHz spectrometer (Bruker, Ettlingen, Germany) at 400 MHz for ^1^H, 100 MHz for ^13^C, and 375 MHz for ^19^F frequency resonances at 298 ± 1 K. ^1^H NMR spectra of Put-FNBT and Spm-BPI were recorded on a Bruker Avance III 700 MHz spectrometer (Bruker, Ettlingen, Germany) using CDCl_3_, DMSO-*d6*, acetone-*d6*, and acetonitrile-*d3* as solvents. Chemical shifts were reported using the residual solvent peaks as references: CDCl_3_ (δ 7.26 ppm), DMSO-*d6* (δ 2.50 ppm), acetone-*d6* (δ 2.05 ppm), acetonitrile-*d3* (δ 1.94 ppm) for ^1^H NMR and relative to the central CDCl_3_ (δ 77.00 ppm), DMSO-*d6* (δ 39.51 ppm), acetone-*d6* (δ 29.92 ppm), and acetonitrile-*d3* (δ 1.39 ppm) resonances for ^13^C NMR. Spin–spin coupling constants (*J*) are presented in [Hz]. Infrared spectra were recorded on a Bruker Alpha Platinum-ATR spectrometer (Bruker, Ettlingen, Germany) with OPUS 7.5 software using the ATR technique. Melting points were obtained in open-capillary tubes using electrothermal digital melting-point apparatus from Cole-Parmer Ltd. (Saint Neots, United Kingdom), and were uncorrected. The diffraction data of the studied compounds were collected for the single crystal on a Rigaku XtaLAB Synergy (Dualflex) diffractometer with a HyPix detector with a monochromated CuKα X-ray source (λ = 1.54184 Å) and BESSY II synchrotron, (Helmholtz Zentrum, Berlin, Germany). Statistica Ultimate 13 (StatSoft Polska, Kraków, Poland) was applied for the statistical evaluation of the obtained results.

### 2.2. Selection of Molar Ratio for Polyamines’ Derivatization

The impact of the reagents ratio on the derivatives’ yield was examined in order to obtain optimal derivatization reaction conditions. The reactions of spermidine and spermine with two equivalents of CNBF, FNBT, and BPI resulted in the mixtures of products. Moreover, in addition to compounds substituted in the terminal amino groups, fully substituted derivatives were also formed in secondary amino groups. Therefore, an excess of reagents was used that was adjusted to the number of amino groups (e.g., for Spd 1:3.3 equiv. of CNBF, FNBT, BPI). Obtained derivatives were purified, dissolved in an appropriate solvent or solution, and identified by NMR and HPLC techniques, which confirmed their purity and maximal yield. The optimal conditions of the derivatives’ synthesis were applied for further research.

### 2.3. Derivatization Process—General Procedures

Diamines (Put and Cad) reacted with the derivatizing reagent at a 1/2.2 molar ratio, whereas spermidine (Spd, triamine) and spermine (Spm, tetraamine) was at 1/3.3 and 1/4.4 ratios, respectively. The syntheses of the appropriate derivatives for individual amines were conducted analogously. Representative procedures with details for the preparation of putrescine derivatives from CNBF, FNBT, and BPI were as follows: CNBF (0.5952 g, 2.2 mmol) was mixed with Put (0.0882 g, 1 mmol) and DIPEA (0.3102 g, 0.418 mL, 2.4 mmol) in ethanol (5.0 mL). The reaction mixture was refluxed for 1.5 h, followed by solvent removal under reduced pressure. The obtained crude product was purified by column chromatography on a silica gel with hexane/ethyl acetate (80/20) as an eluent producing a final yellow solid (0.5230 g, 94% yield), which was recrystallized from methanol resulting in yellow crystals (MP 190–194 °C).

FNBT (0.4600 g, 0.308 mL, 2.2 mmol) was added to the solution of Put (0.0882 g, 0.101 mL, 1 mmol) and DIPEA (0.3102 g, 0.418 mL, 2.4 mmol) in EtOH (5.0 mL). The reaction mixture was stirred for 1.5 h and evaporated under vacuum to yield a crude product. Purification on a silica gel column with hexane/acetone (80:20) as the eluent produced a yellow solid (0.4290 g, 92% yield, MP 138–140 °C).

BPI (0.1491 g, 100.4 µL, 0.55 mmol) was added to the solution of Put (0.0220 g, 25.1 µL, 0.25 mmol) in THF (5.0 mL) and the reaction mixture was stirred for 3 h, followed by solvent evaporation under reduced pressure. The crude product was purified on a column with silica gel, using DCM/MeOH (90:10) as an eluent, resulting in a white solid (0.1346 g, 90% yield, MP 229–231 °C).

The syntheses of aromatic and heterocyclic BA derivatives were performed as described [14,15,16].

The HPLC-DAD technique was applied for the analysis of all obtained derivatives, and their structures were confirmed by ^1^H, ^13^C, ^19^F NMR, and IR spectra.

### 2.4. ^1^H, ^13^C, ^19^F NMR, IR, and XRD Studies

^1^H, ^13^C, and ^19^F NMR spectra can be recorded, even for incompletely dissolved derivatives. However, selecting an appropriate solvent for the ^13^C spectrum was necessary to register signals of proper intensities, especially the signals of carbon atoms that have been split into multiplets due to heteronuclear spin–spin coupling with fluorine atoms. In the case of PAs-FNBT derivatives, the solvent was CDCl_3_, while Spd-FNBT was less soluble, and CD_3_CN was used. CNBF derivatives are much less soluble in CDCl_3_; hence, acetone-*d6* was applied. Because the derivatives of BPI have urea structures, only DMSO-*d6* or acetone-*d6* dissolved them.

The diffraction data of FNBT derivatives with Spm, Cad, and Put were collected at 100 K for the single crystal on a Rigaku XtaLAB Synergy diffractometer with a HyPix detector with a monochromated CuKα X-ray source (λ = 1.54184 Å). The data reduction, space group determination, and analytical absorption correction were performed with CrysAlis Pro [19]. The data set (Spd-CNBF) was collected at 100 K on an MX14-2 beamline (BESSY II synchrotron, Helmholtz Zentrum Berlin) using λ = 0.7999 Å radiation, and data reduction was performed in xdsapp [20]. All structures were solved by direct methods and refined with the full-matrix least-squares procedure on F (SHELX-97 [21]). All the heavy atoms were refined with anisotropic displacement parameters. Hydrogen atoms were located at calculated positions with the thermal displacement parameters fixed to a value of 20% higher than those of the corresponding carbon atoms. Hydrogen atoms from NH groups were obtained from the difference electron density synthesis. For Cad-FNBT in CrysAlis, two grains were detected and the data were processed for twinned system, and the refinement process was performed with an HKLF 5 flag. For this structure, several restraints (DFIX and ISOR) were applied on a rotationally disordered C18 CF_3_ group to assure reasonable geometry and stable refinement. In Spd-CNBF thermal restraints (ISORs) were applied on F10 and F11 atoms from the minor set (0.30) of a rotationally disordered CF_3_ group. All the figures were prepared in ORTEP-3 [22]. The results of the data collections and refinement are summarized in Table S1 (Supplementary Materials). Structural analysis was performed in CrystalExplorer 21.5 [23,24,25]. The interaction’s energy was calculated using the B3LYP/6-31G (d,p) procedure [26,27,28,29,30].

CCDC 2177543, 2177544, 2177545, and 2177548 contained the supplementary crystallographic data for the obtained derivatives. These data can be obtained free of charge from The Cambridge Crystallographic Data Centre via www.ccdc.cam.ac.uk/data_request/cif.

### 2.5. Chromatographic Analysis

All PA derivatives were determined using RP-HPLC, as reported by Liu et al. [31]. The mobile phase was acetonitrile (solvent A) and water (solvent B), and the gradient conditions were 0 min A: 60%; 40 min A: 100%; 47 min A: 100%; 48 min A: 60%, and 57 min A: 60%. The total flow rate was 1 mL/min; temperature: 35 °C; injection volume was 20 µL, and the detection wavelength was 254 nm. The chromatographic data were recorded and processed by the LC solution program, version 1.23 SP. The polyamine derivatives in wine samples were identified according to the retention times of the corresponding standards. Quantification was performed using the calibration curves of the respective standards.

### 2.6. Samples Preparation

The investigated nine home-made wines (six red and three white) were produced from grapevines (Cascade, Maréchal Foch, Agat Donskoy, Aurora, Arcadia); chokeberry, elderberry, and apple, and were produced in one household in 2021. The commercially available yeast (universal, Bordeaux, or enovini) for fermentation was applied. A potassium metabisulphite solution (3%) was used to disinfect the wine-making equipment and was added to yeast (1 g·10 L^−1^). All wines were made using crushed fruit (collected in October), except of apple wine, which was made from squeezed juice.

All the tested, home-made wines were prepared in one local household in central-eastern Poland. This region of Poland is dominated by a relatively mild climate, hilly terrain, and fertile loess soils, which provide suitable conditions for cultivating grapes and other fruits.

### 2.7. Selection of Sample Preparation Method

A sample preparation procedure, based on double extraction with a mixture of hexane, diethyl ether, and ethyl acetate, was used [14,16]. However, the procedure was time-consuming; hence, a simple sample preparation method based on the removal of the phenolic compounds with polysaccharides and purification after synthesis by flash chromatography was applied in this work.

The sample preparation stage was tested on samples of sweet, red, home-made wine (Cascade grapevine; sample 1). Wine samples were centrifuged (9000 rpm, 15 min) and filtrated. In the following stage, 10 mL of the sample was treated with 0.5 g of PVP, pectin, kappa-carrageenan, hide powder, or chitosan, respectively. The mixtures were shaken for 30 min in an orbital shaker, centrifuged, and filtrated. Derivatization reactions were performed in the same way as described above. Samples (5 mL) were treated with 1 mL of FNBT (or 0.5 g CNBF) and 1.5 mL of DIPEA, or in the case of BPI (0.5 mL), 2 mL of wine samples were used. The obtained products were mixed with silica gel (1.5 g), evaporated, and purified by flash column chromatography (C18 columns) with methanol/water eluent. After the solvents’ removal, the derivatives were dissolved in methanol (10 mL), filtered, and analyzed by HPLC-DAD.

### 2.8. Statistical Evaluation of Proposed Procedures

The HPLC procedure for PA derivatives was evaluated in terms of linearity, precision, accuracy, detection (DL), and quantification (QL) limits. The linear ranges of the curves for the obtained derivatives were recorded and converted into pure polyamine concentrations. All analyses were performed three times, and all the results are presented as mean ± standard deviation. Method intra-day precision and accuracy (recovery studies) were determined by five replicates’ analyses of three mixtures of biogenic amines at different concentration levels. The HPLC conditions, wavelength, and flow rate were deliberately altered to evaluate the robustness of the procedures developed. The wavelength was changed by ±2 nm and the flow rate by ±0.2 mL·min^−1^ while keeping the other chromatographic conditions constant. Recoveries (Rec) of the Pas’ determination were calculated by analyzing the selected wine samples, Cascade, sweet red wine (sample 1); Aurora, semi-sweet white wine (sample 6); elderberry wine (sample 7); chokeberry wine (sample 8); and apple wine (sample 9), spiked with polyamine standards (2.50 mg·L^−1^; 5.50 mg·L^−1^, and 10.50 mg·L^−1^). Moreover, the stability of spiked sample solutions after the derivatives reaction was evaluated in triplicate after 7 and 14 days of storage (4 °C). One-way ANOVA followed by Turkey’s multiple comparison tests were performed to determine the significant differences between the data (*p* < 0.05).

## 3. Results and Discussion

### 3.1. Structural Characteristics of the Obtained Derivatives

**Figure 1:**
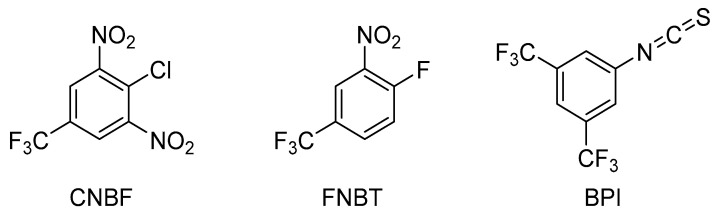
Structures of reagents used for the synthesis of biogenic amine derivatives.

The polyamines derivatives were obtained in reactions with FNBT, CNBF, and BPI, as reported (Figure 1) [14,15,16].

**Figure 2:**
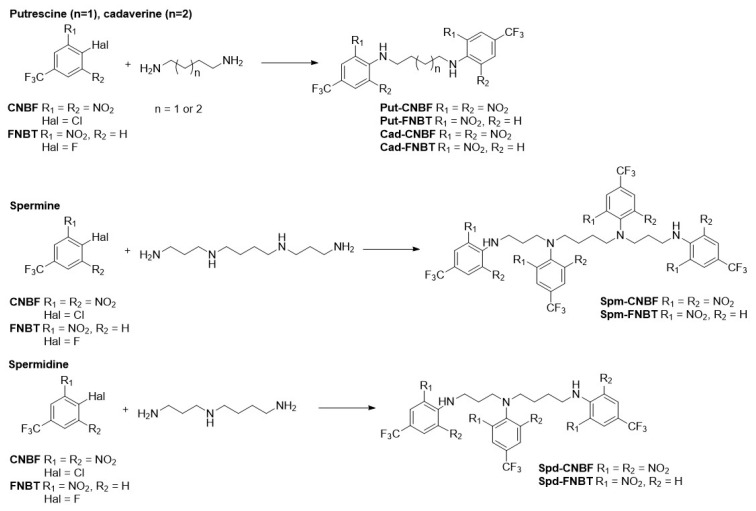
Reaction scheme of CNBF and FNBT with polyamines.

CNBF and FNBT readily undergo a nucleophilic aromatic substitution; hence, numerous publications describe the usage of these reagents to synthesize heterocyclic compounds for medical applications [32,33] and were also applied for the determination of biogenic amines. However, to the best of our knowledge, there is a lack of information on spermidine and spermine derivatives’ structures. We noticed that BPI is highly reactive towards amines and alcohols and requires a non-reactive solvent (such as tetrahydrofuran, THF). The first two reagents (FNBT and CNBF) differed in reactivity towards amines; however, the mechanism of their reaction with amines was analogous, and the products were structurally similar to each other (Figure 2).

On the other hand, BPI is an isothiocyanate, willingly reacting with amines to produce thioureas. Therefore, we discussed the analysis of these derivatives separately. The structures of all obtained compounds were confirmed by ^1^H, ^13^C, ^19^F NMR, and FTIR ATR analyses. The full description of the ^1^H, ^13^C, ^19^F NMR, and FTIR-ATR spectra of polyamine derivatives is presented in the Supplementary Materials.

The structure of the studied polyamines is a feature that clearly distinguishes them. Thus, Put and Cad have only terminal primary amino groups. In contrast, spermidine and spermine have, in addition to primary amino groups, one and two secondary amino groups, respectively. Additionally, Put, Cad, and Spm are symmetrical, unlike spermidine, which is nonsymmetric. These structural differences were also observed in the NMR spectra of the individual amine derivatives. Based on the number of signals and the integration of the areas of particular peaks in the ^1^H NMR spectra, the symmetrical substitution of the Put and Cad amino groups with phenyl residues from FNBT and CNBF was unequivocally confirmed. The analysis of the spectra of FNBT and CNBF derivatives of spermine and spermidine became more interesting. The substitution of the amino groups in symmetric spermine with four phenyl substituents resulted in the appearance of two types of signals from aromatic groups in the ^1^H and ^13^C spectra. In addition, the analysis of the ^19^F NMR spectrum of the spermine FNBT derivative confirmed the tetrasubstituted product. Two peaks with the same integration areas were present in the spectrum. The peak with a chemical shift of −62.97 ppm (CDCl_3_) corresponded to the CF_3_ groups in the terminal aryl substituents, as was the case for Put-FNBT (−62.93 ppm). The second signal, with a chemical shift of −63.11 ppm, was obtained from CF_3_ groups in aryl substituents attached to internal nitrogen atoms. In the ^19^F NMR spectrum of the CNBF derivative of spermine, there were also two signals analogous to Spm-FNBT.

Spermidine is a triamine in which nitrogen atoms are linked together by a propyl and butyl linker. An additional methylene group in the butyl fragment causes asymmetry, and as a result of attaching aryl residues to nitrogen atoms, each of these groups can be observed separately. For example, Spd-FNBT in the ^19^F spectrum produced three signals for the sample recorded in THF-*d8* (−63.64, −63.60, −63.57 ppm), although the sample dissolved in acetonitrile-*d3* produced two peaks with an area ratio of 2:1: −63.35 and −63.32 ppm, respectively. Similarly, in the ^13^C NMR spectra, both for Spd-FNBT and Spd-CNBF, all carbon atoms of the alkyl part and phenyl groups were visible, as well as the characteristic quartet signals resulting from the couplings between three fluorine and carbon atoms.

Additionally, the structures of aliphatic PAs derivatives were confirmed by IR spectra. For CNBF and FNBT derivatives, characteristic bands for the nitro groups were visible (1351.30–1365.44 cm^−1^ and 1523.81–1541.42 cm^−1^ for CNBF-Pas, and 1316.72–1322.24 cm^−1^ and 1525.96–1536.29 cm^−1^ for FNBT-PAs, respectively) and trifluoromethyl groups (1129.58–1167.78 cm^−1^ for CNBF-PAs and 1126.91–1182.98 cm^−1^ for FNBT-PAs, respectively).

The obtained derivatives were crystallized, and only four products produced crystals suitable for X-ray study. The obtained structures revealed that all NH_2_ groups, four for Spm, two for Put, and Cad, were involved in the reaction with FNBT, whereas three amine groups were produced for Spd with CNBF. The molecular structures of the obtained polyamine derivatives are presented in the Supplementary Materials (Figure S1).

**Figure 3:**
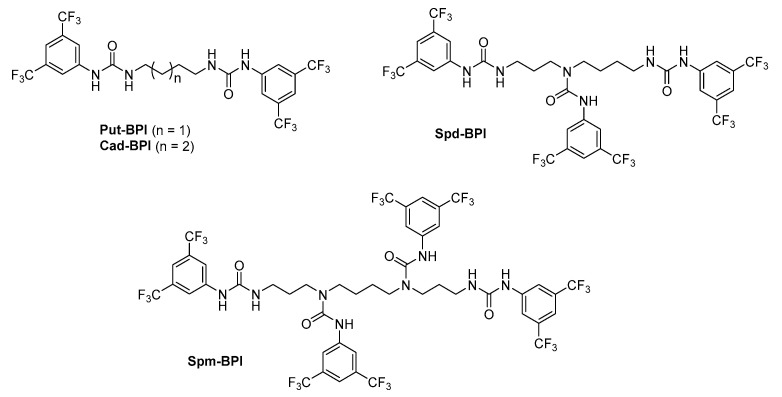
Urea derivatives obtained in the reaction of BPI with polyamines.

BPI (3,5-bis (trifluoromethyl) phenyl isothiocyanate) reacted similarly to FNBT and CNBF, with all the amine groups present in the polyamine molecules. An analysis of the ^1^H, ^13^C, and ^19^F NMR spectra confirmed the formation of fully substituted compounds. Unfortunately, for the obtained BPI derivatives with polyamines, the characteristic signals of the thiourea groups (C=S) with a chemical shift of about 182 ppm were not observed. All polyamines reacted with BPI to form urea groups with a C=O moiety, with chemical shifts in the range of 154.43–155.87 ppm (Figure 3). The desulfurization reactions of thioureas to ureas are known, but usually require the participation of oxidants; although, reactions with oxygen are also known [34].

The FTIR-ATR spectra of PA-BPI derivatives confirmed characteristic bands for the carbonyl group in the urea system (1640–1655 cm^−1^) and the C–N bond (1472–1473 cm^−1^), as well as for the trifluoromethyl groups (1169–1172 cm^−1^).

This was surprising due to the advantages of the isothiocyanate reagent for the simple and convenient derivatization of the aromatic or heterocyclic amines previously described [16]. Considering the different reactivity levels of isothiocyanate with biogenic amines and polyamines leading to thioureas and ureas, respectively, we did not pursue further studies using this derivatizing reagent.

### 3.2. RP-HPLC Method Development and Validation

The regression parameters of the calibration curves for the tested PAs are listed in Table 1. The results of the accuracy analysis for the tested concentrations of polyamines are summarized in Table S2 (Supplementary Materials).

The measured retention times of the PA derivatives indicate the adequate separation of the analytes. The reproducibility of the retention time analysis, expressed by the intra-day coefficient of variation (CV), was satisfactory and ranged from 0.07% to 0.21% (CNBF procedure) and from 0.04% to 0.19% (FNBT procedure), respectively. The determined results regarding the linearity of the method, including its coefficient of determination, as well as limits of detection (DL) and quantification (QL), suggest the robust linearity of the tested concentration range for all considered derivatives.

The CV values for the obtained concentrations of PA derivatives were in the range of 1.09–3.27% (CNBF procedure) and 1.08–1.29% (FNBT procedure), indicating satisfactory intra-day reproducibility. The accuracy of the proposed procedures was tested by the recovery studies of the PA standard solution, and the average recovery index varied between 93.88–98.18% (CNBF procedure) and 96.96–98.27% (FNBT procedure). Table 1 indicates that the precision and accuracy of the proposed procedure were satisfactory; however, better results were obtained for FNBT.

The robustness of the proposed procedures was assessed with respect to the change in wavelength (±2 nm), a flow rate of the mobile phase (±0.2 mL·min^−1^), and the chromatographic separation of PA derivatives with CNBF and FNBT. The values of the QL for all tested compounds were identical to those listed in Table 1, whereas the CV values for the time of migration and area of the obtained peaks were below 0.3%. These data indicate that the developed procedures are robust regarding the changes in the wavelength and flow rate of the mobile phase.

### 3.3. The Determination of the PA Derivatives in Home-Made Wine Samples

#### 3.3.1. Sample Preparation

To solve the problem of polyphenol interference occurring in the wine samples, the selected polysaccharides, such as kappa-carrageenan, pectin, and chitosan, were tested, and the obtained results were compared with the polyvinylpyrrolidone (PVP) purification procedure. Moreover, hide powder was tested for the pre-purification of the wine samples, which was applied to prevent vegetable extracts from producing enzyme-hydrolyzing proteins that moderately degrade peptones and peptides. Unfortunately, the use of the hide powder did not allow for the satisfactory removal of compounds interfering with the obtained derivatives. In this study, the best results for wine sample purification were obtained for chitosan. It is a highly biocompatible, non-toxic compound used as a perfect sorbent in water purification from heavy metal contamination. Moreover, it is known as a pseudo-natural cationic polymer, and thus, it finds many applications as a flocculent, clarifier, thickener, or affinity chromatography column matrix [35]. Despite its wide use in cosmetics, food, medicine, polymers, or biocatalysis, according to our literature study, chitosan has not yet been used to remove interfering compounds from wines during PA analysis. Its use allowed us to obtain statistically similar results (one-way ANOVA, *p* < 0.05) for the determination of PAs in house wine compared to the procedure with PVP (Figure S2, Supplementary Materials).

It should be noted that PVP is a very popular additive in pharmaceutical, cosmetic, and packaging materials. Because this compound has very low toxicity, it can also be used in the food industry as a stabilizer or thickening agent. This popularity and the resistance of PVP to biodegradation are associated with an excessive amount of this compound in the environment [36]. The main advantages of chitosan are high biocompatibility, biodegradability, and availability. Therefore, it seemed reasonable to use chitosan to precipitate of phenolic compounds in the tested wines.

#### 3.3.2. Wine Sample Analysis

The proposed procedures for the sample preparation (PA derivatives’ synthesis with CNBF, FNBT, and sample purification) was applied before the determination of the polyamines in the tested home-made wine samples, and the obtained results are listed in Table 2.

Considering the factors that have an impact on the level of biogenic amines in wines, the most important factors seem to be agronomical practices, amino acid content, variety, and degree of grapes’ ripeness [37]. According to Ancín-Azpilicueta et al. [38], Put, Cad, Spm, and Spd were frequently observed in wines. In this study, the predominant polyamine in all the home-made wine samples appeared to be putrescine (Table 1). High amounts of this polyamine were observed in red wines, which can be related to secondary fermentation (malolactic fermentation). Moreover, red wine vinification was conducted in the presence of grape skin and pulp, and putrescine could be released from them into the must. Additionally, Put can also originate from the microbial decarboxylation of ornithine or arginine via agmatine [38]. On the other hand, high levels of this polyamine in fermented food indicate inappropriate sanitary conditions during food processing [39]. According to [38], Put could reduce the sensory quality at 15–20 and 20–30 mg·L^−1^ in white and red wines, respectively. Based on the obtained results, samples 1 (Cascade, sweet red wine), 3 (Agat Donskoy, sweet red wine), and 4 (Agat Donskoy, sweet red wine) contain the discussed compound at a level that may lower the accepted taste of wine. Similarly, a noticeably high level of Cad in red wines (except for sample 2, Table 2) was observed. It should be added that Put and Cad, although not toxic themselves, can affect the action of MAO and DAO, which are responsible for the degradation of Him, Tym, and Phe, and consequently, they can increase the toxic activity of these amines.

The remaining polyamines were observed in the studied samples at a much lower level. The Spm and Spd in wine can originate from grapes and/or from yeast lysis; however, their level was significantly decreased during winemaking due to the growth of lactic acid bacteria or potential consumption by alcoholic fermentative yeasts, or due to spontaneous chemical degradation.

By comparing the results obtained by one-way ANOVA, followed by the Duncan test, it is evident that the mean value of PAs in the majority of the wine samples reveal significant differences. The latter can be related to FNBT selectivity toward the polyamines because this reagent only reacts with amine groups, thus promoting the selective extraction of N-2′-nitro-4-trifuoromethylphenyl polyamine (NTP) derivatives to an organic phase [3]. Comparing the results presented in Table 1, it can be noted that the Spd-FNBT derivative allows for the determination of Spd at a very low level: 0.03 mg·L^−1^ for red, sweet wine (Maréchal Foch) and 0.02 mg·L^−1^ for elderberry wine. Although FNBT derivatives have been used for the determination of polyamines in plant extracts and clinical samples [3], this reagent is not widely used in food analysis.

A standard addition method was applied for the evaluation of the proposed procedures’ accuracy. Recoveries of spiked samples (obtained by adding known amounts of PAs to the blank matrix) were calculated, and the results are presented in Table 3.

The mean values of recovery for the CNBF procedure varied from 93.52% (Spd) to 96.32% (Put), whereas for FNBT, one varied from 95.74% (Spd) to 97.19% (Put). Surprisingly, the lowest accuracy for both reagents was presented for spermidine, while the best was for putrescine. It is evident that the procedure using FNBT derivatives is characterized by better accuracy.

A similar situation was observed for precision expressed as a values of variation coefficient. The differences in reactivity with various reagents may result from a steric hindrance in nitrogen atoms in the polyamines and halogen atoms in CNBF and FNBT. Furthermore, the presence of the CF_3_ group in FNBT influenced the reactivity of the discussed reagent and the hydrophilicity of the obtained product. It is worth noting that for the discussed procedure with FNBT, the recovery values did not change over time (7 and 14 days), which suggests the satisfactory accuracy and stability of the proposed method and obtained derivatives. The structural analysis confirmed the latter, which was discussed for the Put-FNBT example. This compound crystallizes in the triclinic P-1 space group with half of the molecule in the asymmetric unit (Figure S1, Supplementary Materials). The molecule in the solid state adopts an elongated conformation with all bonds in anti-positions in an aliphatic linker (torsion angles: N7-C8-C9-C9[1 − x, 2 − y, 2 − z] −179.98, C8-C9-C9[1 − x, 2 − y, 2 − z]-C8[1 − x, 2 − y, 2 − z] −180.0, C9 − C9 − C8 − N7 −179.98°). In the FNBT moiety, intramolecular N7-H7…O1 and N7-H7…N5 hydrogen bonds were observed. In the crystal network, there are formed columns along the *a* axis, mainly maintained due to stacking interactions between C6 phenyl rings (Figure S3, Supplementary Materials). However, the energy landscape shows that there are two other important interactions with molecules from adjacent pillars (Figure S4, Supplementary Materials). For [x, y, z] and [−x, 1 − y, 2 − z] molecules, the electrostatic factor is important. The interactions projected onto the Hirshfeld surface show that H…O contacts are the most abundant; however, there are no spikes corresponding to short and strong hydrogen bonds (Figure S5, Supplementary Materials). Hence, in the crystal network, weak dispersion interactions prevail. It should be noted that the high percentage of F…F interactions related to mutual CF_3_ group positions from adjacent molecules.

Based on the obtained results, FNBT is the best choice for the determination of polyamines in the tested food samples. To the best of our knowledge, this is the first validated procedure of PAs determination in home-made wine preceding pre-column derivatization with FNBT.

The determination of both polyamines and biogenic amines in food samples is still an interesting analytical problem. Multiple analytical methods have been developed to determine these compounds in various foods and beverages. Many review papers have been written in recent years [40,41,42]. Chromatographic methods of PA determination feature a pre- or post-column derivatization step because many amines do not absorb in the UV region. Moreover, this process can be conducted either off- or on-line. Recently, post-column derivatization coupled with the HPLC method was proposed as a powerful tool in the modern analytical laboratory [43,44]. This technique has many advantages, such as avoiding matrix interference effects, increasing the tested compounds’ signal, or detecting compounds that are otherwise undetectable with available detectors. Moreover, it offers lower sample manipulation, high sensitivity, and selectivity. Among others, reagents, such as *o*-phtalaldehyde, dansyl-Cl, or 1,2-naphthoquinone-4-sulfonate have been used for this purpose. The post-column derivatization step for determining biogenic amines, including PAs in wines, was described [45,46]. However, this technique is less popular for PA determination than the pre-column process, because the post-column procedure requires more complex equipment since an additional pump is needed for the post-column reagent. Regardless of the number of derivatization procedures described, the off-line pre-column derivatization method is still widely used because it is simple and has a low-cost factor and does not require other equipment. Moreover, the possible interferences from the matrix can be removed prior to separation, and the obtained derivatives can be separated on reverse phase columns using mobile phases compatible for UV-Vis detection.

The broad array of derivatizing reagents was proposed and discussed in this study. The selection should consider, among others, the necessity to scavenge excess reagents, the instability of the obtained products, the formation of by-products, the lack of selectivity, and the reaction time. Therefore, in recent years, new derivatization strategies have been proposed for the more rapid, accurate, and uncomplicated detection of these compounds in food. As proposed in this work, the reaction with FNBT was simple, effective, and the obtained derivatives were stable during 14 days of storage (Table 3). The latter can be related to the presence of the electron-withdrawing groups in FNBT, resulting in the activation of the fluorine atom for a nucleophilic substitution reaction with amines. Therefore, FNBT only reacts with amino groups, thus promoting the selectivity of the procedure.

### 3.4. The Determination of the BAs in Home-Made Wine Samples

The obtained results for the determination of remaining biogenic amines in wine samples following derivatization with FNBT are presented in Tables S3 and S4 (Supplementary Materials).

A higher level of BAs in red than in white wine may be caused by the maceration process of the grape skins, which occurs only in red wine production. This produces high amounts of free amino acids and a high fermentation temperature, together with malolactic fermentation (MLF). This process of vinification in white and other wine production processes was performed over a much shorter time. The histamine content in the tested red home-made wines (samples 1–4) was particularly disturbing. This amine is known to cause headaches, low blood pressure, heart palpitations, vomiting, and diarrhea. Together with tyramine, phenylethylamine revealed toxic effects on the organism. Moreover, we can conclude that controlling the content of biogenic amines in home-made wines is essential.

At present, the production of wine with a low biogenic amine content is a worldwide concern. In this context, the obtained results show that during the home-made vinification process, some factors that affected the concentration of BAs were not completely controlled. Usually, fruit wines are characterized by a low BA concentration, compared to the red ones [45]. This fact can be related to fruit winemaking processes not involving malolactic fermentation. The same situation was observed for elderberry, chokeberry, and apple wines. The amount of polyamines, Him, Phe, and Tym in these samples was similar to white grape wine, whereas Try was not detected. For comparison, Płotka-Wasylka et al. [18] analyzed several fruit wines produced from different types of fruits, such as apple, black lilac, and quince. They observed Him, Put, Cad, dimethylamine, methylamine, ethylamine, and butylamine at different concentrations, based on the fruits used for their production. Interestingly, elderberry and apple wines had similar concentrations of all BAs, whereas, in chokeberry wine, the BA level was significantly lower. For comparison, Nalazek-Rudnicka and Wasik [47] determined selected BAs in different fruit wines; however, they were not detected in chokeberry. Some authors [38,40] suggested that, in the case of wines, the toxic impact of BAs is not connected to their high concentration, but to the consumption of large quantities of wine in a very short time.

## 4. Conclusions

We compared the applicability of CNBF and FNBT, which react with amines in the analogous mechanism; however, with different reactivity values, BPI reacts with amines to produce thioureas or ureas. It was confirmed that CNBF and FNBT resulted in satisfactory results for the determination of polyamines. The results of the analyses and conditions of derivative synthesis reveal that FNBT is the best choice for the discussed compounds.

It should be added that despite numerous efforts to determine biogenic amines in food, their isolation, separation, and analysis from complex biological matrices is still a challenging analytical problem. One of the basic rules of the modern analytical method demands a minimal number of stages. In this context, the advantages of the proposed procedure for polyamine determination are the elimination of the laborious process of sample extraction, synthesis of derivatives at room temperature, and lack of reagent interference.

In conclusion, we suggest simple and sensitive procedures for PA (putrescine, cadaverine, spermine, and spermidine) determination in home-made wine samples. Moreover, a simple sample preparation procedure was described, based on wine sample purification using chitosan and flash column chromatography.

The production of wine with a low biogenic amine content is a worldwide concern; however, the regulatory limits for these compounds in wine have not yet been established. Our PA determination results for food samples reveal detectable levels of the discussed compounds, especially putrescine. The latter fact indicates some problems in home-made wine production, e.g., the lack of vinification practices, such as using bentonite to eliminate the growth of bacteria that produce high concentrations of amines or inoculate starter cultures without amino acid decarboxylase activity, in order to reduce PA content. This suggests that the development of a simple procedure for biogenic amines’ determination is worth studying.

## Figures and Tables

**Table 1:** Validation parameters of PA derivatives’ determination by RP-HPLC method.

	Put	Cad	Spd	Spm
CNBF				
Range [mg·L^−1^]	4.00–31.00	2.00–22.00	2.00–20.00	2.00–24.00
t [min]	18.44	19.38	24.51	28.74
Mean CV_t_ [%]	0.11	0.21	0.16	0.07
R^2^	0.9997	0.9990	0.9992	0.9990
DL [mg·L^−1^]	0.10	0.12	0.15	0.12
QL [mg·L^−1^]	0.33	0.40	0.51	0.41
Mean Rec [%]	98.18	95.54	93.88	94.59
Mean CV [%]	1.09	2.57	1.65	3.27
FNBT				
Range [mg·L^−1^]	2.00–16.00	2.00–18.00	2.00– 0.00	2.00–18.00
t [min]	20.11	22.87	27.95	33.08
Mean CV_t_ [%]	0.10	0.04	0.07	0.06
R^2^	0.9999	0.9999	0.9990	0.09995
DL [mg·L^−1^]	0.10	0.17	0.15	0.19
QL [mg·L^−1^]	0.30	0.58	0.51	0.62
Mean Rec [%]	98.27	97.51	96.96	98.00
Mean CV [%]	1.22	1.15	1.08	1.29

Abbreviations: Put—putrescine; Cad—cadaverine; Spm—spermine; Spd—spermidine; t—retention time; SD—standard deviation; R^2^—coefficient of determination; n—number of samples; DL—detection limit [mg·L^−1^] = (3 × s_y/x_) × b^−1^; QL—quantification limit [mg·L^−1^] = (10 × s_y/x_) × b^1−^; Rec—recovery was calculated as mean value of (experimental/obtained concentration)·100%; CV—coefficient of variation [%].

**Table 2:** Results of polyamine determination (**X ± SD) [mg·L^−1^]** in wine samples after derivatization with CNBF and FNBT.

	Put-CNBF	Put-FNBT	Cad-CNBF	Cad-FNBT	Spd-CNBF	Spd-FNBT	Spm-CNBF	Spm-FNBT
sample 1	25.5 ^a,^ _g_ ±0.3	26.4 ^b,^ _i_ ±0.2	15.3 ^b,^ _g_ ±0.4	14.9 ^a,^ _g_ ±0.3	0.059 ^a,^ _a_ ±0.005	0.076 ^b,^_a_ ±0.004	0.11 ^a,^ _b_ ±0.02	0.13 ^b,^ _a,b_ ±0.01
sample 2	16.3 ^a,^ _d_ ±0.1	17.3^b,^ _f_ ±0.2	5.75^a,^_e_ ±0.03	6.30 ^b,^ _f_ ±0.07	nd	0.03_a_ ±0.01	nd	nd
sample 3	21.1 ^a,^ _f_ ±0.2	21.3 ^a,^ _h_ ±0.1	14.5 ^a,^ _f_ ±0.3	14.9^b,^_g_ ±0.1	9.93 ^b,^_f_ ±0.04	9.80 ^a,^ _f_ ±0.17	2.86 ^a,^_e_ ±0.05	2.85 ^a,^ _c_ ±0.11
sample 4	18.7 ^b,^ _e_ ±0.1	18.2 ^a,^ _g_ ±0.3	15.8 ^b,^ _h_ ±0.1	14.7 ^a,^ _g_ ±0.2	2.35 ^a,^_e_ ±0.04	2.79 ^b,^ _e_ ±0.03	nd	nd
sample 5	9.15 ^a,^ _c_ ±0.20	10.6 ^b,^ _e_ ±0.1	3.48 ^a,^ _c_ ±0.18	3.86 ^b,^ _d_ ±0.02	0.13 ^a,^_a_ ±0.02	0.26 ^b,^ _b_ ±0.01	0.20 ^a,^ _c_ ±0.02	0.20 ^a,^ _b_ ±0.01
sample 6	5.15 ^a,^ _b_ ±0.13	5.36 ^b,^ _c_ ±0.02	1.91 ^a,^ _b_ ±0.01	2.07 ^a,^ _c_ ±0.01	0.42 ^a,^ _b_ ±0.02	0.66 ^b,^ _c_ ±0.01	2.51 ^a,^ _d_ ±0.02	2.82 ^b,^ _c_ ±0.11
sample 7	9.19 ±0.18 ^a,^ _c_	9.85 ±0.04 ^b,^ _d_	5.07 ±0.05 ^a,^ _d_	5.37 ±0.04 ^b,^ _e_	nd	0.02 ±0.01 _a_	nd	nd
sample 8	4.78 ^a,^ _a_ ±0.02	5.10 ^b,^ _a_ ±0.12	1.70 ^a,^ _a,b_ ±0.01	1.82 ^b,^ _b_ ±0.01	1.67 ^a,^ _c_ ±0.09	2.04 ^b,^ _d_ ±0.02	0.094 ^a,^ _a_ ±0.007	0.095 ^a,^ _a_ ±0.004
sample 9	5.21 ^a,^ _b_ ±0.03	5.50 ^b,^ _b_ ±0.07	1.47 ^a,^ _a_ ±0.01	1.55 ^b,^ _a_ ±0.03	1.89 ^a,^ _d_ ±0.01	1.97 ^b,^ _d_ ±0.02	0.011 ^a,^ _b_ ±0.001	0.014 ^b,^ _a_ ±0.002

Abbreviations: X—mean value; SD—standard deviation, number of independent samples = 3; nd—not detected. Different red letters (a–b) within the same amine indicate significant differences (one-way ANOVA and Duncan test, *p* < 0.05) for tested reagents; different blue letters (a–h) within the same column indicate significant differences (one-way ANOVA and Duncan test, *p* < 0.05) for tested samples; sorted from the lowest to highest values, where “a” is the lowest; sample 1—Cascade, sweet red wine; sample 2—Maréchal Foch, sweet red wine; sample 3—Agat Donskoy, sweet red wine (Enovini yeast); sample 4—Agat Donskoy, sweet red wine (Bordeaux yeast); sample 5—Aurora and Arcadia, sweet white wine; sample 6—Aurora, semi-sweet white wine; sample 7—elderberry wine; sample 8—chokeberry wine; sample 9—apple wine.

**Table 3:** The recovery values for the assay of PAs in real samples (*n* = 5).

PAs	Added [mg·L^−1^]	CNBF				FNBT			
		Rec [%]	CV [%]	Mean Rec after 7 Days ±SD [%]	Mean Rec after 14 Days ±SD [%]	Rec [%]	CV [%]	Mean Rec after 7 Days ±SD [%]	Mean Rec after 14 Days ±SD
Put	2.50	95.40	3.14	95.06 ± 4.16	94.11 ± 3.31	96.52	2.89	95.59 ± 3.13	95.34 ± 3.07
	5.50	96.36	3.91			96.80	3.23		
	10.50	97.21	1.53			98.25	1.26		
Cad	2.50	95.07	3.05	94.06 ± 3.08	92.07 ± 2.71	95.79	2.32	95.38 ± 3.03	95.16 ± 3.61
	5.50	94.47	3.54			95.86	2.86		
	10.50	95.76	2.96			97.22	1.72		
Spd	2.50	92.63	3.39	93.76 ± 3.72	92.60 ± 0.35	95.17	2.11	96.52 ± 2.89	95.30 ± 4.52
	5.50	93.41	3.80			95.25	2.69		
	10.50	94.53	3.27			96.80	2.36		
Spm	2.50	92.47	3.39	93.96 ± 3.81	92.63 ± 4.18	95.25	2.58	96.04 ± 2.55	95.23 ± 3.16
	5.50	96.49	2.23			97.08	1.80		
	10.50	94.67	4.07			96.24	1.99		

Abbreviations: Rec—recovery calculated as mean value of [((X_2_ − X_0_)/X_1_)·100%]; X_0_—concentration found in the blank sample; X_2_—concentration found in spiked sample; X_1_—added concentration of PAs; CV—coefficient of variation [%]; n—number of samples, each sample was analyzed in triplicate.

## Data Availability

Not applicable.

## Supplementary Materials

The following supporting information can be downloaded at https://www.mdpi.com/article/10.3390/ma16041474/s1: physicochemical data for all obtained polyamine derivatives, including yields, melting points, NMR and IR spectra descriptions, and the selected ^1^H, ^13^C, ^19^F NMR, and IR spectra, are presented. Table S1. The results of the data collections and refinement for Cad-FNBT, Put-FNBT, Spm-FNBT, and Spd-CNBF; Table S2. Precision and accuracy of the proposed procedures; Table S3. Linear regression calibration parameters of BAs determination by RP-HPLC method after FNBT derivatization; Table S4. Results of aromatic and heterocyclic biogenic amines’ determination (X ± SD) [mg·L^−1^] in tested wine samples; Figure S1. Molecular structures of polyamine derivatives; Figure S2. Results of biogenic amines’ chromatographic determination in wine samples after FNBT derivatization (sample 1, Cascade, red sweet wine) and purification procedures with chitosan and PVP; Figure S3. Packing of Put-FNBT along *a* axis; Figure S4. Hirshfeld surfaces and fingerprints of selected interactions created in the crystal network of Put-FNBT; Figure S5. Interaction energy (total—a, electrostatic—b, dispersion—c) in the crystal network of Put-FNBT are presented in kJ/mol. The tube diameter depends on the interaction energy. CRediT authorship contribution statement.
