# Supplementary material for: The Selection of the Best Derivatization Reagents for the Determination of Polyamines in Home-Made Wine Samples
Source: Materials (Basel). 2023 Feb 9;16(4):1474. doi: 10.3390/ma16041474 (PMC9960030; doi:10.3390/ma16041474)

checkCIF/PLATON report

Structure factors have been supplied for datablock(s) naj5-3Dtw2shape_twin1_hklf5

THIS REPORT IS FOR GUIDANCE ONLY. IF USED AS PART OF A REVIEW PROCEDURE FOR PUBLICATION, IT SHOULD NOT REPLACE THE EXPERTISE OF AN EXPERIENCED CRYSTALLOGRAPHIC REFEREE.

No syntax errors found. CIF dictionary Interpreting this report

Datablock: naj5-3Dtw2shape_twin1_hklf5

```
Bond precision:   C-C = 0.0039 A          Wavelength=1.54184

Cell:           a=9.93377(12)    b=18.5968(3)        c=11.49761(16)
                alpha=90         beta=100.4050(13)   gamma=90
Temperature:    100 K
```

	Calculated	Reported
Volume	2089.10(5)	2089.10(5)
Space group	P 21/n	P 21/n
Hall group	-P 2yn	-P 2yn
Moiety formula	C19 H18 F6 N4 O4	C19 H18 F6 N4 O4
Sum formula	C19 H18 F6 N4 O4	C19 H18 F6 N4 O4
Mr	480.37	480.37
Dx,g cm-3	1.527	1.527
Z	4	4
Mu (mm-1)	1.267	1.267
F000	984.0	984.0
F000'	988.23	
h,k,lmax	12,23,14	12,23,14
Nref	4277	8183
Tmin,Tmax	0.872,0.945	0.890,0.951
Tmin'	0.870	

```
Correction method= # Reported T Limits: Tmin=0.890 Tmax=0.951
AbsCorr = ANALYTICAL

Data completeness= 1.913          Theta(max)= 74.465

R(reflections)= 0.0710( 6348)                    wR2(reflections)=
                                                 0.2197( 8183)
S = 1.054                  Npar= 326
```

The following ALERTS were generated. Each ALERT has the format
test-name_ALERT_alert-type_alert-level.
Click on the hyperlinks for more details of the test.

Alert level C

```
PLAT029_ALERT_3_C _diffrn_measured_fraction_theta_full value Low .      0.967 Why?
PLAT906_ALERT_3_C Large K Value in the Analysis of Variance ......      3.621 Check
PLAT911_ALERT_3_C Missing FCF Refl Between Thmin & STh/L=    0.600        125 Report
PLAT918_ALERT_3_C Reflection(s) with I(obs) much Smaller I(calc) .         12 Check
PLAT939_ALERT_3_C Large Value of Not (SHELXL) Weight Optimized S .      10.89 Check
```

Alert level G

```
PLAT002_ALERT_2_G Number of Distance or Angle Restraints on AtSite          7 Note
PLAT003_ALERT_2_G Number of Uiso or Uij Restrained non-H Atoms ...          7 Report
PLAT007_ALERT_5_G Number of Unrefined Donor-H Atoms .............           2 Report
PLAT172_ALERT_4_G The CIF-Embedded .res File Contains DFIX Records          1 Report
PLAT186_ALERT_4_G The CIF-Embedded .res File Contains ISOR Records          2 Report
PLAT230_ALERT_2_G Hirshfeld Test Diff for    F5       --C18      .        5.7 s.u.
PLAT230_ALERT_2_G Hirshfeld Test Diff for    F6       --C18      .        5.3 s.u.
PLAT242_ALERT_2_G Low    'MainMol' Ueq as Compared to Neighbors of        C3A Check
PLAT242_ALERT_2_G Low    'MainMol' Ueq as Compared to Neighbors of        C18 Check
PLAT300_ALERT_4_G Atom Site Occupancy of F4         Constrained at        0.7 Check
PLAT300_ALERT_4_G Atom Site Occupancy of F5         Constrained at        0.7 Check
PLAT300_ALERT_4_G Atom Site Occupancy of F6         Constrained at        0.7 Check
PLAT300_ALERT_4_G Atom Site Occupancy of F7         Constrained at        0.3 Check
PLAT300_ALERT_4_G Atom Site Occupancy of F8         Constrained at        0.3 Check
PLAT300_ALERT_4_G Atom Site Occupancy of F9         Constrained at        0.3 Check
PLAT301_ALERT_3_G Main Residue  Disorder ..............(Resd  1  )         9% Note
PLAT860_ALERT_3_G Number of Least-Squares Restraints .............         48 Note
PLAT870_ALERT_4_G ALERTS Related to Twinning Effects Suppressed ..          ! Info
PLAT883_ALERT_1_G No Info/Value for _atom_sites_solution_primary .     Please Do !
PLAT898_ALERT_4_G Second Reported H-M Symbol in CIF Ignored ......          ! Check
PLAT910_ALERT_3_G Missing # of FCF Reflection(s) Below Theta(Min).          1 Note
PLAT912_ALERT_4_G Missing # of FCF Reflections Above STh/L=  0.600         22 Note
PLAT933_ALERT_2_G Number of HKL-OMIT Records in Embedded .res File          1 Note
PLAT941_ALERT_3_G Average HKL Measurement Multiplicity ..........         2.0 Low
PLAT965_ALERT_2_G The SHELXL WEIGHT Optimisation has not Converged     Please Check
PLAT992_ALERT_5_G Repd & Actual _reflns_number_gt Values Differ by          2 Check
```

0 **ALERT level A** = Most likely a serious problem - resolve or explain
0 **ALERT level B** = A potentially serious problem, consider carefully
5 **ALERT level C** = Check. Ensure it is not caused by an omission or oversight
26 **ALERT level G** = General information/check it is not something unexpected

1 ALERT type 1 CIF construction/syntax error, inconsistent or missing data
8 ALERT type 2 Indicator that the structure model may be wrong or deficient
9 ALERT type 3 Indicator that the structure quality may be low
11 ALERT type 4 Improvement, methodology, query or suggestion
2 ALERT type 5 Informative message, check

It is advisable to attempt to resolve as many as possible of the alerts in all categories. Often the minor alerts point to easily fixed oversights, errors and omissions in your CIF or refinement strategy, so attention to these fine details can be worthwhile. In order to resolve some of the more serious problems it may be necessary to carry out additional measurements or structure refinements. However, the purpose of your study may justify the reported deviations and the more serious of these should normally be commented upon in the discussion or experimental section of a paper or in the "special_details" fields of the CIF. checkCIF was carefully designed to identify outliers and unusual parameters, but every test has its limitations and alerts that are not important in a particular case may appear. Conversely, the absence of alerts does not guarantee there are no aspects of the results needing attention. It is up to the individual to critically assess their own results and, if necessary, seek expert advice.

Publication of your CIF in IUCr journals

A basic structural check has been run on your CIF. These basic checks will be run on all CIFs submitted for publication in IUCr journals (*Acta Crystallographica*, *Journal of Applied Crystallography*, *Journal of Synchrotron Radiation*); however, if you intend to submit to *Acta Crystallographica Section C* or *E* or *IUCrData*, you should make sure that full publication checks are run on the final version of your CIF prior to submission.

Publication of your CIF in other journals

Please refer to the *Notes for Authors* of the relevant journal for any special instructions relating to CIF submission.

PLATON version of 19/02/2022; check.def file version of 19/02/2022

Datablock naj5-3Dtw2shape_twin1_hklf5 - ellipsoid plot

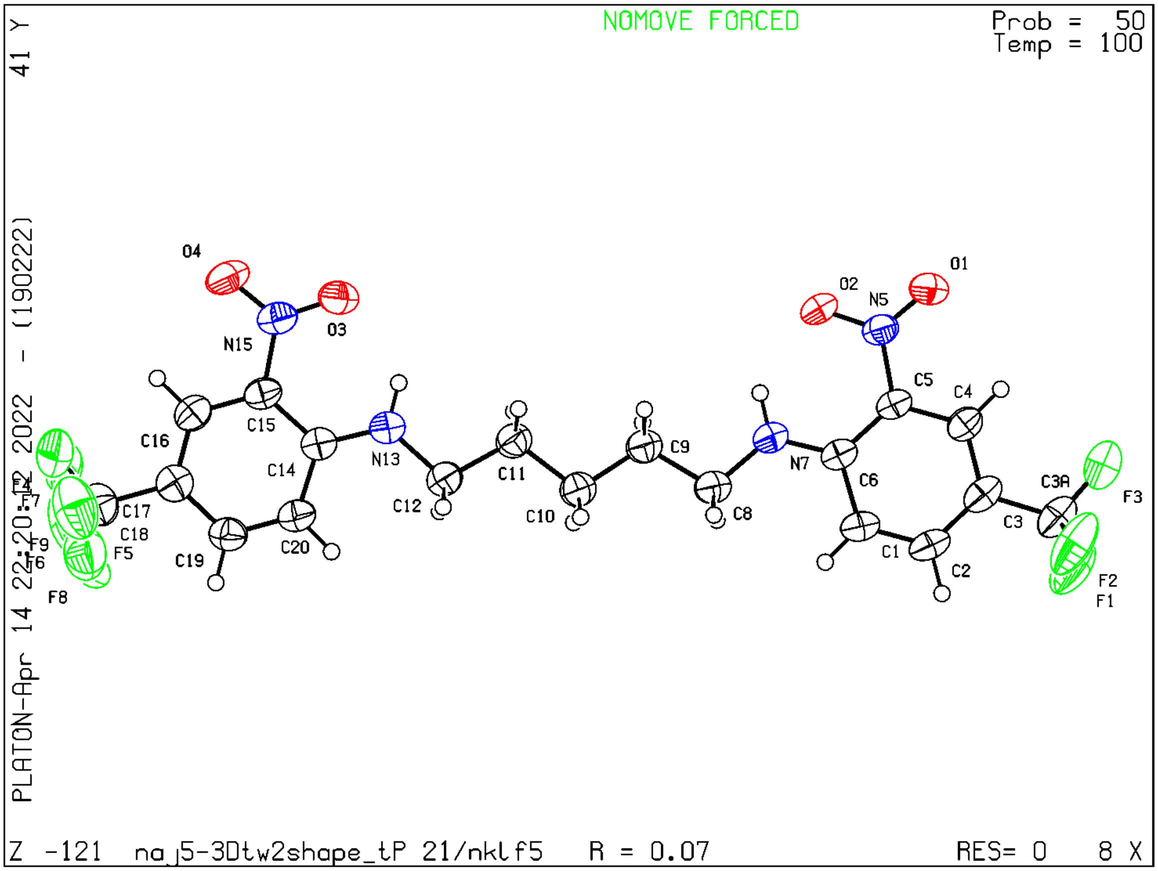

Supplement: Supplementary file 1 [file materials-16-01474-s001.zip › materials-2144164-supplementary/materials-2144164-supplementary/Supplementary + cif/Cad-FNBT-checkcif.pdf]
