# Supplementary material for: The Selection of the Best Derivatization Reagents for the Determination of Polyamines in Home-Made Wine Samples
Source: Materials (Basel). 2023 Feb 9;16(4):1474. doi: 10.3390/ma16041474 (PMC9960030; doi:10.3390/ma16041474)

checkCIF/PLATON report

Structure factors have been supplied for datablock(s) naj6-3Dshape

THIS REPORT IS FOR GUIDANCE ONLY. IF USED AS PART OF A REVIEW PROCEDURE FOR PUBLICATION, IT SHOULD NOT REPLACE THE EXPERTISE OF AN EXPERIENCED CRYSTALLOGRAPHIC REFEREE.

No syntax errors found. CIF dictionary Interpreting this report

Datablock: naj6-3Dshape

Bond precision: C-C = 0.0027 A Wavelength=1.54184

Cell:	a=4.9668(2)	b=6.5809(3)	c=14.7689(6)
	alpha=83.837(3)	beta=85.011(3)	gamma=85.340(3)
Temperature:	100 K		

	Calculated	Reported
Volume	476.87(4)	476.87(4)
Space group	P -1	P -1
Hall group	-P 1	-P 1
Moiety formula	C18 H16 F6 N4 O4	C18 H16 F6 N4 O4
Sum formula	C18 H16 F6 N4 O4	C18 H16 F6 N4 O4
Mr	466.35	466.35
Dx,g cm-3	1.624	1.624
Z	1	1
Mu (mm-1)	1.368	1.368
F000	238.0	238.0
F000’	239.04	
h,k,lmax	6,8,18	5,8,17
Nref	1939	1791
Tmin,Tmax	0.952,0.960	0.888,1.000
Tmin’	0.782	

Correction method= # Reported T Limits: Tmin=0.888 Tmax=1.000
AbsCorr = MULTI-SCAN

Data completeness= 0.924 Theta(max)= 74.322

R(reflections)= 0.0438(1624) wR2(reflections)= 0.1252(1791)

S = 1.061 Npar= 145

The following ALERTS were generated. Each ALERT has the format
test-name_ALERT_alert-type_alert-level.
Click on the hyperlinks for more details of the test.

Alert level C

```
PLAT911_ALERT_3_C Missing FCF Refl Between Thmin & STh/L=    0.600          27 Report
```

Alert level G

```
PLAT007_ALERT_5_G Number of Unrefined Donor-H Atoms .............           1 Report
PLAT154_ALERT_1_G The s.u.'s on the Cell Angles are Equal ..(Note)      0.003 Degree
PLAT242_ALERT_2_G Low    'MainMol' Ueq as Compared to Neighbors of        C3A Check
PLAT883_ALERT_1_G No Info/Value for _atom_sites_solution_primary .     Please Do !
PLAT898_ALERT_4_G Second Reported H-M Symbol in CIF Ignored ......          ! Check
PLAT912_ALERT_4_G Missing # of FCF Reflections Above STh/L=  0.600         84 Note
PLAT933_ALERT_2_G Number of HKL-OMIT Records in Embedded .res File          1 Note
PLAT941_ALERT_3_G Average HKL Measurement Multiplicity ..........         2.6 Low
PLAT965_ALERT_2_G The SHELXL WEIGHT Optimisation has not Converged     Please Check
PLAT978_ALERT_2_G Number C-C Bonds with Positive Residual Density.          6 Info
```

0 **ALERT level A** = Most likely a serious problem - resolve or explain
0 **ALERT level B** = A potentially serious problem, consider carefully
1 **ALERT level C** = Check. Ensure it is not caused by an omission or oversight
10 **ALERT level G** = General information/check it is not something unexpected

2 ALERT type 1 CIF construction/syntax error, inconsistent or missing data
4 ALERT type 2 Indicator that the structure model may be wrong or deficient
2 ALERT type 3 Indicator that the structure quality may be low
2 ALERT type 4 Improvement, methodology, query or suggestion
1 ALERT type 5 Informative message, check

It is advisable to attempt to resolve as many as possible of the alerts in all categories. Often the minor alerts point to easily fixed oversights, errors and omissions in your CIF or refinement strategy, so attention to these fine details can be worthwhile. In order to resolve some of the more serious problems it may be necessary to carry out additional measurements or structure refinements. However, the purpose of your study may justify the reported deviations and the more serious of these should normally be commented upon in the discussion or experimental section of a paper or in the "special_details" fields of the CIF. checkCIF was carefully designed to identify outliers and unusual parameters, but every test has its limitations and alerts that are not important in a particular case may appear. Conversely, the absence of alerts does not guarantee there are no aspects of the results needing attention. It is up to the individual to critically assess their own results and, if necessary, seek expert advice.

Publication of your CIF in IUCr journals

A basic structural check has been run on your CIF. These basic checks will be run on all CIFs submitted for publication in IUCr journals (*Acta Crystallographica*, *Journal of Applied Crystallography*, *Journal of Synchrotron Radiation*); however, if you intend to submit to *Acta Crystallographica Section C* or *E* or *IUCrData*, you should make sure that full publication checks are run on the final version of your CIF prior to submission.

Publication of your CIF in other journals

Please refer to the *Notes for Authors* of the relevant journal for any special instructions relating to CIF submission.

PLATON version of 19/02/2022; check.def file version of 19/02/2022

Datablock naj6-3Dshape - ellipsoid plot

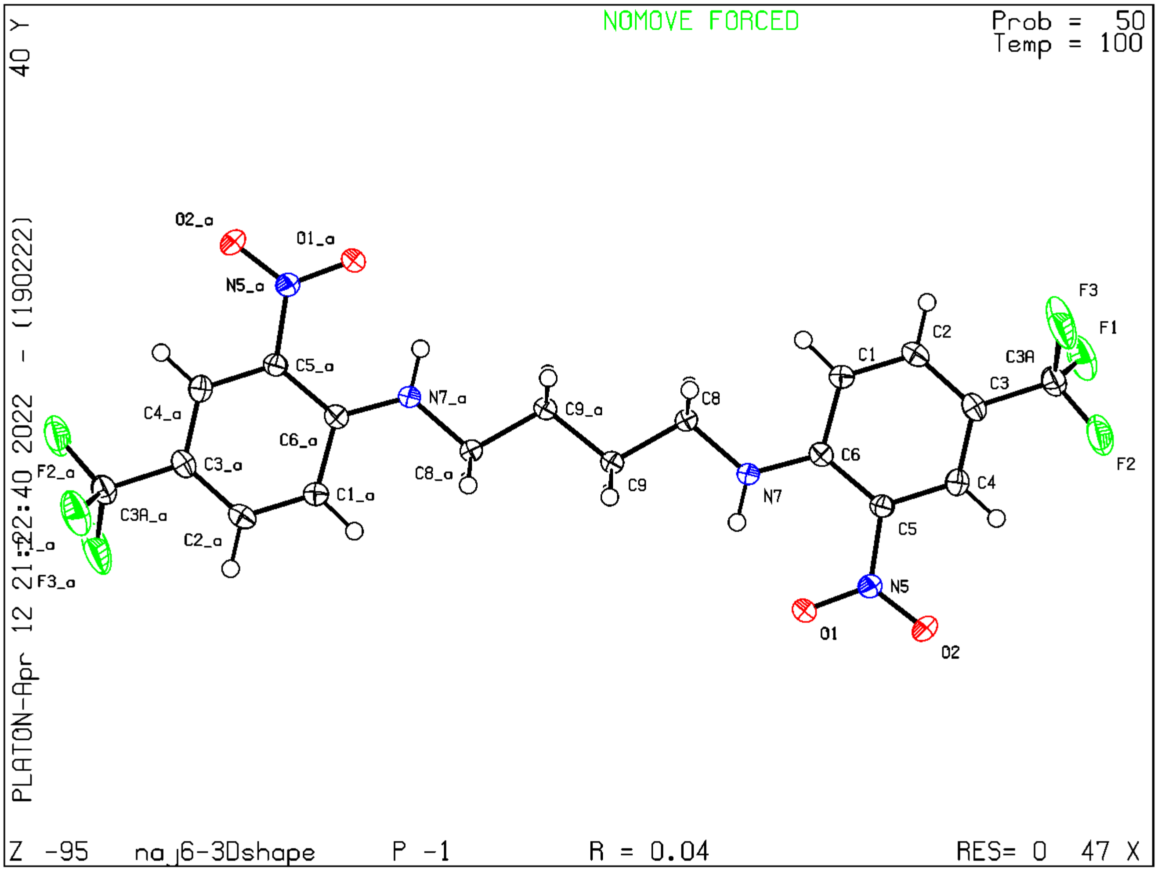

Supplement: Supplementary file 1 [file materials-16-01474-s001.zip › materials-2144164-supplementary/materials-2144164-supplementary/Supplementary + cif/Put-FNBT-checkcif.pdf]
