# Supplementary material for: The Selection of the Best Derivatization Reagents for the Determination of Polyamines in Home-Made Wine Samples
Source: Materials (Basel). 2023 Feb 9;16(4):1474. doi: 10.3390/ma16041474 (PMC9960030; doi:10.3390/ma16041474)
Supplement: Supplementary file 1 [file materials-16-01474-s001.zip › materials-2144164-supplementary/materials-2144164-supplementary/Supplementary + cif/SUPPLEMENTARY MATERIALS after revision - 6.02.2023.pdf]

# The selection of the best derivatization reagents for the determination of polyamines in homemade wine samples

**Anna Kmieciak, Aneta Jastrzębska*, Karolina Szymańska, Marek P. Krzemiński, Tadeusz M. Muzioł, Marzanna Kurzawa, Edward Szłyk**

**Faculty of Chemistry**

**Nicolaus Copernicus University in Toruń**

***aj@umk.pl**

***eszlyk@umk.pl**

# PAs-FNBT

***N^1^,N^4^*-Bis(2-nitro-4-(trifluoromethyl)phenyl)butane-1,4-diamine (Put-FNBT)**

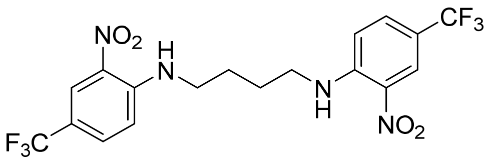


**Yields:** 0.4290 g, 92%

**Melting point** 138-140 °C

**$^1$H NMR** (700 MHz, $CDCl_3$) δ ppm 1.91 – 1.97 (m, 4 H), 3.46 (m, 4 H), 6.93 (d, *J*=9.03 Hz, 2 H), 7.63 (dd, *J*=9.03, 2.15 Hz, 2 H), 8.29 (br. s., 2 H), 8.47 (d, *J*=1.29 Hz, 2 H).

**$^{13}$C NMR** (101 MHz, $CDCl_3$) δ ppm 26.32 (2x$CH_2$), 42.70 (2x$CH_2$), 114.23 (2xCH), 117.72 (q, *J*=34.7 Hz, 2x<u>C</u>-$CF_3$), 123.53 (q, *J*=270.9 Hz, 2x$CF_3$), 125.02 (q, *J*=4.24 Hz, 2xCH), 131.07 (2x<u>C</u>-$NO_2$), 132.26 (q, *J*=3.08 Hz, 2xCH), 146.64 (2x<u>C</u>-NH-).

**$^{19}$F NMR** (376 MHz, $CDCl_3$) δ ppm -62.93 (s, 2x$CF_3$).

**FTIR** (ATR) ($cm^{-1}$): 3361.29, 3094.13, 2956.20, 2922.73, 2872.34, 1631.67, 1572.37, 1529.28, 1472.68, 1430.08, 1411.09, 1363.11, 1316.72, 1250.23, 1218.49, 1182.98, 1115.74, 1076.29, 1061.05, 968.74, 939.92, 915.52, 899.19, 818.21, 765.66, 749.95, 694.56, 642.40, 613.84, 559.19, 516.19, 485.75, 432.26.

***N^1^,N^5^*-Bis(2-nitro-4-(trifluoromethyl)phenyl)pentane-1,5-diamine (Cad-FNBT)**

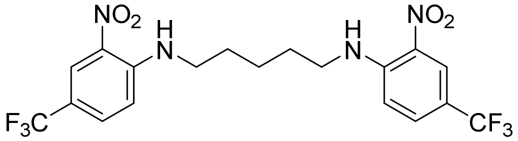


**Yields:** 0.4704 g, 98%

**Melting point** 92-94 °C

**$^1$H NMR** (400 MHz, $CDCl_3$) δ ppm 1.58 – 1.69 (m, 2 H), 1.85 (quin, *J*=7.34 Hz, 4 H), 3.35 – 3.44 (m, 4 H), 6.94 (d, *J*=9.10 Hz, 2 H), 7.63 (dd, *J*=9.10, 2.05 Hz, 2 H), 8.28 (br. s., 2 H), 8.47 (d, *J*=1.17 Hz, 2 H).

**$^{13}$C NMR** (101 MHz, $CDCl_3$) δ ppm 24.44 ($CH_2$), 28.47 (2x$CH_2$), 42.90 (2x$CH_2$), 114.34 (2xCH), 117.38 (q, *J*=34.5 Hz, 2x<u>C</u>-$CF_3$), 123.56 (q, *J*=270.5 Hz, 2x$CF_3$), 124.93 (q, *J*=4.24 Hz, 2xCH), 130.85 (2x<u>C</u>-$NO_2$), 132.15 (q, *J*=3.08 Hz, 2xCH), 146.77 (2x<u>C</u>-NH-).

**$^{19}$F NMR** (376 MHz, $CDCl_3$) δ ppm -62.89 (s, 2x$CF_3$).

**FTIR** (ATR) ($cm^{-1}$): 3380.38, 3361.94, 3101.57, 2933.92, 2870.89, 1634.29, 1574.73, 1525.96, 1473.49, 1434.88, 1413.71, 1362.20, 1321.19, 1296.30, 1266.65, 1250.88, 1225.76, 1188.70, 1150.48, 1129.40, 1101.52, 1079.07, 972.77, 918.73, 910.21, 897.82, 819.49, 764.36, 732.74, 690.58, 642.87, 617.41, 580.19, 516.51, 486.46, 453.85, 430.09.

***N$^1$*,*N$^4$*-Bis(2-nitro-4-(trifluoromethyl)phenyl)-*N$^1$*-(3-((2-nitro-4-(trifluoromethyl)phenyl)-amino)propyl)butane-1,4-diamine (Spd-FNBT)**

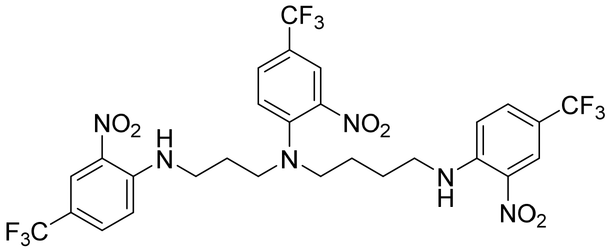


**Yields:** 0.6835 g, 96%

**Melting point** 210-215 °C

**$^1$H NMR** (400 MHz, Acetonitrile-*$d_3$*) δ ppm 1.59 - 1.70 (m, 4 H), 1.89 - 1.98 (m, 2 H), 3.23 - 3.41 (m, 8 H), 6.94 (d, *J*=9.10 Hz, 1 H), 7.01 (d, *J*=9.10 Hz, 1 H), 7.29 (d, *J*=8.80 Hz, 1 H), 7.53 - 7.63 (m, 3 H), 7.90 (dd, *J*=2.20, 0.73 Hz, 1 H), 8.13 (t, *J*=5.43 Hz, 1 H), 8.20 (t, *J*=5.28 Hz, 1 H), 8.31 (dt, *J*=1.98, 0.92 Hz, 2 H).

**$^{13}$C NMR** (101 MHz, Acetonitrile-*$d_3$*) δ ppm 25.65 ($CH_2$), 26.71 ($CH_2$), 27.34 ($CH_2$), 41.20 ($CH_2$), 43.66 ($CH_2$), 50.02 ($CH_2$), 52.49 ($CH_2$), 116.55 (CH), 116.77 (CH), 117.25 (q, *J*=34.3 Hz, C-$CF_3$), 117.40 (q, *J*=33.9 Hz, C-$CF_3$), 121.03 (q, *J*=33.9 Hz, C-$CF_3$), 123.39 (CH), 125.09 (q, *J*=270.5 Hz, $CF_3$), 125.28 (q, *J*=3.85 Hz, CH),125.36 (q, *J*=269.7 Hz, $CF_3$), 125.40 (q, *J*=269.7 Hz, $CF_3$), 125.78 (q, *J*=4.24 Hz, CH), 125.80 (q, *J*=4.24 Hz, CH), 130.67 (q, *J*=3.47 Hz, CH), 132.06 (C-$NO_2$), 132.21 (C-$NO_2$), 133.06 (q, *J*=3.08 Hz, 2xCH), 141.66 (C-$NO_2$), 148.23 (C-N), 148.31 (C-NH-), 148.42 (C-NH-).

**$^{19}$F NMR** (376 MHz, Acetonitrile-*$d_3$*) δ ppm -63.35 (s, 2x$CF_3$), -63.32 (s, $CF_3$).

**$^{19}$F NMR** (376 MHz, THF-*$d_8$*) δ ppm -63.64 (s, $CF_3$), -63.60 (s, $CF_3$), -63.57 (s, $CF_3$).

**FTIR** (ATR) ($cm^{-1}$): 3372.04, 3327.38, 3112.24, 2942.34, 2858.91, 1639.20, 1572.56, 1536.29, 1466.59, 1434.49, 1414.60, 1385.34, 1364.13, 1322.24, 1260.51, 1231.31, 1185.06, 1155.11, 1107.27, 1079.47, 901.71, 872.74, 819.99, 762.10, 714.97, 692.98, 645.25, 615.92, 541.15, 515.27, 432.98, 417.90.

***$N^1$,$N^{1'}$*-(Butane-1,4-diyl)bis(*$N^1$,$N^3$*-bis(2-nitro-4-(trifluoromethyl)phenyl)propane-1,3-diamine) (Spm-FNBT)**

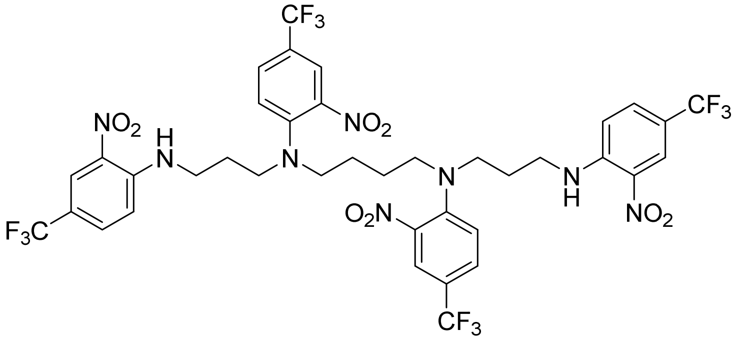

**Yields:** 0.9101 g, 95%

**Melting point** 135-138 °C

**$^1$H NMR** (400 MHz, $CDCl_3$) δ ppm 1.50 (m, 4 H), 1.95 (quin, *J*=6.75 Hz, 4 H), 3.11 (m, 4 H), 3.31 (t, *J*=6.60 Hz, 4 H), 3.34 – 3.42 (m, 4 H), 6.87 (d, *J*=8.80 Hz, 2 H), 7.17 (d, *J*=8.51 Hz, 2 H), 7.55 – 7.63 (m, 4 H), 7.92 (d, *J*=1.76 Hz, 2 H), 8.18 (br. s., 2 H), 8.43 (d, *J*=1.17 Hz, 2 H).

**$^{13}$C NMR** (101 MHz, $CDCl_3$) δ ppm 24.47 (2x$CH_2$), 26.44 (2x$CH_2$), 40.04 (2x$CH_2$), 48.65 (2x$CH_2$), 52.57 (2x$CH_2$), 114.25 (2xCH), 117.75 (q, *J*=34.3 Hz, 2x$\underline{C}$-$CF_3$), 121.86 (2xCH), 122.39 (q, *J*=34.7 Hz, 2x$\underline{C}$-$CF_3$), 123.09 (q, *J*=271.7 Hz, 2x$CF_3$), 123.50 (q, *J*=270.9 Hz, 2x$CF_3$), 123.89 (q, *J*=3.85 Hz, 2xCH), 124.96 (q, *J*=4.24 Hz, 2xCH), 129.55 (q, *J*=3.08 Hz, 2xCH), 130.85 (2x$\underline{C}$-$NO_2$), 132.25 (q, *J*=3.08 Hz, 2xCH), 141.67 (2x$\underline{C}$-$NO_2$), 146.18 (2x$\underline{C}$-N), 146.60 (2x$\underline{C}$-NH-).

**$^{19}$F NMR** (376 MHz, $CDCl_3$) δ ppm -62.97 (s, 2x$CF_3$), -63.11 (s, 2x$CF_3$).

**FTIR** (ATR) ($cm^{-1}$): 3378.55, 3112.64, 2940.92, 2879.32, 1737.77, 1637.83, 1624.32, 1578.28, 1561.10, 1528.88, 1504.03, 1434.93, 1398.70, 1374.89, 1359.85, 1320.33, 1281.02, 1263.55, 1235.35, 1205.34, 1194.68, 1155.46, 1126.91, 1079.87, 1044.47, 974.57, 920.86, 905.13, 866.33, 828.08, 818.06, 773.97, 763.14, 753.93, 714.96, 689.30, 670.46, 637.59, 607.08, 569.50, 518.77, 474.95.

# PAs-CNBF

***$N^1$,$N^4$*-Bis(2,6-dinitro-4-(trifluoromethyl)phenyl)butane-1,4-diamine (Put-CNBF)**

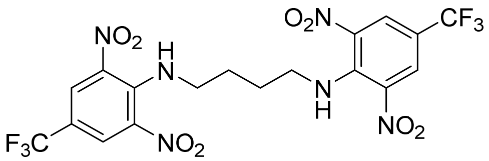

**Yields:** 0.5230 g, 94%

**Melting point** 190-194 °C

**$^{1}$H NMR** (400 MHz, Acetone-*d6*) δ ppm 1.89 (dt, *J*=6.09, 3.26 Hz, 4 H), 3.12 – 3.20 (m, 4 H), 8.52 (s, 4 H), 8.58 (br. s., 2 H).

**$^{13}$C NMR** (101 MHz, Acetone-*d6*) δ ppm 27.73 (2x$CH_2$), 46.98 (2x$CH_2$), 116.06 (q, *J*=35.8 Hz, 2x$\underline{C}$-$CF_3$), 123.96 (q, *J*=270.9 Hz, 2x$CF_3$), 129.76 (q, *J*=3.85 Hz, 4xCH), 138.82 (4x$\underline{C}$-$NO_2$), 142.43 (2x$\underline{C}$-NH-).

**$^{19}$F NMR** (376 MHz, Acetone-*d6*) δ ppm -63.44 (s, 2x$CF_3$).

**FTIR** (ATR) ($cm^{-1}$): 3351.23, 3102.59, 3062.69, 2955.16, 2929.02, 2874.92, 2115.20, 1861.68, 1738.39, 1635.35, 1577.00, 1537.86, 1516.18, 1449.40, 1409.83, 1370.48, 1357.31, 1279.51, 1249.25, 1166.62, 1125.66, 1065.49, 1005.11, 963.70, 944.44, 919.59, 899.35, 832.37, 787.08, 766.69, 759.17, 737.42, 723.81, 706.58, 670.13, 653.76, 642.41, 588.68, 531.37, 503.67, 452.49, 425.79.

***$N^1$,$N^5$*-Bis(2,6-dinitro-4-(trifluoromethyl)phenyl)pentane-1,5-diamine (Cad-CNBF)**

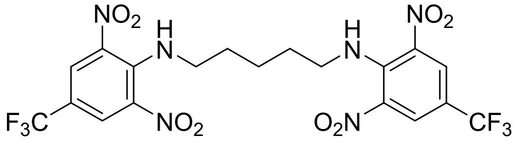


**Yields:** 0.5462 g, 96%

**Melting point** 134-136 °C

**$^{1}$H NMR** (400 MHz, Acetone-*d6*) δ ppm 1.47 – 1.57 (m, 2 H), 1.82 (dt, *J*=14.82, 7.26 Hz, 4 H), 3.07 – 3.15 (m, 4 H), 8.52 (s, 4 H), 8.58 (br. s., 2 H).

**$^{13}$C NMR** (101 MHz, Acetone-*d6*) δ ppm 24.40 ($CH_2$), 30,03 (2x$CH_2$), 47.21 (2x$CH_2$), 115.89 (q, *J*=35.6 Hz, 2x$\underline{C}$-$CF_3$), 123.95 (q, *J*=270.5 Hz, 2x$CF_3$), 129.77 (q, *J*=3.90 Hz, 4xCH), 138.71 (4x$\underline{C}$-$NO_2$), 142.50 (2x$\underline{C}$-NH-).

**$^{19}$F NMR** (376 MHz, Acetone-*d6*) δ ppm -63.41 (s, 2x$CF_3$).

**FTIR** (ATR) ($cm^{-1}$): 3340.55, 3064.59, 2940.78, 1850.99, 1738.46, 1636.56, 1573.50, 1541.42, 1514.09, 1437.54, 1408.88, 1363.96, 1271.30, 1226.04, 1202.60, 1167.78, 1124.59, 954.64, 935.85, 914.95, 896.40, 781.86, 765.20, 727.19, 707.57, 643.01, 535.80, 513.37, 478.20, 451.69.

***$N^1$,$N^4$*-Bis(2,6-dinitro-4-(trifluoromethyl)phenyl)-*$N^1$*-(3-((2,6-dinitro-4-(trifluoromethyl)-phenyl)amino)propyl)butane-1,4-diamine (Spd-CNBF)**

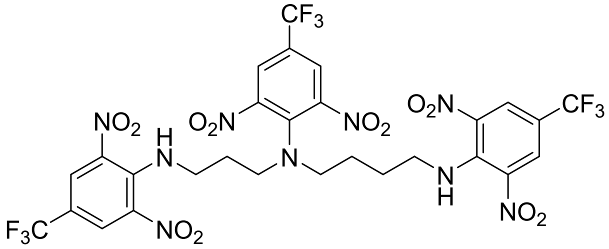


**Yields**: 0.8032 g, 95%

**Melting point** 164-167 °C

[1]**H NMR** (400 MHz, Acetone-*d6*) δ ppm 1.68 - 1.82 (m, 4 H), 2.09 - 2.17 (m, 2 H), 3.06 - 3.19 (m, 6 H), 3.22 (t, *J*=6.75 Hz, 2 H), 8.31 (d, *J*=0.59 Hz, 2 H), 8.46 (m, 3 H, 2xCH, NH), 8.49 - 8.55 (m, 3 H, 2xCH, NH).

[13]**C NMR** (101 MHz, Acetone-*d6*) δ ppm 25.42 ($CH_2$), 27.62 ($CH_2$), 28.57 ($CH_2$), 45.10 ($CH_2$), 47.03 ($CH_2$), 49.61 ($CH_2$), 52.08 ($CH_2$), 116.02 (q, *J*=35.8 Hz, C-$CF_3$), 116.12 (q, *J*=35.5 Hz, C-$CF_3$), 123.25 (q, *J*=35.8 Hz, C-$CF_3$), 123.50 (q, *J*=271.6 Hz, $CF_3$), 123.86 (q, *J*=270.5 Hz, $CF_3$), 123.93 (q, *J*=270.9 Hz, $CF_3$), 127.89 (q, *J*=3.08 Hz, 2xCH), 129.75 (q, *J*=3.47 Hz, 2xCH), 129.79 (q, *J*=3.85 Hz, 2xCH), 138.69 (2xC-$NO_2$), 138.73 (2xC-$NO_2$), 141.62 (2xC-$NO_2$), 142.40 (C-NH-), 142.42 (C-NH-), 146.80 (C-N).

[19]**F NMR** (376 MHz, Acetone-*d6*) δ ppm -63.81 (s, $CF_3$), -63.49 (s, $CF_3$), -63.45 (s, $CF_3$).

**FTIR** (ATR) ($cm^{-1}$): 3341.68, 3077.99, 2969.85, 2929.71, 2324.19, 2113.34, 2014.65, 1996.40, 1738.71, 1637.71, 1578.90, 1523.81, 1474.11, 1448.46, 1433.47, 1411.40, 1365.44, 1303.60, 1278.15, 1228.93, 1181.74, 1129.58, 1105.84, 996.21, 948.22, 910.32, 900.51, 877.51, 793.51, 783.71, 765.22, 740.45, 724.25, 711.31, 660.81, 641.79, 582.55, 515.14, 451.54, 431.22.

***$N^1$,$N^{1'}$*-(Butane-1,4-diyl)bis(*$N^1$,$N^3$*-bis(2,6-dinitro-4-(trifluoromethyl)phenyl)propane-1,3-diamine) (Spm-CNBF)**

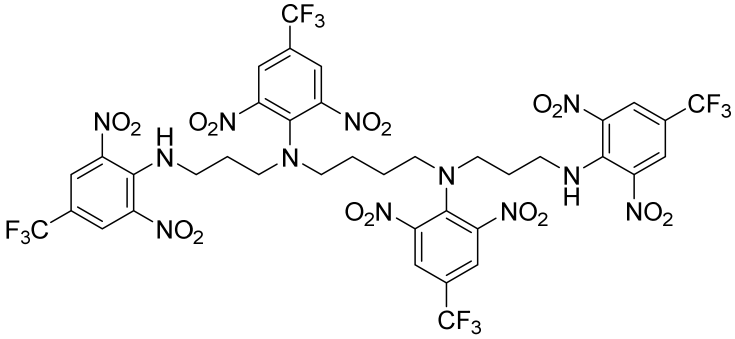


**Yields**: 1.0565 g, 93%

**Melting point** 205-208 °C

**$^1$H NMR** (400 MHz, Acetone-*d6*) δ ppm 1.57 – 1.67 (m, 4 H), 2.11 (quin, *J*=6.68 Hz, 4 H), 3.01 – 3.08 (m, 4 H), 3.11 – 3.22 (m, 8 H), 8.27 (d, *J*=0.59 Hz, 4 H), 8.45 (s, 6 H).

**$^{13}$C NMR** (101 MHz, THF-*d8*) δ ppm 25.65 (2x$CH_2$), 28.86 (2x$CH_2$), 45.10 (2x$CH_2$) 49.78 (2x$CH_2$), 52.44 (2x$CH_2$), 116.54 (q, *J*=35.8 Hz, 2x$\underline{C}$-$CF_3$), 123.69 (q, *J*=272.0 Hz, 2x$CF_3$), 123.98 (q, *J*=35.8 Hz, 2x$\underline{C}$-$CF_3$), 124.10 (q, *J*=270.9 Hz, 2x$CF_3$), 127.82 (q, *J*=3.47 Hz, 4xCH), 129.75 (q, *J*=3.85 Hz, 4xCH), 138.88 (4x$\underline{C}$-$NO_2$), 141.56 (4x$\underline{C}$-$NO_2$), 142.28 (2x$\underline{C}$-NH-), 147.28 (2x$\underline{C}$-NH-).

**$^{19}$F NMR** (376 MHz, Acetone-*d6*) δ ppm -63.80 (s, 2x$CF_3$), -63.50 (s, 2x$CF_3$).

**$^{19}$F NMR** (376 MHz, THF-*d8*) δ ppm -64.06 (s, 2x$CF_3$) -63.71 (s, 2x$CF_3$).

**FTIR** (ATR) ($cm^{-1}$): 3313.20, 3089.31, 2969.78, 2947.66, 2110.38, 2014.74, 1738.55, 1636.82, 1580.92, 1536.65, 1464.43, 1434.75, 1414.18, 1366.13, 1351.30, 1314.11, 1279.28, 1230.02, 1217.44, 1192.93, 1167.23, 1121.32, 1021.37, 953.00, 919.04, 897.32, 819.36, 791.31, 765.25, 730.14, 710.47, 669.53, 659.70, 611.38, 539.92, 520.44, 488.19, 453.90.

# PAs-BPI

**1,1'-(Butane-1,4-diyl)bis(3-(3,5-bis(trifluoromethyl)phenyl)urea) (Put-BPI)**

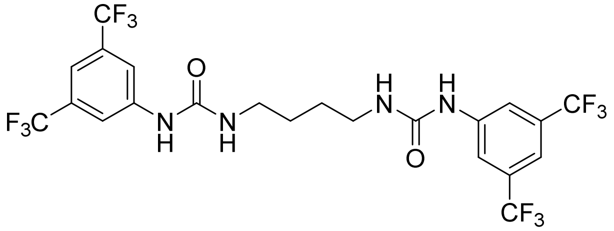


**Yields:** 0.1346 g, 90%

**Melting point** 229-231 °C

**$^1$H NMR** (400 MHz, DMSO-*d6*) δ ppm 1.40 – 1.54 (m, 4 H), 3.05 – 3.19 (m, 4 H), 6.51 (t, *J*=5.72 Hz, 2 H), 7.52 (s, 2 H), 8.07 (s, 4 H), 9.15 – 9.24 (m, 2 H).

**$^{13}$C NMR** (101 MHz, DMSO-*d6*) δ ppm 27.02 (2x$CH_2$), 38.96 (2x$CH_2$), 113.28 (m, 2xCH), 117.14 (m, 4xCH), 123.36 (q, *J*=272.4 Hz, 4x$CF_3$), 130.55 (q, *J*=32.4 Hz, 4x$\underline{C}$-$CF_3$), 142.64 (2x$\underline{C}$-NH-), 155.87 (2xC=O).

**$^{19}$F NMR** (376 MHz, DMSO-*d6*) δ ppm -62.69 (4x$CF_3$).

**FTIR** (ATR) ($cm^{-1}$): 3338.27, 3115.77, 2942.46, 1654.09, 1561.43, 1473.54, 1439.27, 1386.76, 1272.33, 1257.13, 1172.79, 1126.44, 1095.26, 1029.32, 999.67, 940.85, 911.24, 877.52, 846.36, 763.74, 700.05, 679.19, 656.85, 625.46.

**1,1'-(Pentane-1,5-diyl)bis(3-(3,5-bis(trifluoromethyl)phenyl)urea) (Cad-BPI)**

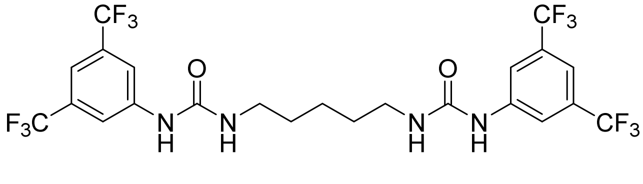


**Yields:** 0.1481 g, 92%

**Melting point** 218-221 °C

**$^{1}$H NMR** (400 MHz, Acetone-*d6*) δ ppm 1.37 - 1.47 (m, 2 H), 1.58 (quin, *J*=7.26 Hz, 4 H), 3.25 (q, *J*=6.75 Hz, 4 H), 6.13 (t, *J*=4.84 Hz, 2 H), 7.50 (s, 2 H), 8.14 (s, 4 H), 8.54 (s, 2 H).

**$^{13}$C NMR** (101 MHz, Acetone-*d6*) δ ppm 24.88 ($CH_2$), 30.57 (2x$CH_2$), 40.52 (2x$CH_2$), 114.73 (sept, *J*=3.85 Hz, 2xCH), 118.49 (q, *J*=3.85 Hz, 4xCH), 124.61 (q, *J*=271.7 Hz, 4x$CF_3$), 132.45 (q, *J*=32.8 Hz, 4x<u>C</u>-$CF_3$), 143.80 (2x<u>C</u>-NH-), 155.84 (2xC=O).

**$^{19}$F NMR** (376 MHz, Acetone-*d6*) δ ppm -64.63 (s, 4x$CF_3$).

**FTIR** (ATR) ($cm^{-1}$): 3327.81, 3119.05, 2937.36, 2863.96, 1655.49, 1569.13, 1473.96, 1439.54, 1387.70, 1271.38, 1171.11, 1126.91, 1039.30, 1015.93, 936,12, 913.67, 875.77, 849.66, 699.46, 679.39, 663.31, 641.16.

**3-(3,5-Bis(trifluoromethyl)phenyl)-1-(4-(3-(3,5-bis(trifluoromethyl)phenyl)ureido)butyl)-1-(3-(3-(3,5-bis(trifluoromethyl)phenyl)ureido)propyl)urea (Spd-BPI)**

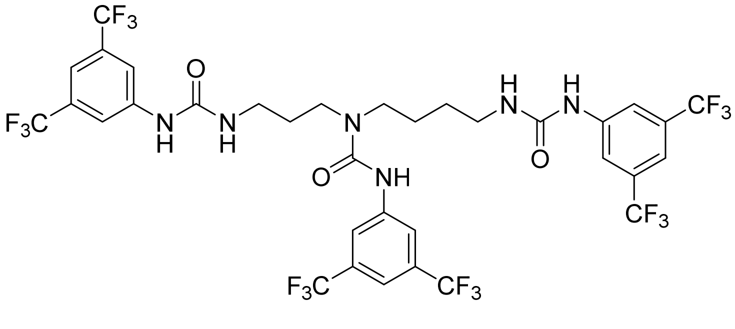


**Yields:** 0.1546 g, 90%

**Melting point** 120-124 °C

**$^{1}$H NMR** (400 MHz, DMSO-*d6*) δ ppm 1.41 – 1.52 (m, 2 H), 1.52 – 1.62 (m, 2 H), 1.73 (quin, *J*=6.60 Hz, 2 H), 3.09 – 3.19 (m, 4 H), 3.35 – 3.44 (m, 4 H), 6.47 – 6.57 (m, 2 H), 7.49 (s, 2 H), 7.52 (s, 1 H), 8.05 (s, 4 H), 8.24 (s, 2 H), 8.86 (s, 1 H), 9.20 (s, 1 H), 9.30 (s, 1 H).

**$^{13}$C NMR** (101 MHz, DMSO-*d6*) δ ppm 25.34 ($CH_2$), 26.96 ($CH_2$), 28.43 ($CH_2$), 36.78 ($CH_2$), 38.89 ($CH_2$), 43.96 ($CH_2$), 46.04 ($CH_2$), 113.28 (m, 2xCH), 113.89 (m, CH), 117.10 (m, 4xCH), 118.98 (m, 2xCH), 123.33 (q, *J*=272.8 Hz, 4x$CF_3$), 123.39 (q, *J*=272.4 Hz, 2x$CF_3$), 130.19 (q, *J*=32.4 Hz, 2x$\underline{C}$-$CF_3$), 130.54 (q, *J*=32.4 Hz, 4x$\underline{C}$-$CF_3$), 142.58 (2x$\underline{C}$-NH-), 142.71 ($\underline{C}$-NH-), 154.43 (C=O), 154.80 (C=O), 154.83 (C=O).

**$^{19}$F NMR** (376 MHz, DMSO-*d6*) δ ppm -62.81, -62.80 (br. s, 4x$CF_3$), -62.68 (s, 2x$CF_3$).

**FTIR** (ATR) ($cm^{-1}$): 3306.86, 3101.65, 2944.16, 1640.40, 1561.69, 1472.48, 1441.16, 1384, 89, 1273.60, 1168.83, 1119.82, 1001.65, 941.47, 879.98, 844.89, 756.45, 731.50, 701.03, 680.66, 627.34.

**1,1'-((Butane-1,4-diylbis(((3,5-bis(trifluoromethyl)phenyl)carbamoyl)azanediyl))-bis(propane-3,1-diyl))bis(3-(3,5-bis(trifluoromethyl)phenyl)urea) (Spm-BPI)**

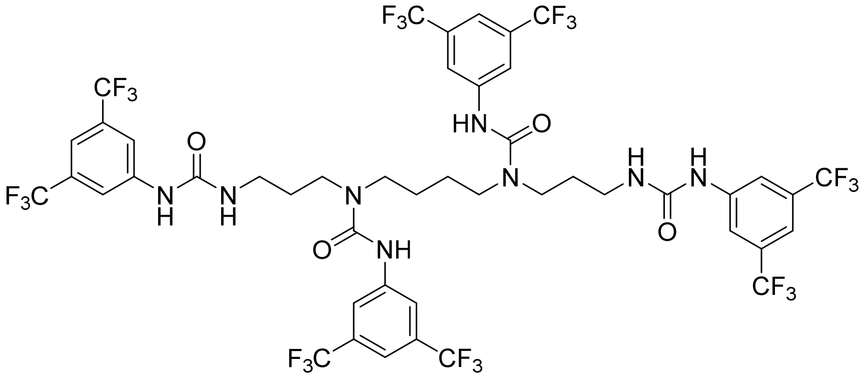

**Yields:** 0.1730 g, 93%

**Melting point** 125-130 °C

**$^{1}$H NMR** (700 MHz, DMSO-*d6*) δ ppm 1.56 (m, 4 H), 1.71 (m, 4 H), 3.13 (q, *J*=6.53 Hz, 4 H), 3.36 – 3.40 (m, 8 H), 6.49 (t, *J*=5.70 Hz, 2 H), 7.50 (s, 2 H), 7.53 (s, 2 H), 8.04 (s, 4 H), 8.21 (s, 4 H), 8.85 (s, 2 H), 9.28 (br. s., 2 H).

**$^{1}$H NMR** (400 MHz, Acetone-*d6*) δ ppm 1.71 – 1.80 (m, 4 H), 1.89 (quin, *J*=6.68 Hz, 4 H), 3.25 – 3.35 (m, 4 H), 3.49 – 3.61 (m, 8 H), 7.51 (s, 4 H), 8.13 (s, 4 H), 8.20 (s, 4 H), 8.60 (s, 1 H), 8.78 (br. s., 1 H).

**$^{13}$C NMR** (101 MHz, DMSO-*d6*) δ ppm 25.30 (2x$CH_2$), 28.55 (2x$CH_2$), 36.82 (2x$CH_2$), 44.05 (2x$CH_2$), 46.14 (2x$CH_2$), 113.19 (m, 2xCH), 113.88 (m, 2xCH), 117.15 (m, 4xCH), 119.10 (m, 4xCH), 123.38 (q, *J*=272.4 Hz, 4x$CF_3$), 123.43 (q, *J*=272.8 Hz, 4x$CF_3$), 130.31 (q, *J*=32.4 Hz, 4x$\underline{C}$-$CF_3$), 130.65 (q, *J*=32.4 Hz, 4x$\underline{C}$-$CF_3$), 142.66 (2x$\underline{C}$-NH-), 142.76 (2x$\underline{C}$-NH-), 154.55 (2xC=O), 154.90 (2xC=O).

$^{13}$**C NMR** (101 MHz, Acetone-*d6*) δ ppm 26.24 (2x$CH_2$), 29.73 (2x$CH_2$), 37.99 (2x$CH_2$), 45.15 (2x$CH_2$), 47.21 (2x$CH_2$), 114.86 (m, 2xCH), 115.51 (m, 2xCH), 118.59 (m, 4xCH), 120.49 (m, 4xCH), 124.58 (q, *J*=272.0 Hz, 8x$CF_3$), 132.16 (q, *J*=32.8 Hz, 4x<u>C</u>-$CF_3$), 132.47 (q, *J*=32.8 Hz, 4x<u>C</u>-$CF_3$), 143.63 (2x<u>C</u>-NH-), 143.65 (2x<u>C</u>-NH-), 156.14 (2xC=O), 156.21 (2xC=O).

$^{19}$**F NMR** (376 MHz, DMSO-*d6*) δ ppm -62.78 (s, 4x$CF_3$), -62.69 (s, 4x$CF_3$).

$^{19}$**F NMR** (376 MHz, Acetone-*d6*) δ ppm -64.57 (s, 4x$CF_3$), -64.65 (s, 4x$CF_3$).

**FTIR** (ATR) ($cm^{-1}$): 3311.12, 3115.62, 2942.14, 1640.51, 1539.43, 1472.32, 1442.34, 1374.70, 1273.73, 1168.66, 1119.52, 1001.62, 941.52, 880.32, 842.29, 756.51, 732.03, 700.90, 680.88, 629.06.

**Put-FNBT** ($^1$H NMR)

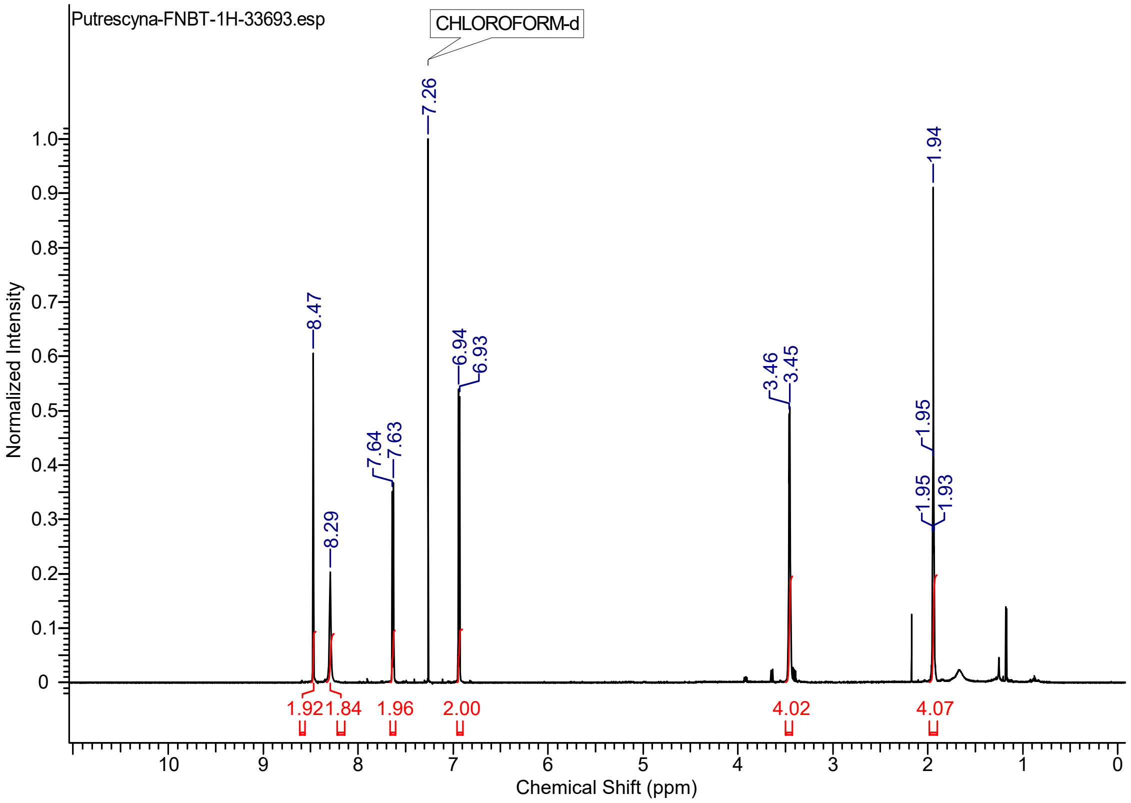


**Put-FNBT** ($^{19}$F NMR)

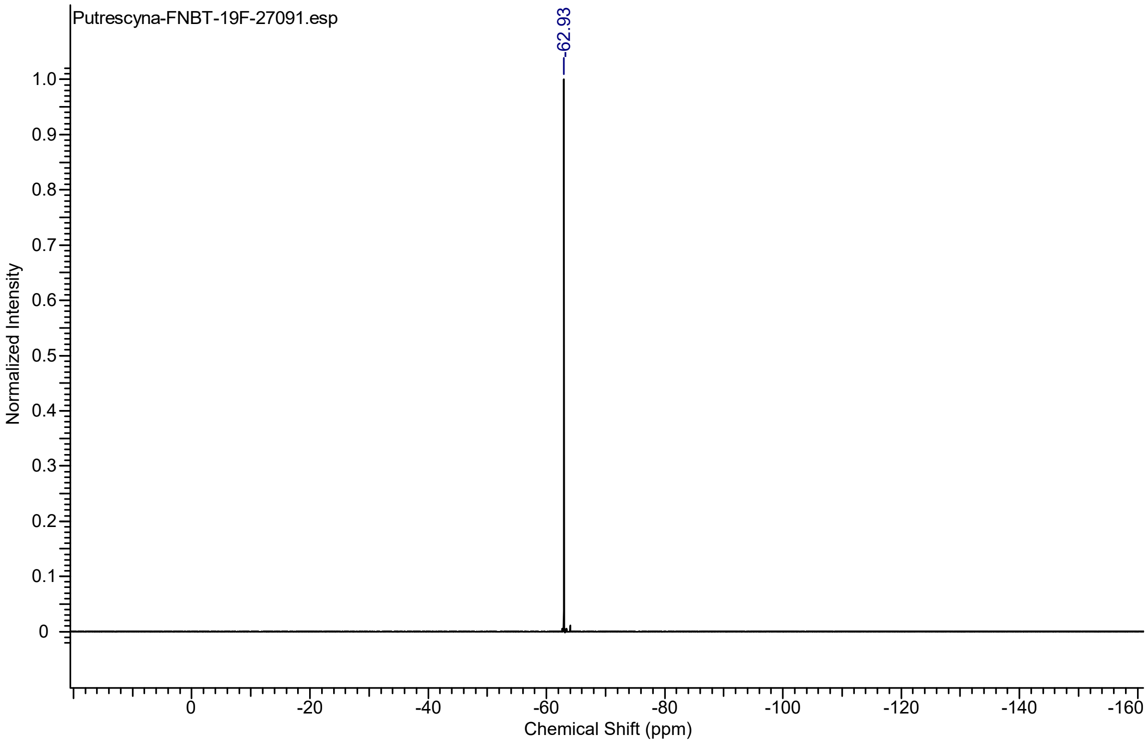

**Put-FNBT** ($^{13}C$ NMR)

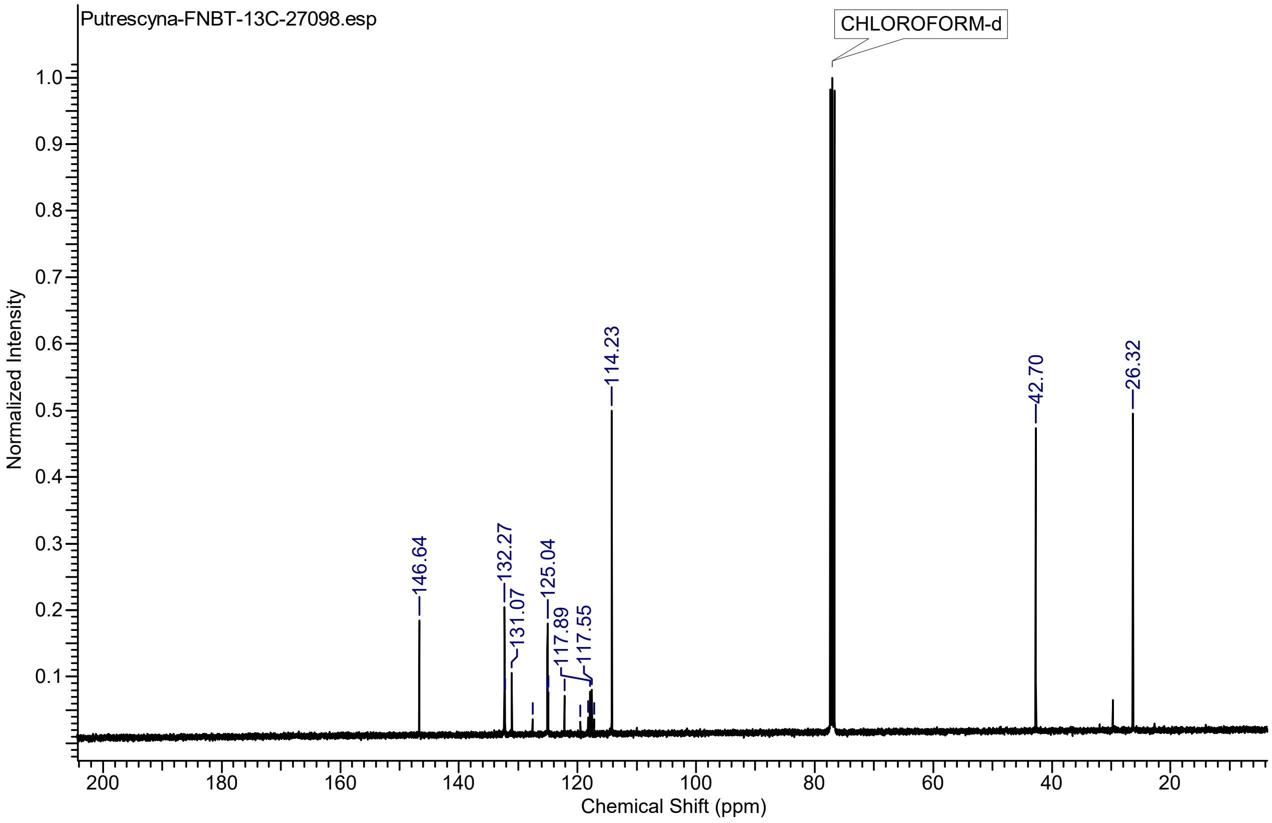


**Cad-FNBT** ($^{1}H$ NMR)

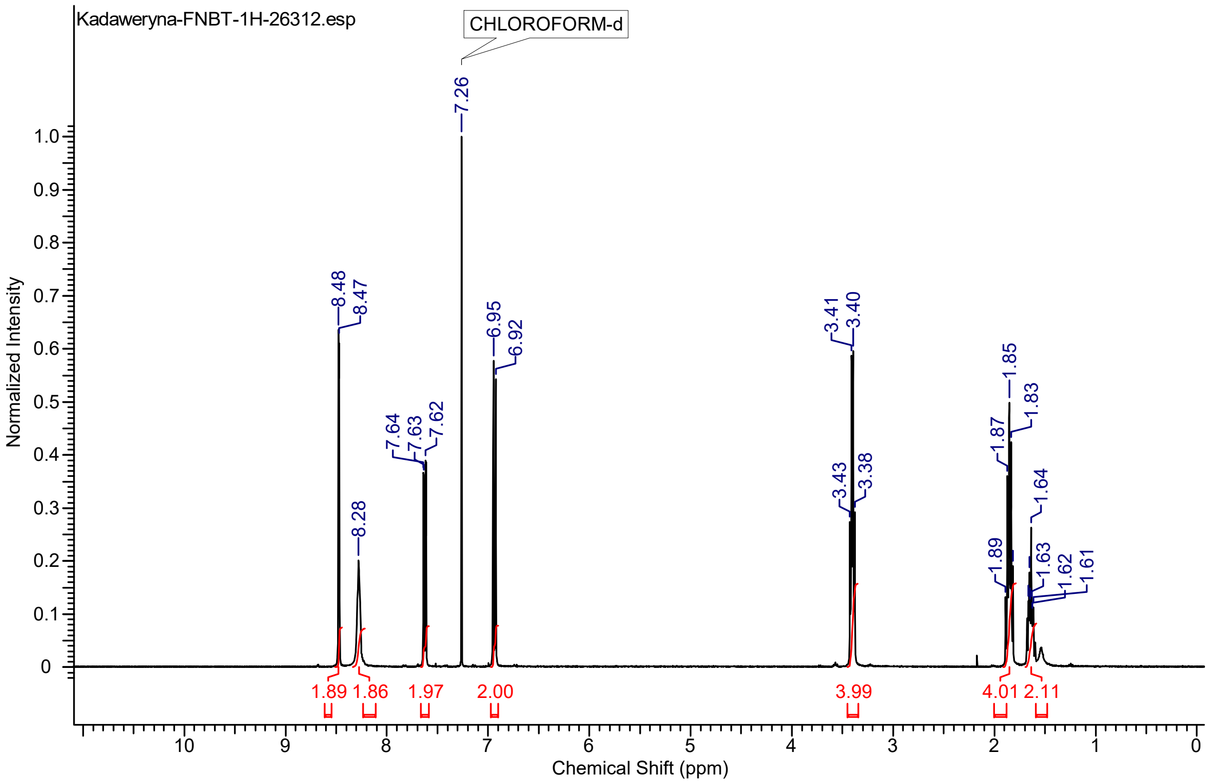

**Cad-FNBT** ($^{19}$F NMR)

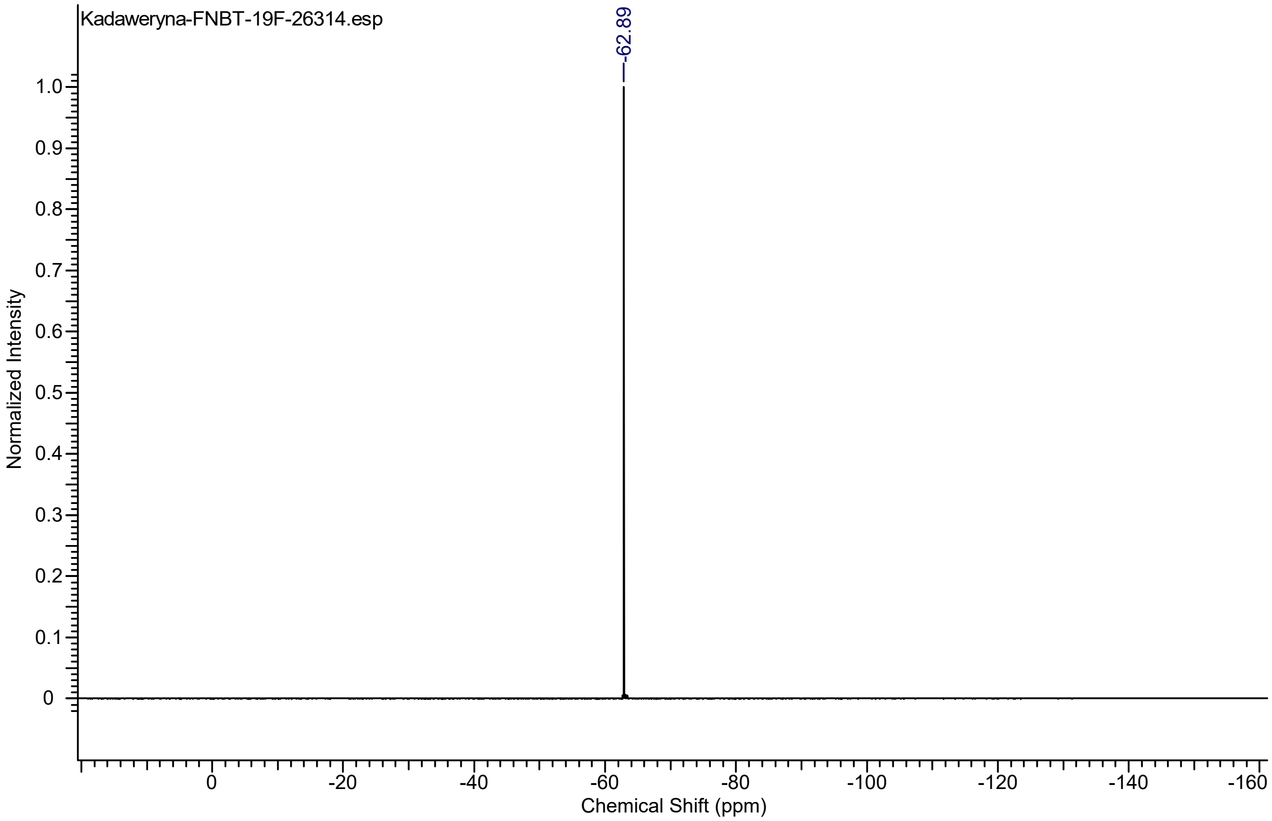


**Cad-FNBT** ($^{13}$C NMR)

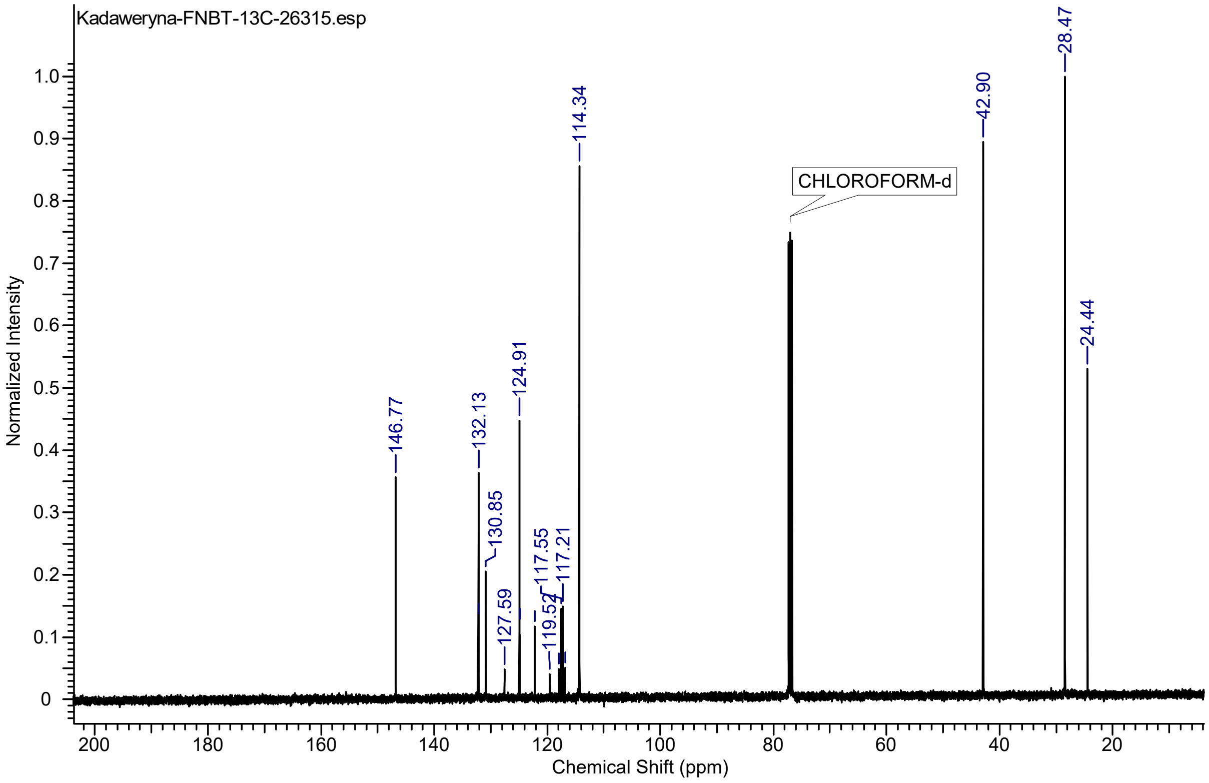

**Spd-CNBF** ($^{1}$H NMR)

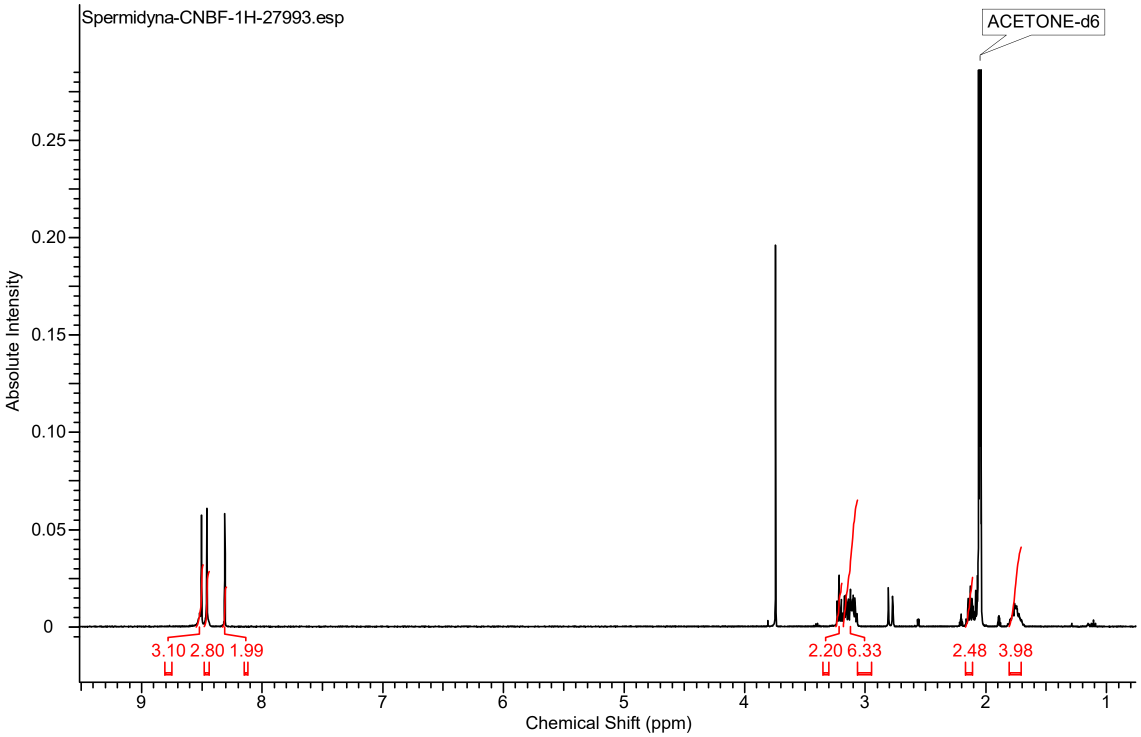


**Spd-CNBF** ($^{19}$F NMR)

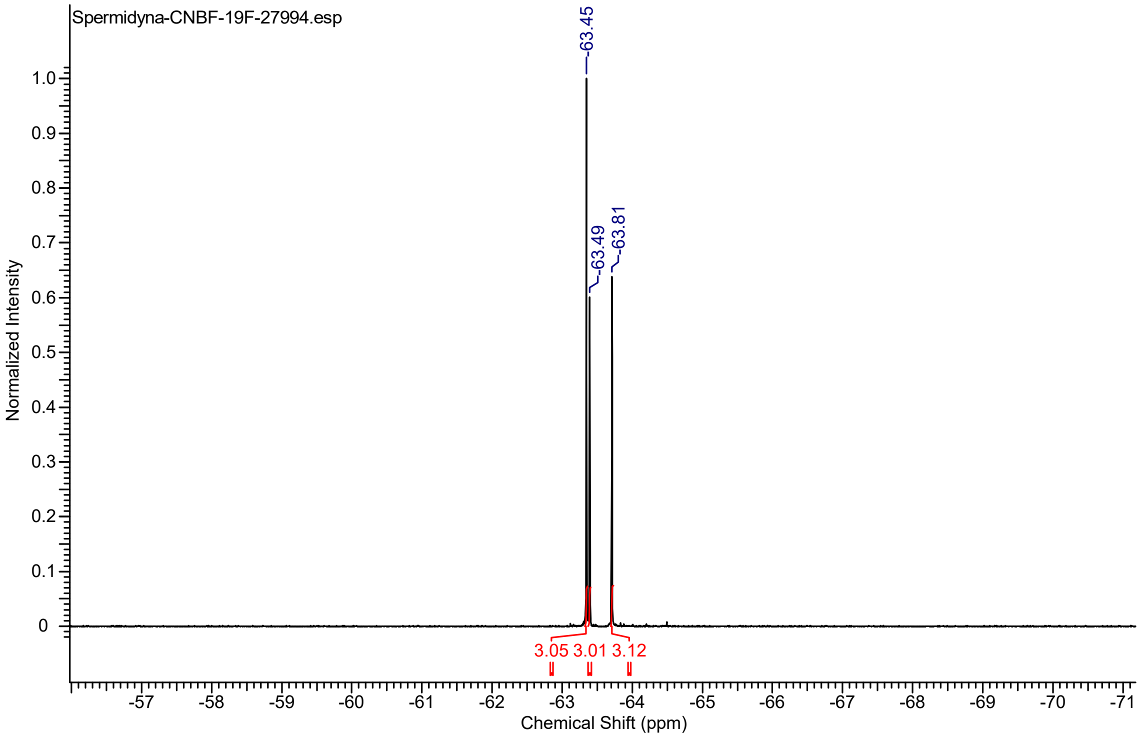

**Spd-CNBF** ($^{1}$H NMR)

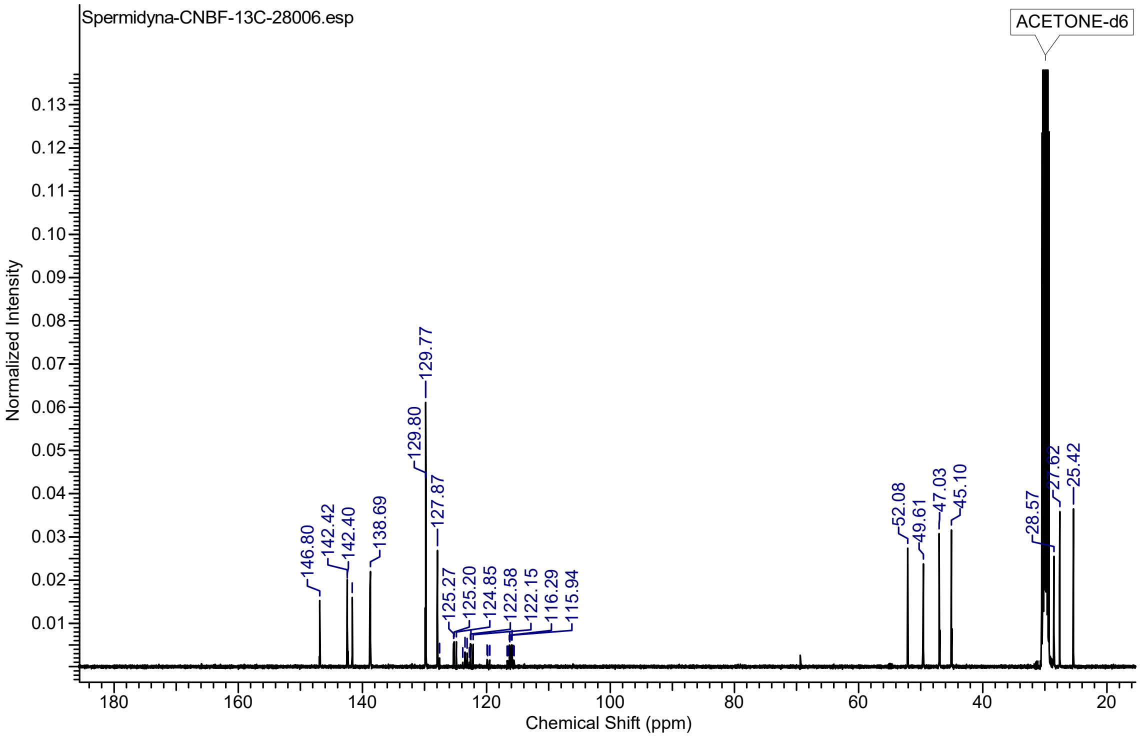


**Spm-CNBF** ($^{1}$H NMR)

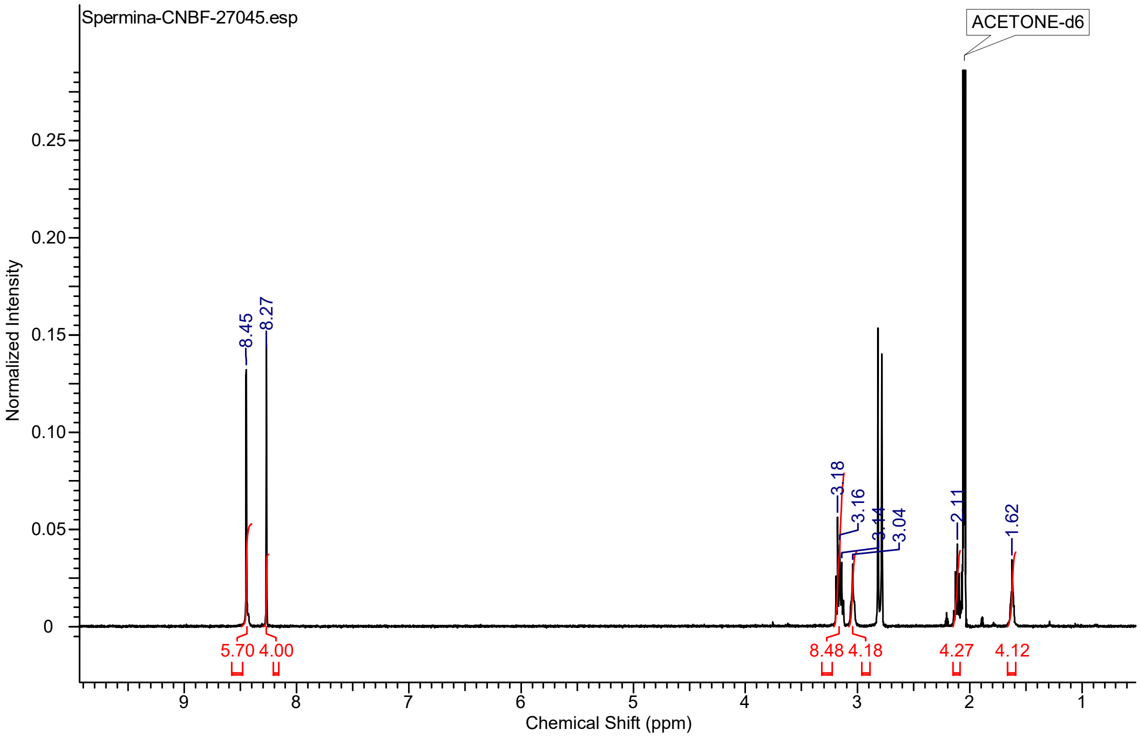

**Spm-CNBF** ($^{19}F$ NMR)

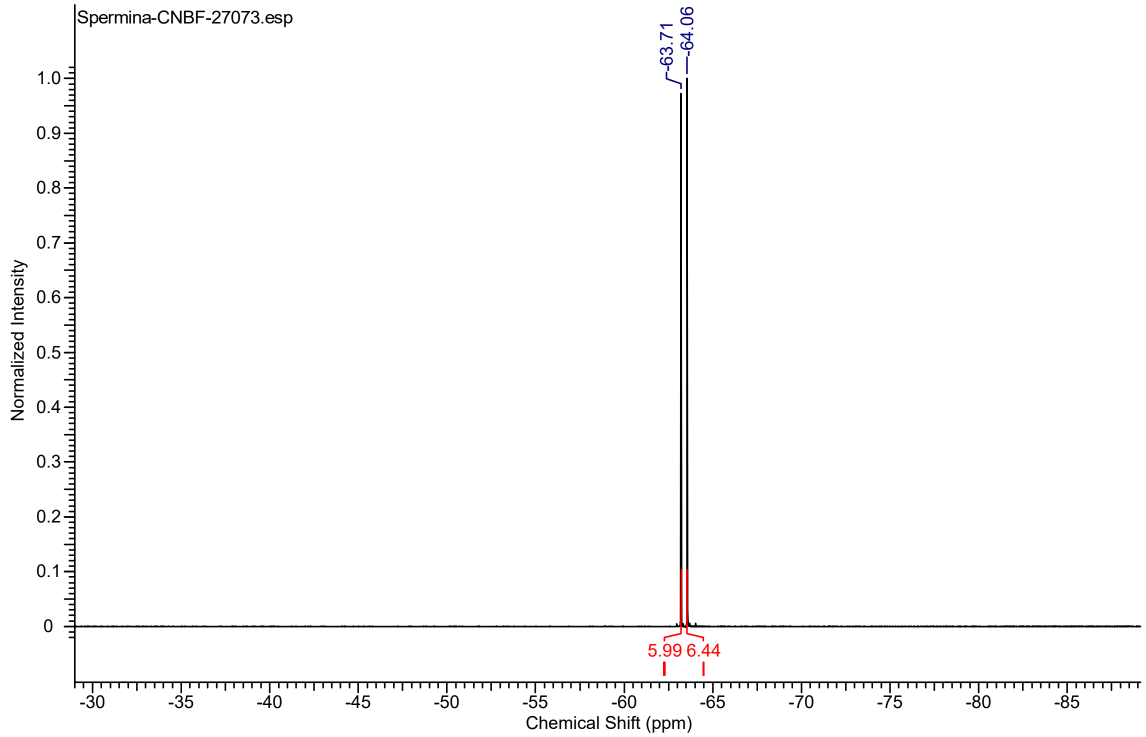


**Spm-CNBF** ($^{13}C$ NMR)

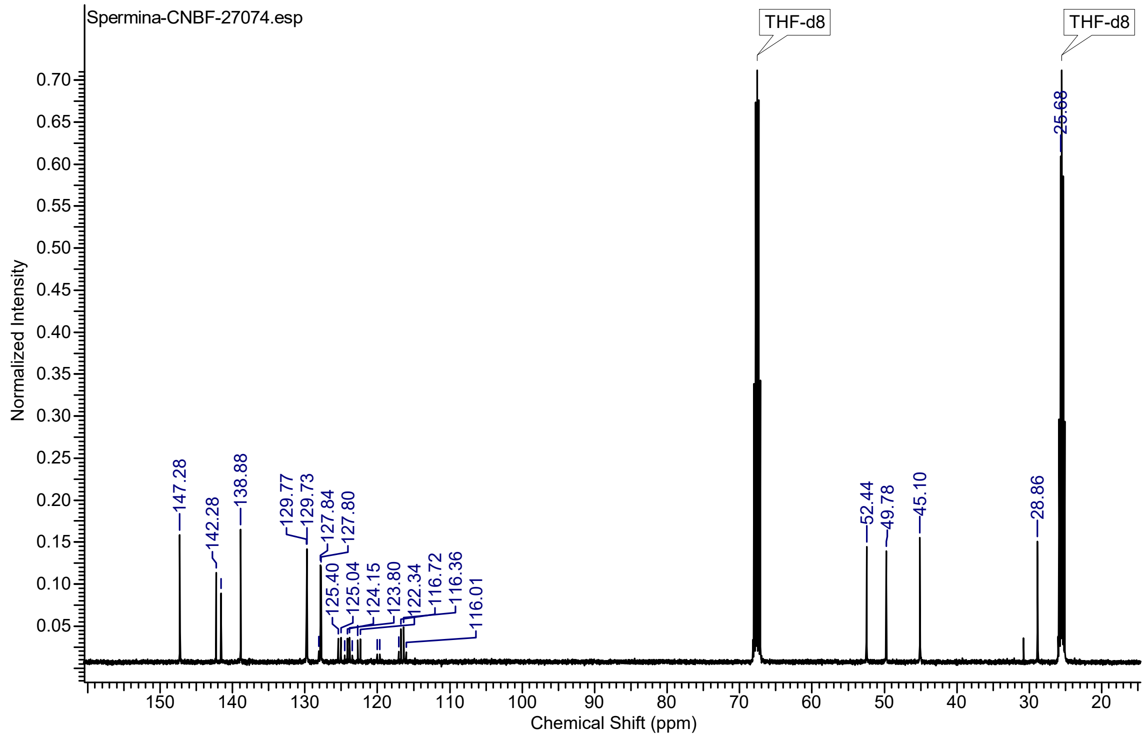

**Put-BPI** ($^{1}$H NMR)

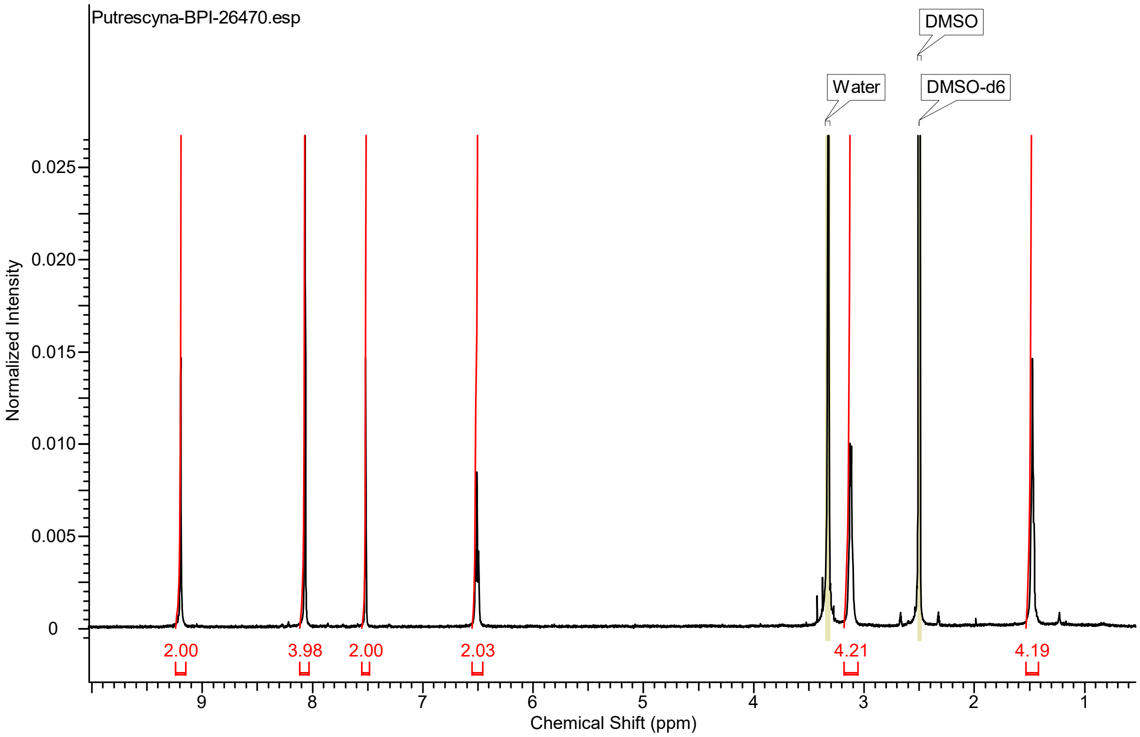


**Put-BPI** ($^{19}$F NMR)

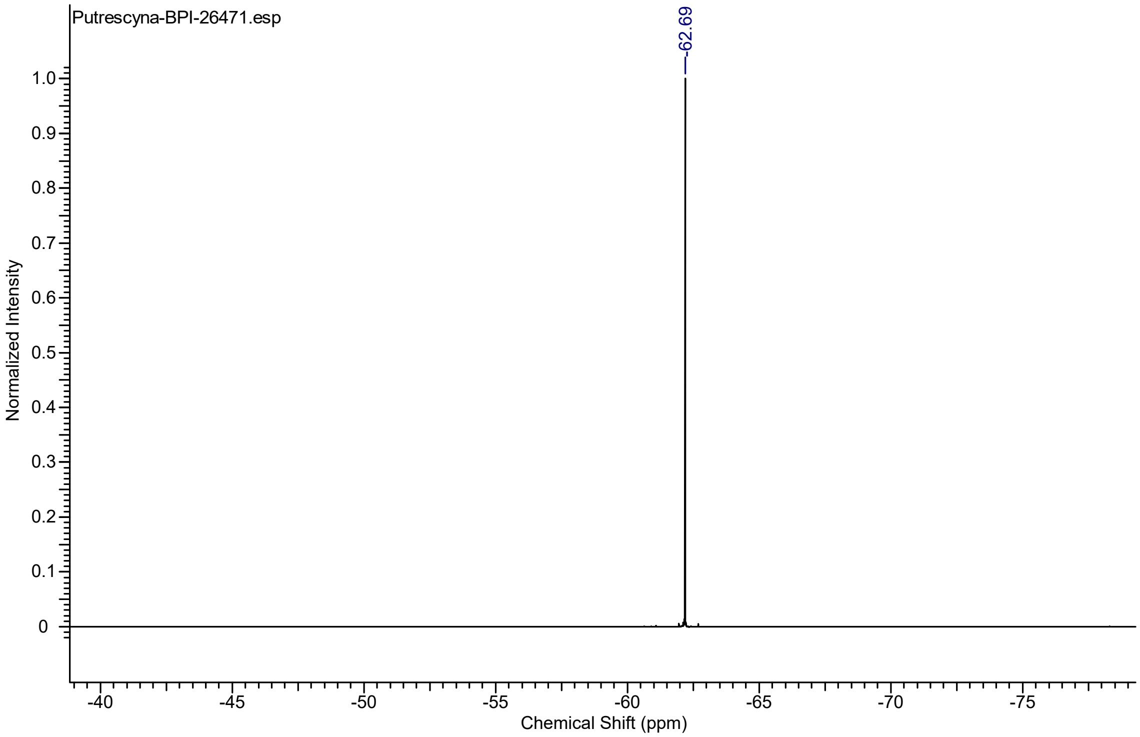

**Put-BPI** ($^{13}$C NHR)

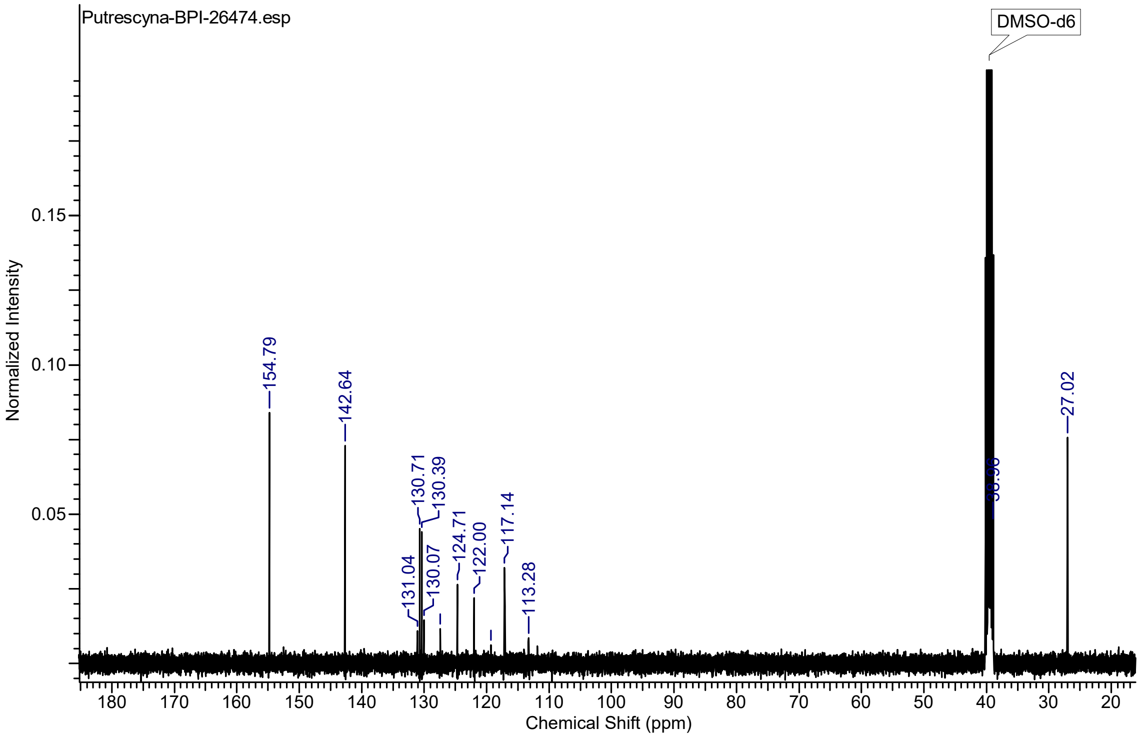


**Spm-BPI** ($^{1}$H NMR)

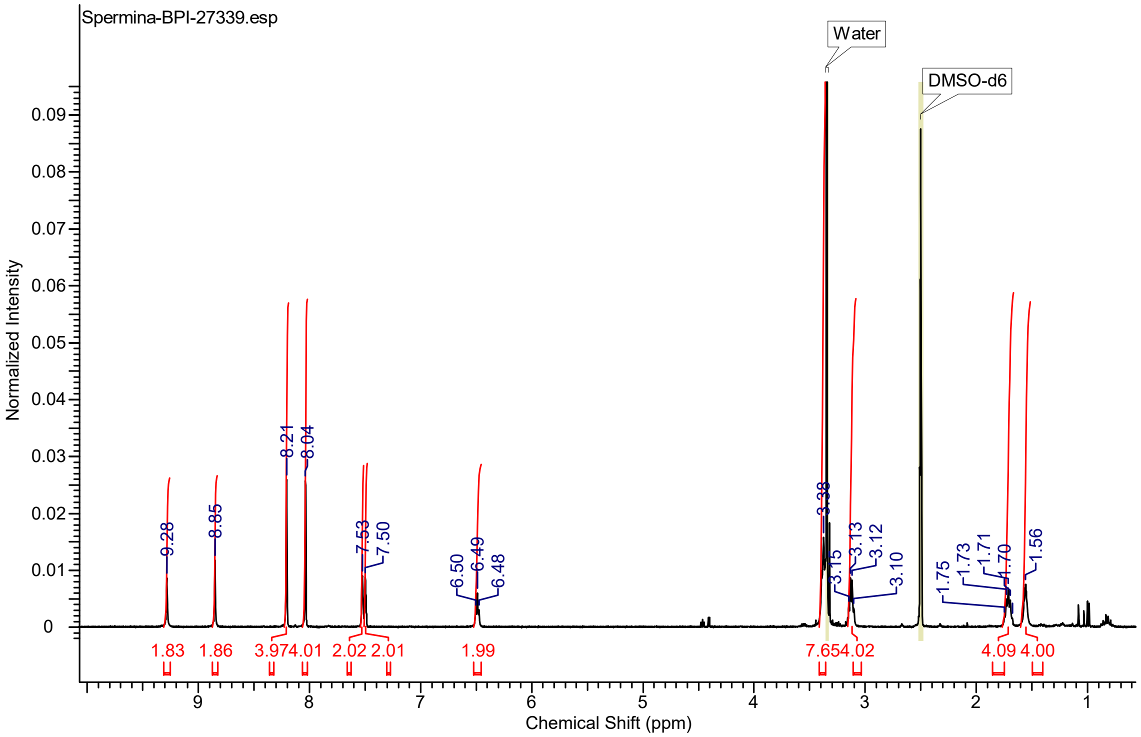

**Spm-BPI** ($^{19}F$ NMR)

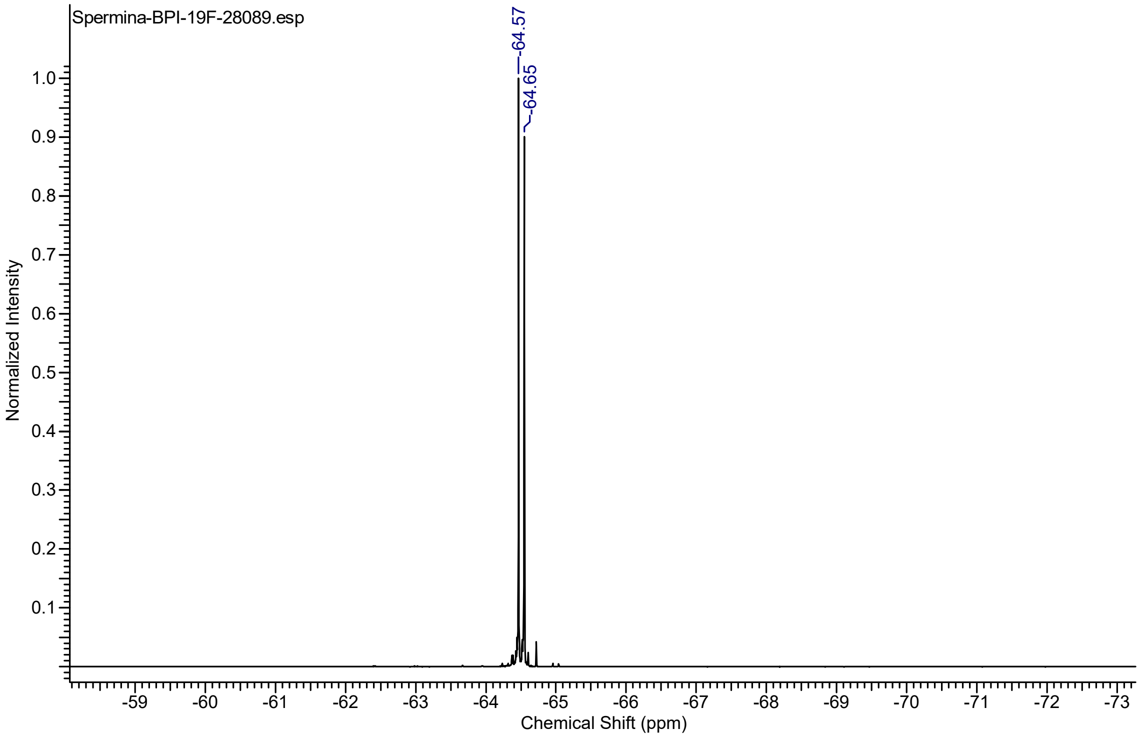


**Spm-BPI** ($^{13}C$ NMR)

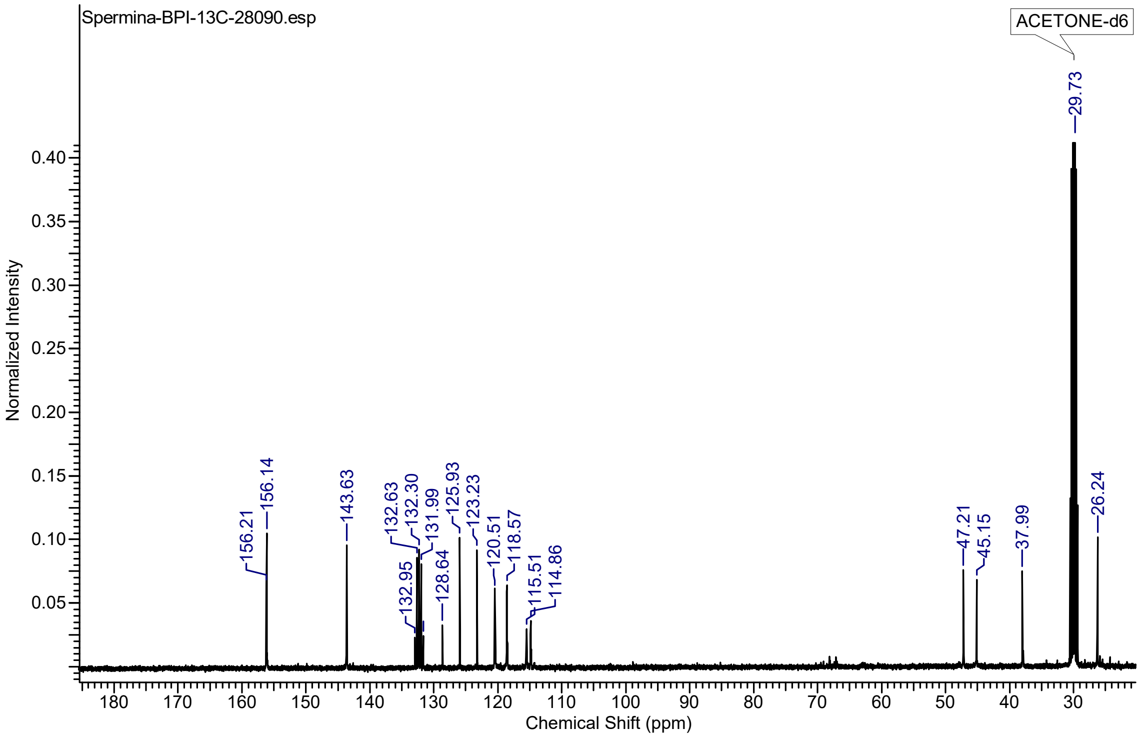

# IR-ATR spectra

**Cad-BPI**

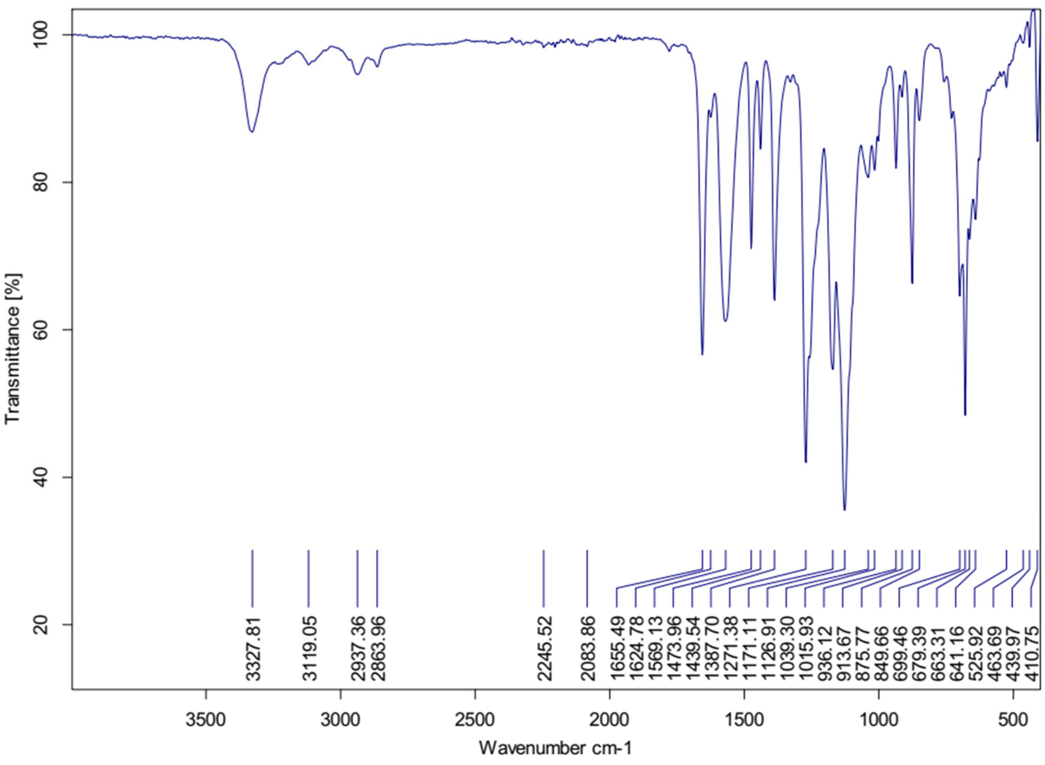


**Put-BPI**

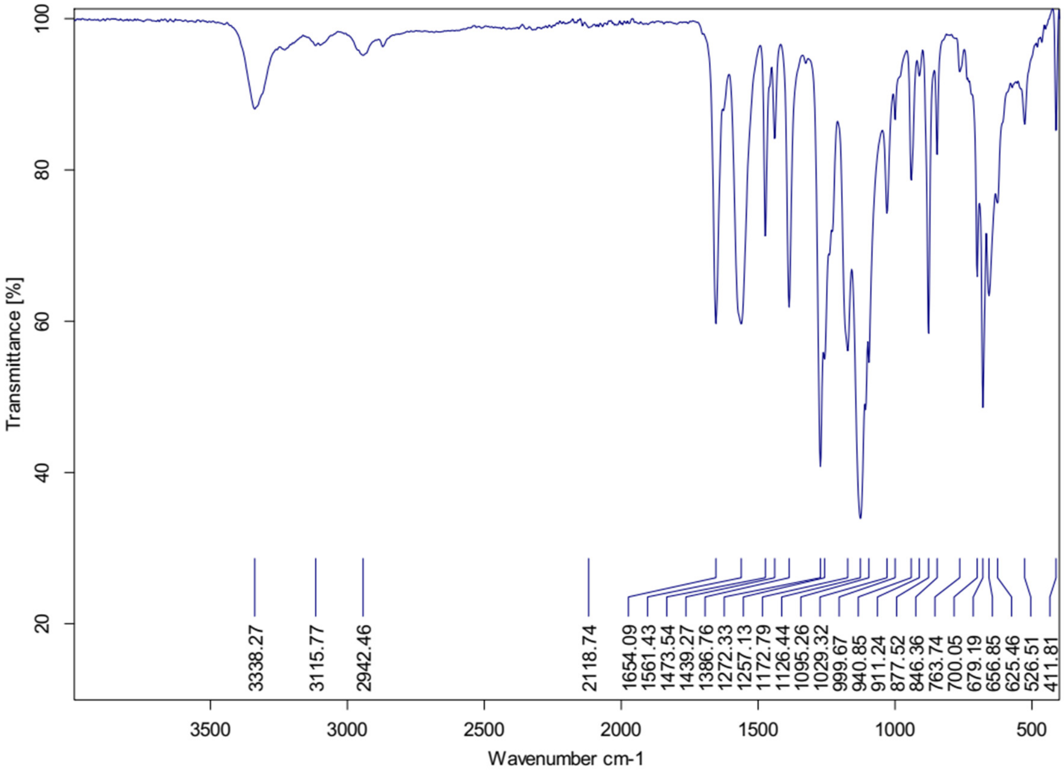

**Spm-BPI**

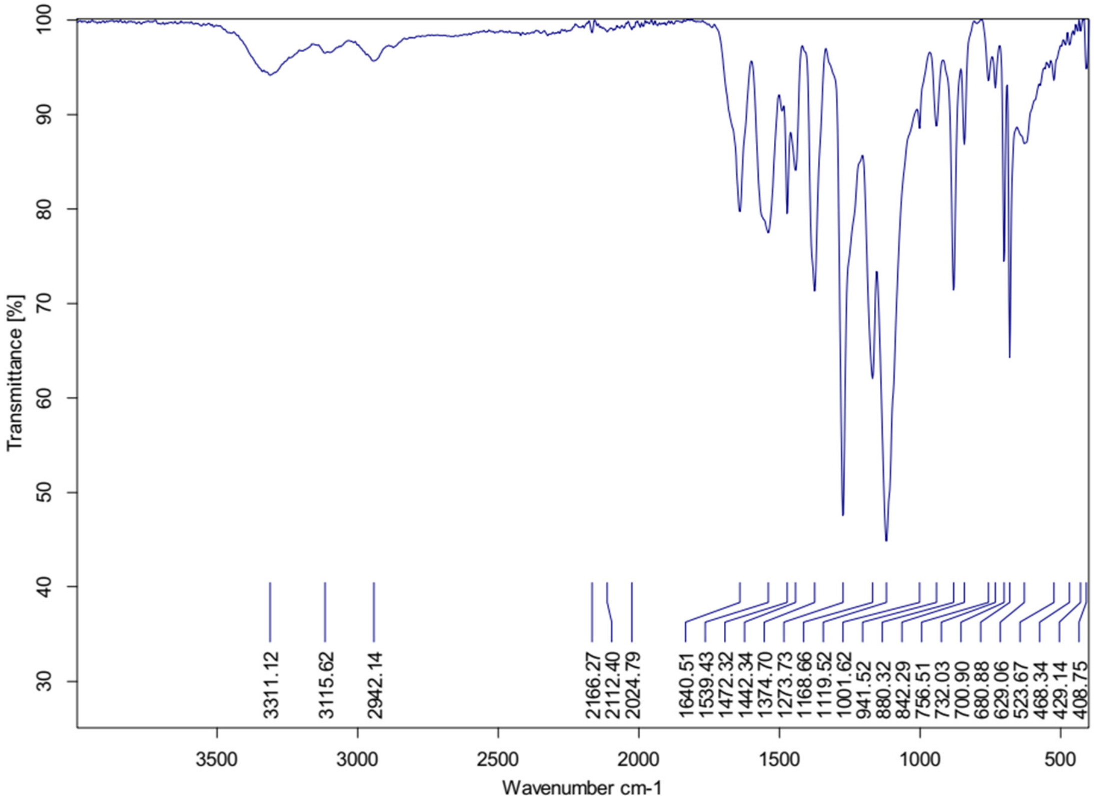


**Spd-BPI**

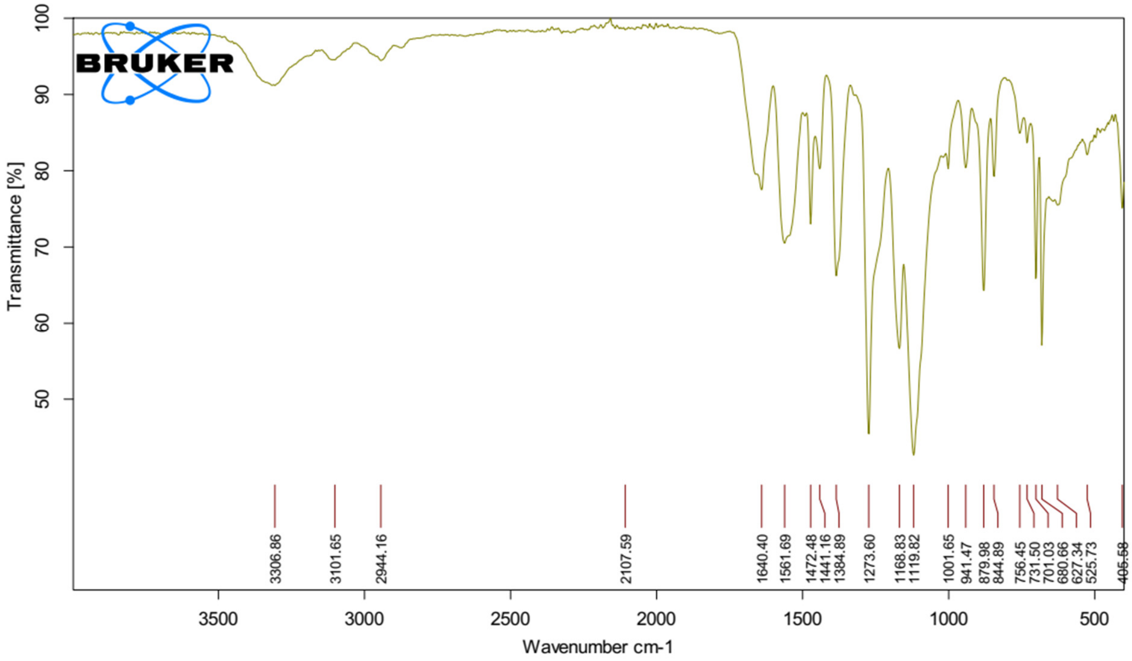

**Cad-CNBF**

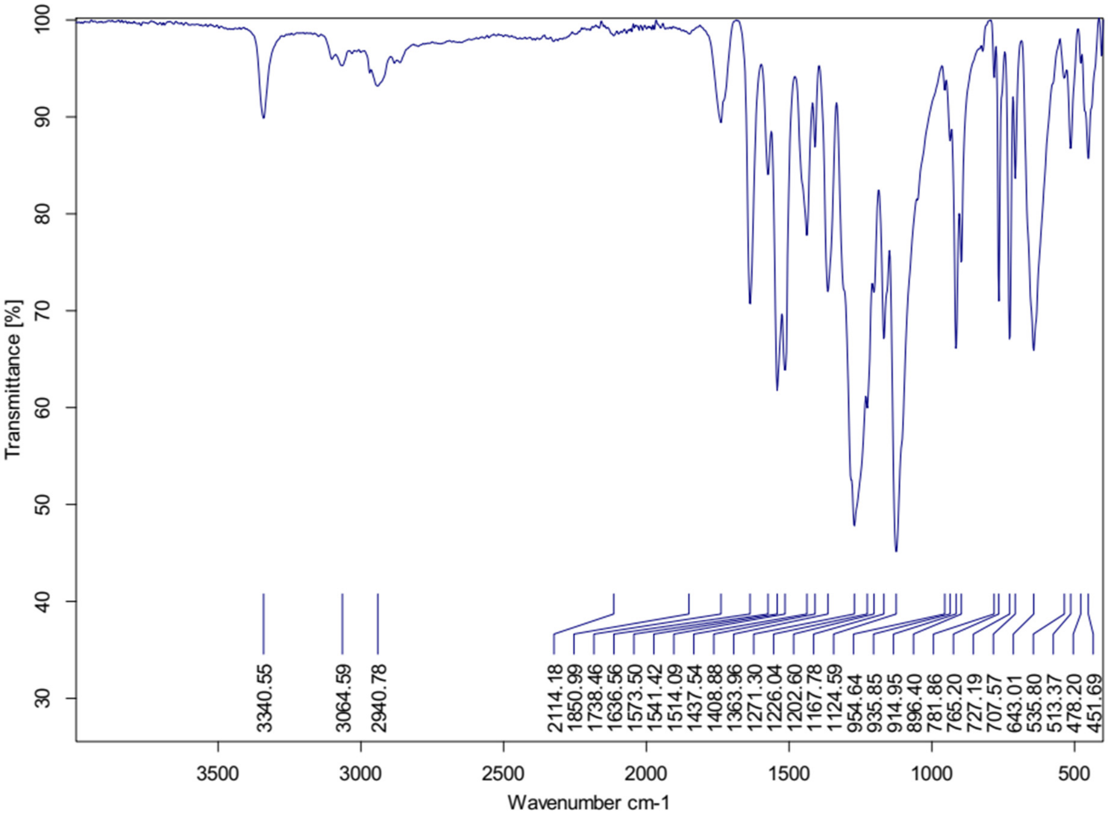


**Put-CNBF**

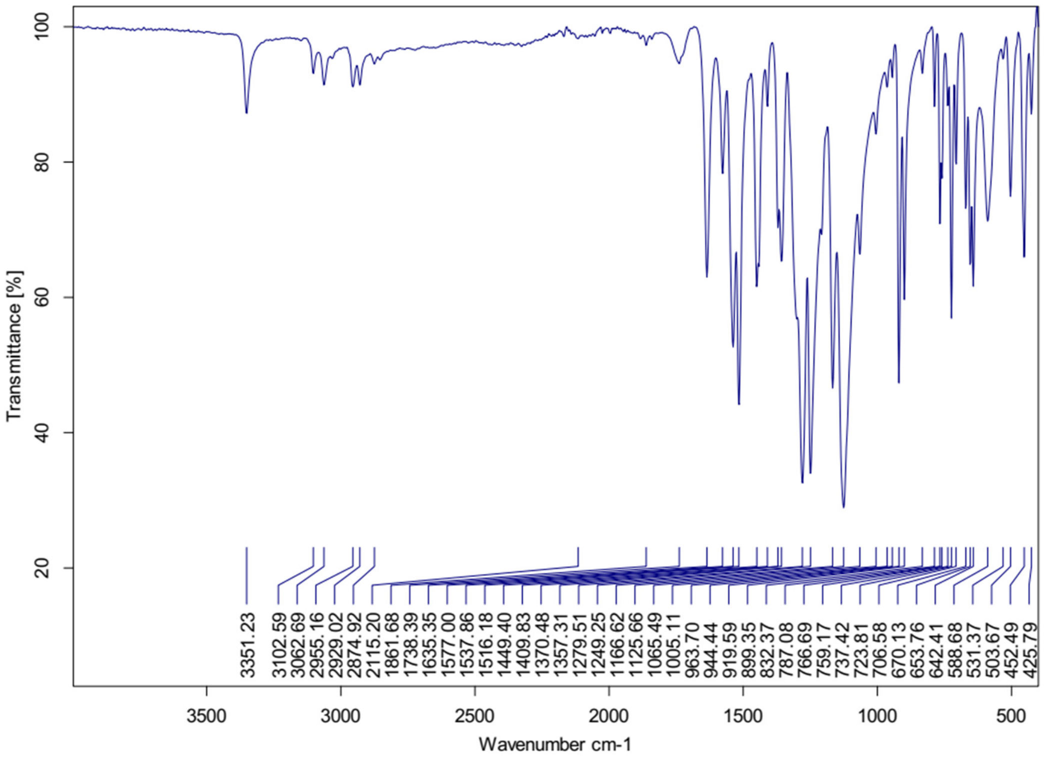

**Spm-CNBF**

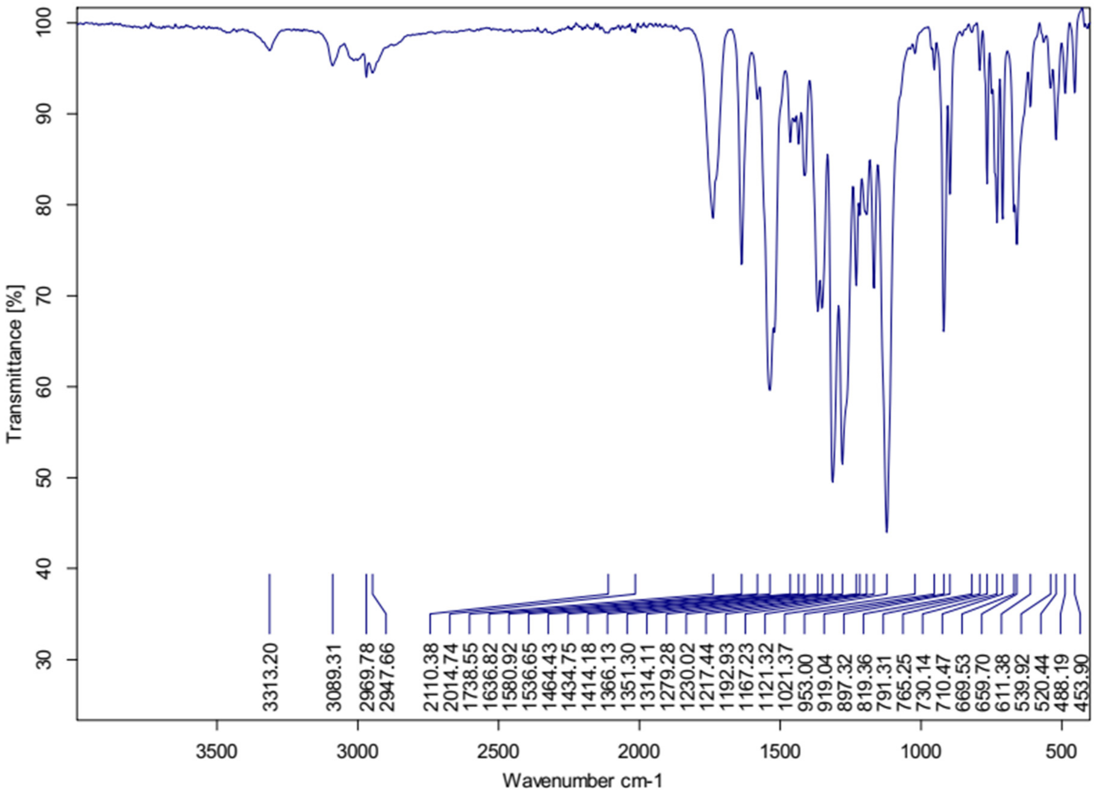


**Spd-CNBF**

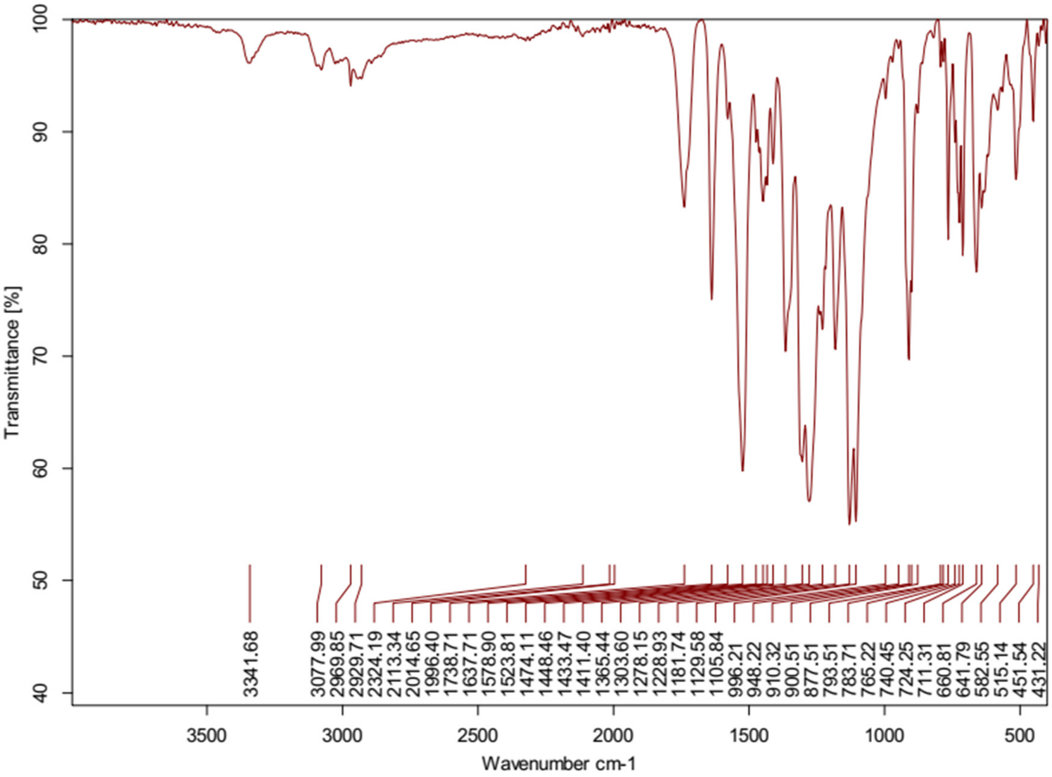

**Cad-FNBT**

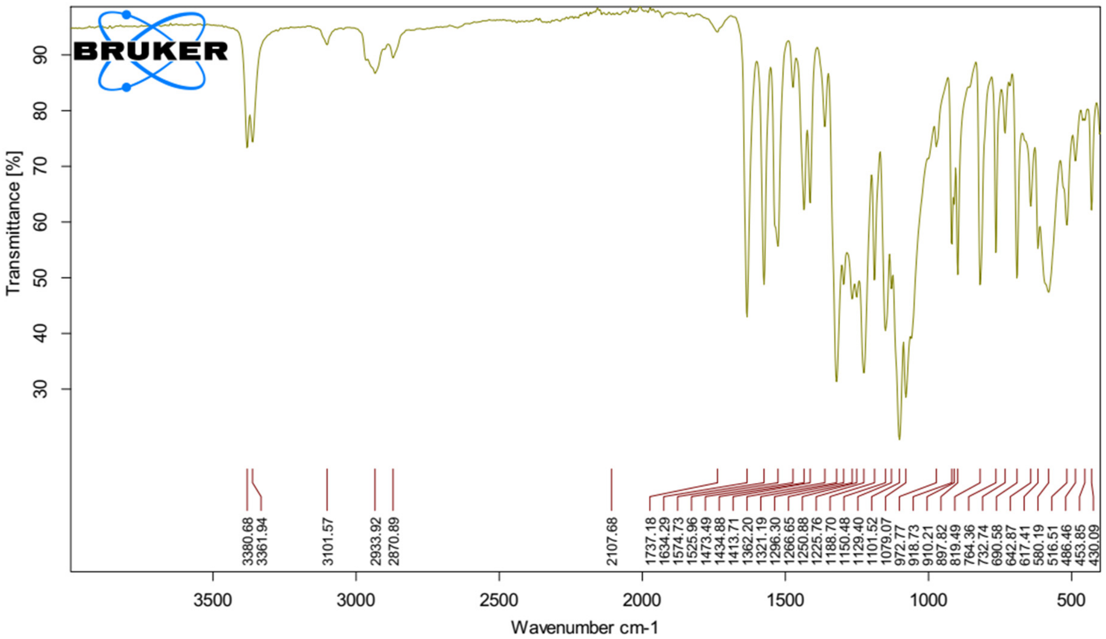


**Put-FNBT**

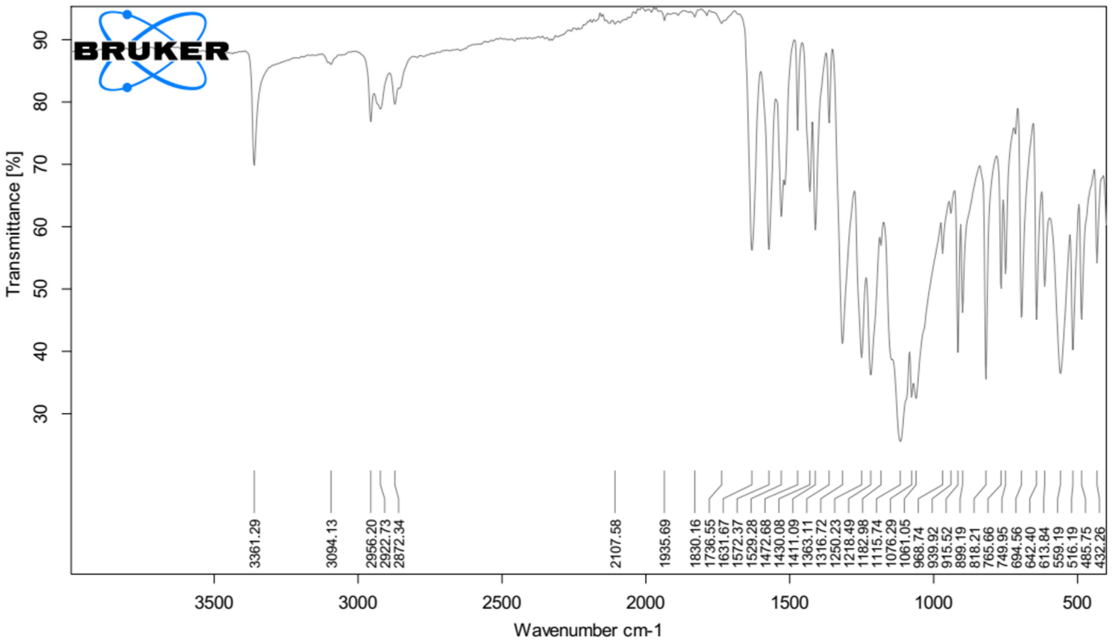

**Spm-FNBT**

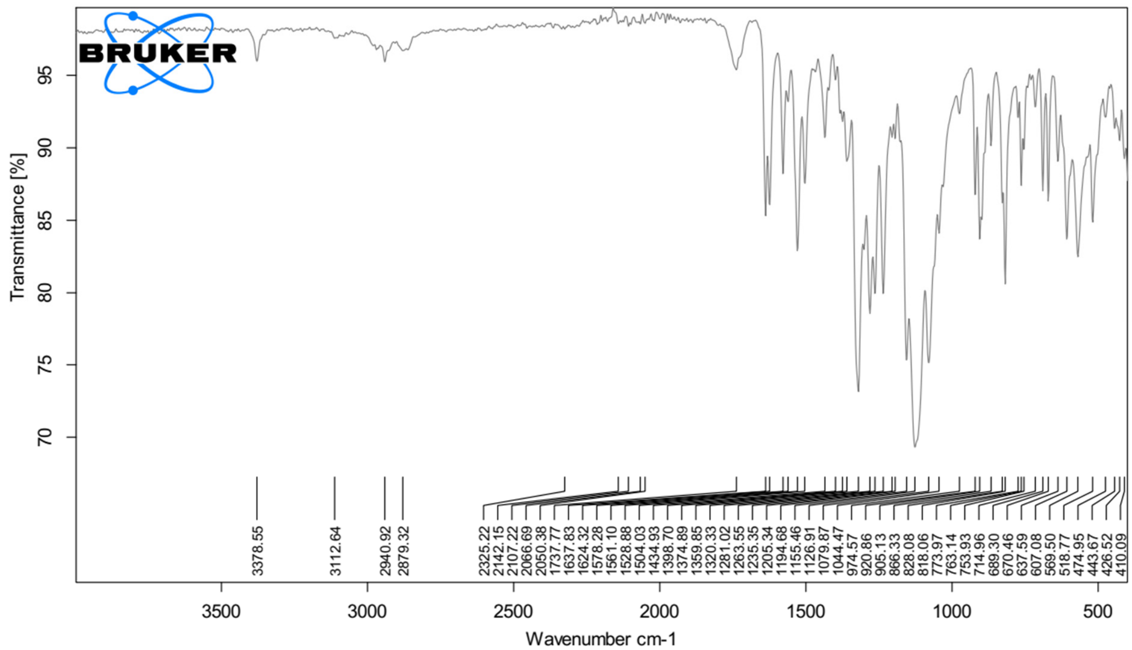


**Spd-FNBT**

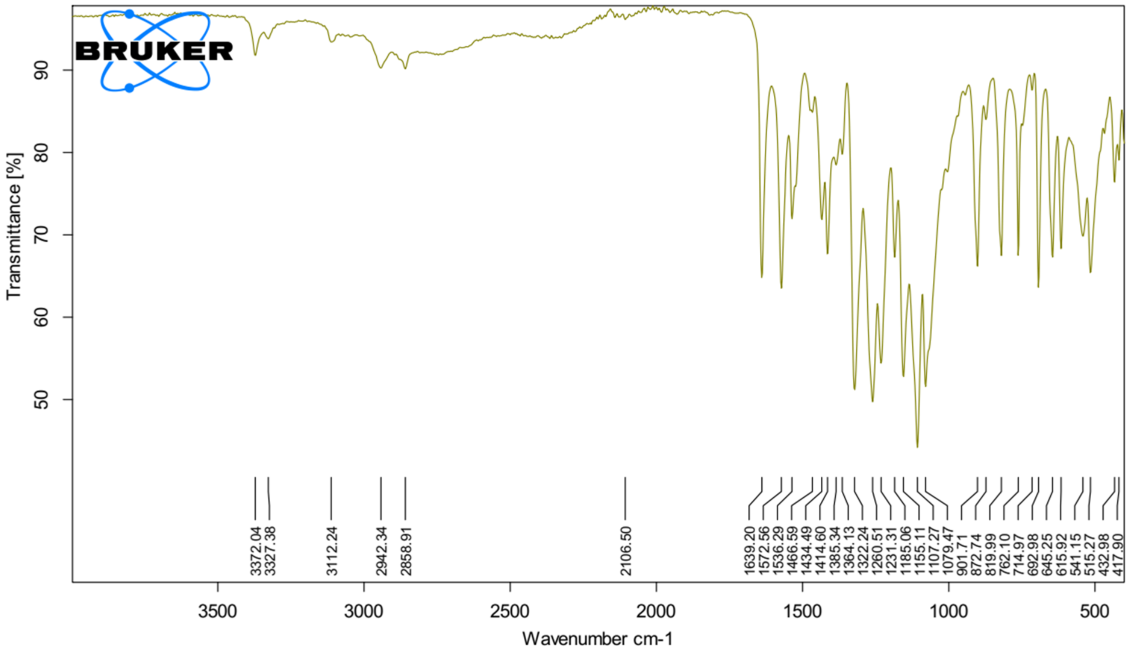

**Table S1.** The results of the data collections and refinement for Cad-FNBT; Put-FNBT; Spm-FNBT and Spd-CNBF.

| **Identification code** | **Spm-FNBT** | **Cad-FNBT** | **Put-FNBT** | **Spd-CNBF** |
|---|---|---|---|---|
| Empirical formula | $C_{38} H_{34} F_{12} N_8 O_8$ | $C_{19} H_{18} F_6 N_4 O_4$ | $C_{18} H_{16} F_6 N_4 O_4$ | $C_{28} H_{22} F_9 N_9 O_{12}$ |
| Formula weight | 958.73 | 480.37 | | |
| Temperature | 100(2) K | 100(2) K | 100(2) K | 100(2) K |
| Wavelength [Å] | 1.54184 | 1.54184 | 1.54184 | 0.7999 |
| Crystal system, space group | Triclinic, P-1 | Monoclinic, P2(1)/n | Triclinic, P-1 | Triclinic, P-1 |
| Unit cell dimensions [Å] and [°] | a = 7.9650(3) | a = 9.93377(12) | a = 4.9668(2) | a = 7.9750(16) Å |
| | α= 71.646(3) | α= 90° | α= 83.837(3) | α= 75.65(3)°. |
| | b = 11.6008(4) | b = 18.5968(3) | b = 6.5809(3) | b = 11.781(2) Å |
| | β = 83.951(3) | β= 100.4050(13) ° | β = 85.011(3) | β= 82.14(3)° |
| | c = 11.7281(4) | c = 11.49761(16) | c = 14.7689(6) | c = 17.767(4) Å |
| | γ = 73.737(3) | γ = 90° | γ = 85.340(3) | γ = 87.48(3)° |
| Volume [$Å^3$] | 987.23(6) | 2089.10(5) | 476.87(4) | 1601.9(6) |

| | | | | |
|---|---|---|---|---|
| Z, calculated density [Mg/m$^3$] | 1, 1.613 | 4, 1.527 | 1, 1.624 | 2, 1.757 |
| Absorption coefficient [mm$^{-1}$] | 1.340 | 1.267 | 1.368 | 0.232 |
| F(000) | 490 | 984 | 238 | 860 |
| Crystal size [mm$^3$] | 0.260 x 0.050 x 0.050 | 0.110 x 0.090 x 0.045 | 0.180 x 0.030 x 0.030 | 0.170 x 0.050 x 0.030 |
| Theta range for data collection | 3.972 to 74.466°. | 4.756 to 74.465°. | 3.019 to 74.322°. | 1.343 to 29.995 |
| Index ranges | -7<=h<=9<br>-14<=k<=14<br>-14<=l<=14 | -12<=h<=12<br>-22<=k<=23<br>-14<=l<=14 | -5<=h<=5<br>-8<=k<=8<br>-17<=l<=17 | -9<=h<=9<br>-14<=k<=14<br>-22<=l<=22 |
| Reflections collected/unique | 11806, 3960 [R(int) = 0.0290] | 8183, 8183 | 4577, 1791 [R(int) = 0.0150] | 20171, 5885 [R(int) = 0.0344] |
| Completeness [%] to theta [°] | 67.684° 99.9 % | 67.684° 96.7% | 67.684° 98.4 % | 28.681°91.9 % |

| | | | | |
|---|---|---|---|---|
| Absorption correction | Analytical | Analytical | Analytical | Semi-empirical from equivalents |
| Max. and min. transmission | 1.00000 and 0.87553 | 0.951 and 0.890 | 1.00000 and 0.88847 | 0.993 and 0.962 |
| Refinement method | Full-matrix least-squares on F2 | Full-matrix least-squares on F2 | Full-matrix least-squares on F2 | Full-matrix least-squares on F2 |
| Data / restraints / parameters | 3960 / 0 / 298 | 8183 / 48 / 326 | 1791 / 0 / 145 | 5885 / 12 / 550 |
| Goodness-of-fit on F2 | 0.969 | 1.054 | 1.061 | 1.063 |
| Final R indices [I>2sigma(I)] | R1 = 0.0359, wR2 = 0.0929 | R1 = 0.0710, wR2 = 0.2045 | R1 = 0.0438, wR2 = 0.1223 | R1 = 0.0426, wR2 = 0.1164 |
| R indices (all data) | R1 = 0.0408, wR2 = 0.0992 | R1 = 0.0857, wR2 = 0.2197 | R1 = 0.0472, wR2 = 0.1252 | R1 = 0.0453, wR2 = 0.1192 |
| Extinction coefficient | n/a | n/a | n/a | n/a |
| Largest diff. peak and hole [eÅ$^{-3}$] | 0.364 and -0.359 | 0.664 and -0.559 | 0.610 and -0.430 | 0.620 and -0.556 |

**Table S2.** Precision and accuracy of the proposed procedures.

| | Put | | | Cad | | | Spd | | | Spm | | |
|---|---|---|---|---|---|---|---|---|---|---|---|---|
| X1 [mg·L$^{-1}$] | X2 [mg·L$^{-1}$] | Rec [%] | CV [%] | X2 [mg·L$^{-1}$] | Rec [%] | CV [%] | X2 [mg·L$^{-1}$] | Rec [%] | CV [%] | X2 [mg·L$^{-1}$] | Rec [%] | CV [%] |
| CNBF | | | | | | | | | | | | |
| 5.25 | 5.16 | 98.32 | 0.72 | 5.02 | 95.54 | 3.04 | 4.88 | 92.91 | 1.27 | 4.958 | 94.44 | 3.04 |
| 7.50 | 7.37 | 98.32 | 1.19 | 7.24 | 96.56 | 1.38 | 7.01 | 93.44 | 1.35 | 6.968 | 92.91 | 3.69 |
| 10.50 | 10.25 | 97.64 | 1.90 | 10.01 | 95.29 | 2.41 | 9.88 | 94.09 | 1.24 | 9.838 | 93.69 | 5.05 |
| 15.25 | 15.01 | 98.44 | 0.53 | 14.45 | 94.75 | 3.44 | 14.50 | 95.07 | 2.75 | 14.84 | 97.31 | 1.312 |
| 19.20 | 18.96 | 98.47 | 0.51 | 18.38 | 95.480 | 2.13 | 17.75 | 92.21 | 1.34 | 18.752 | 97.41 | 1.32 |
| FNBT | | | | | | | | | | | | |
| 5.25 | 5.13 | 97.79 | 1.87 | 5.07 | 96.49 | 1.69 | 5.07 | 96.61 | 2.24 | 5.11 | 97.33 | 1.63 |
| 7.50 | 7.41 | 98.77 | 0.82 | 7.35 | 98.08 | 1.01 | 7.21 | 96.11 | 0.55 | 7.35 | 97.95 | 1.41 |
| 10.50 | 10.24 | 97.52 | 1.67 | 10.13 | 96.46 | 0.97 | 10.07 | 95.87 | 1.16 | 10.26 | 97.68 | 1.39 |
| 15.25 | 15.10 | 98.99 | 0.53 | 15.10 | 99.03 | 0.93 | 15.14 | 99.25 | 0.36 | 15.10 | 99.03 | 0.74 |
| 19.20 | 19.10 | 99.22 | 0.41 | 19.11 | 99.28 | 0.46 | 19.07 | 99.08 | 0.47 | 19.12 | 99.31 | 0.61 |

*Where: X1 -experimental concentration, X2 - found concentration, Rec - recovery was calculated as mean value of (experimental concentration/found concentration)·100%,* CV - *coefficient of variation [%]*

## Put-FNBT

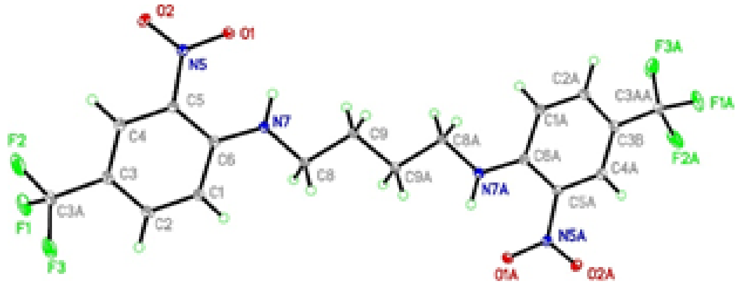


## Cad-FNBT

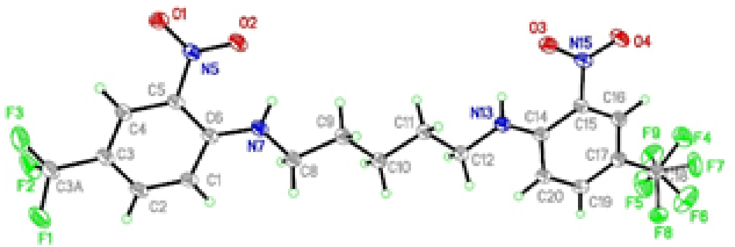


## Spm-FNBT

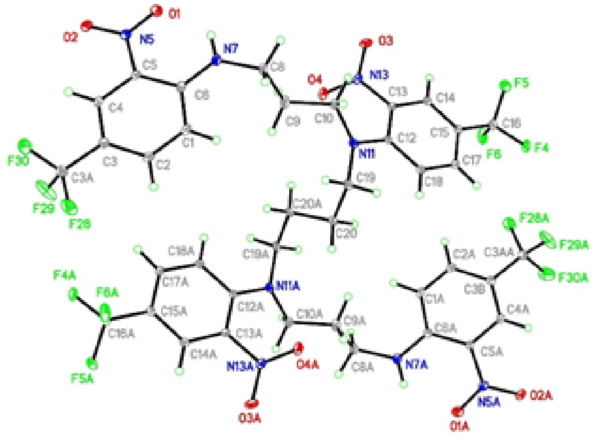

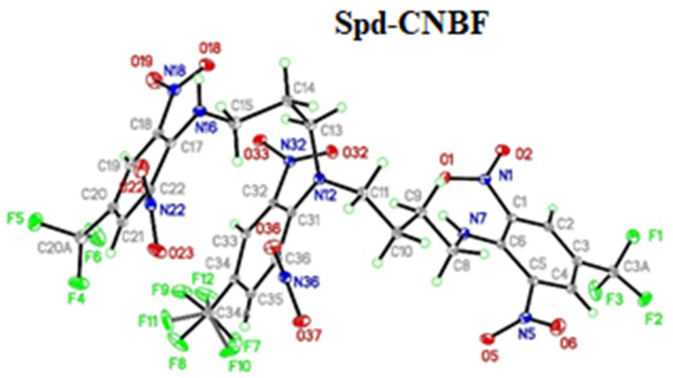


**Figure S1**. Molecular structures of polyamine derivatives.

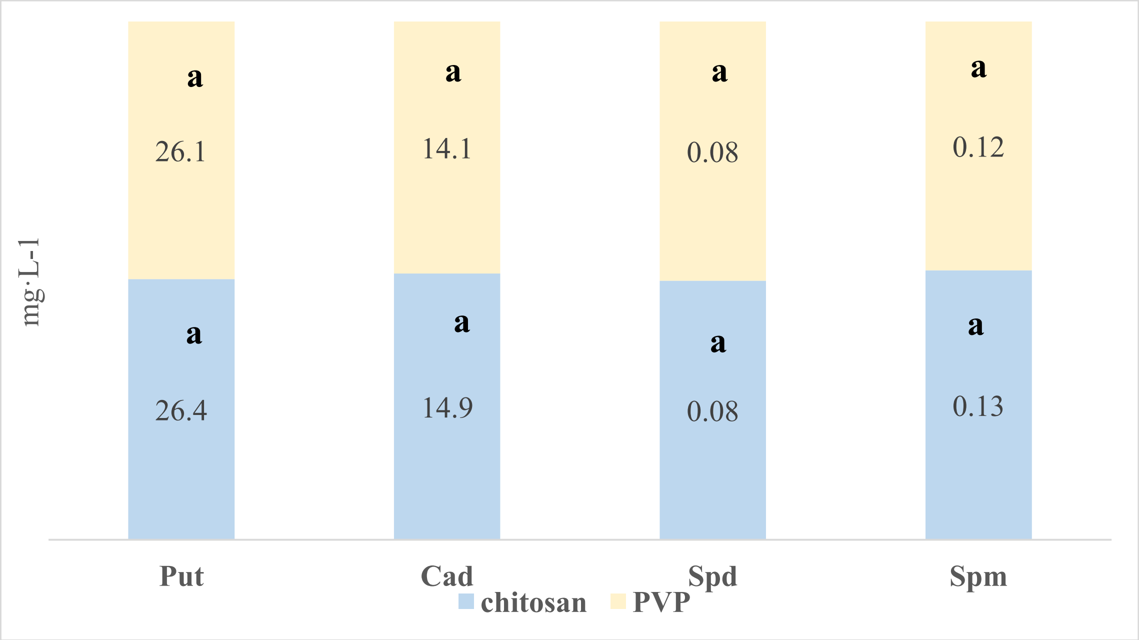


**Figure S2.** Results of biogenic amines chromatographic determination in wine samples after FNBT derivatization (sample 1, Cascade, red sweet wine) and purification procedures with chitosan, and PVP; *the letter (a) for the same polyamine indicate no significant differences (one-way ANOVA and Duncan test, $p<0.05$).*

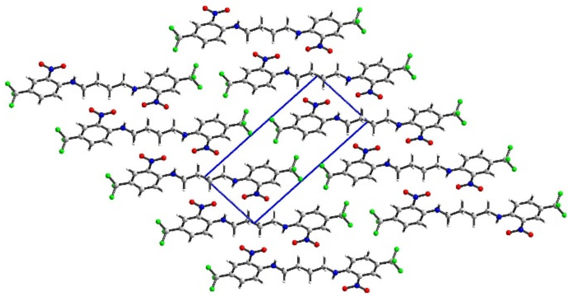

**Figure S3.** Packing of Put-FNBT along *a* axis.

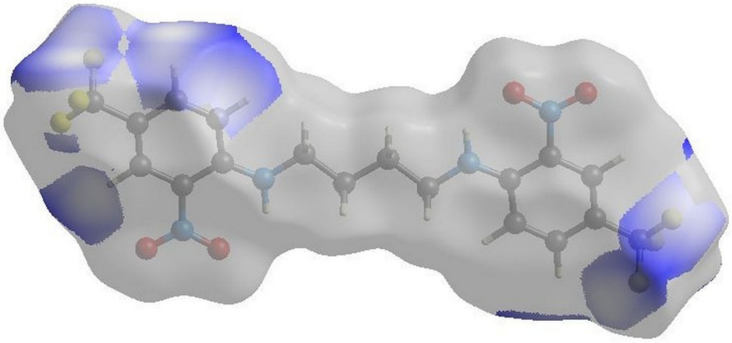

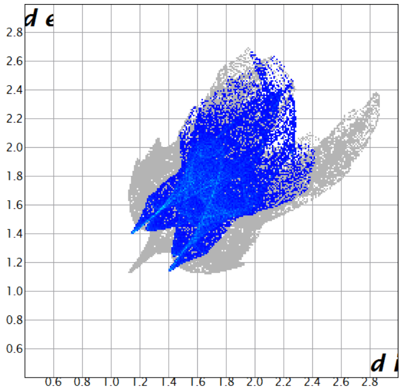


a b

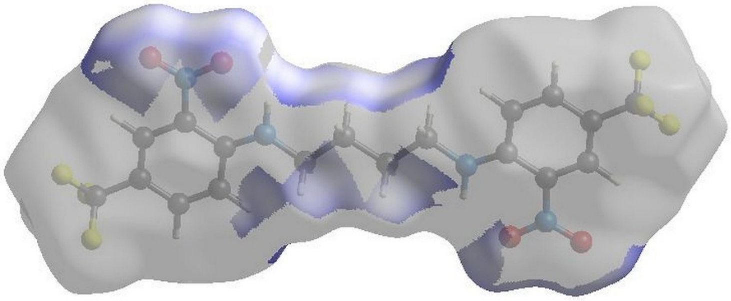

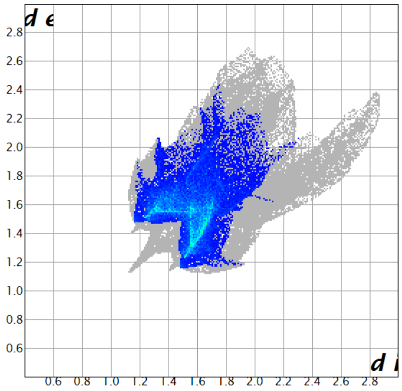


c d

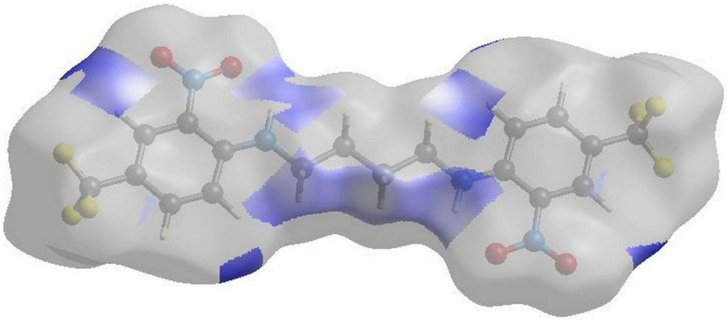

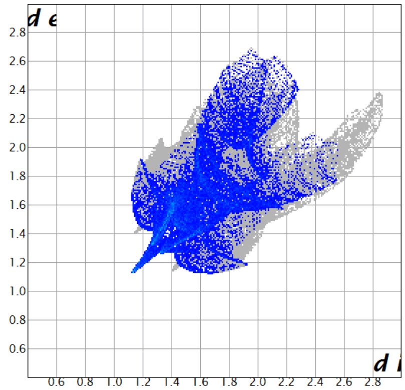


e f

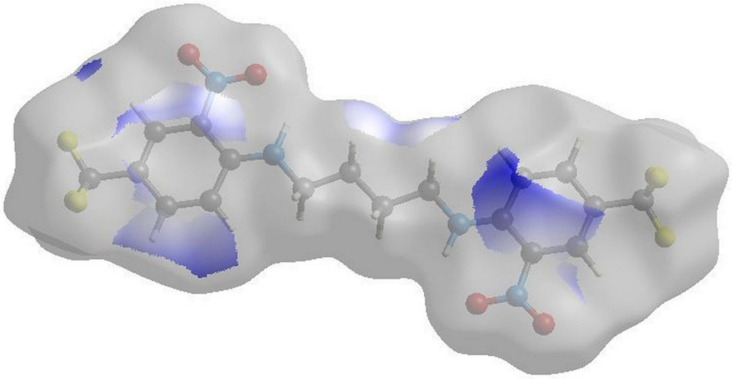

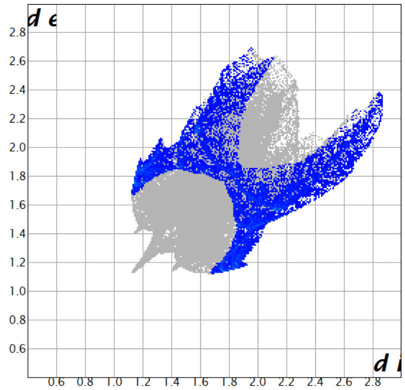


g h

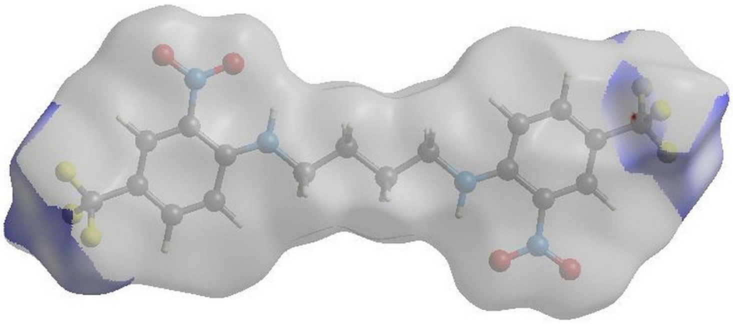

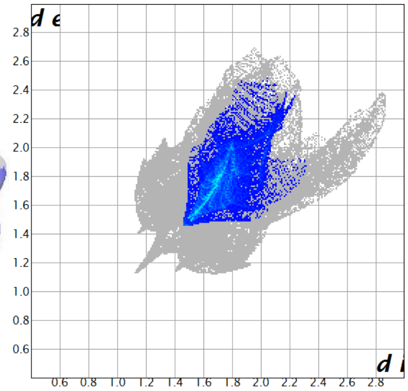


i j

**Figure S4.** Hirshfeld surfaces and fingerprints of selected interactions created in the crystal network of Put-FNBT: Hirshfeld surface (a) and fingerprint (b) for F…H (20.8%), Hirshfeld surface (c; red markers correspond to the spike at 1.25; 0.95) and fingerprint (d) for O…H (24.4%), Hirshfeld surface (e) and fingerprint (f) for H…H (14.8%), Hirshfeld surface (g) and

fingerprint (h) for H…C (10.6%), Hirshfeld surface (i) and fingerprint (j) for F…F (14.1%). In brackets, there is given surface area included as a percentage of the total surface area.

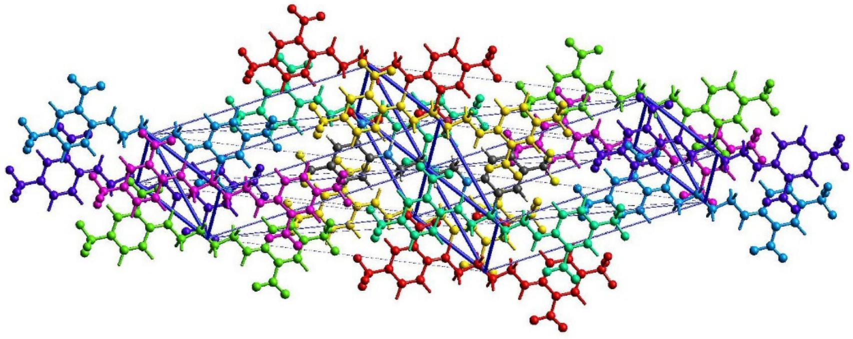

a

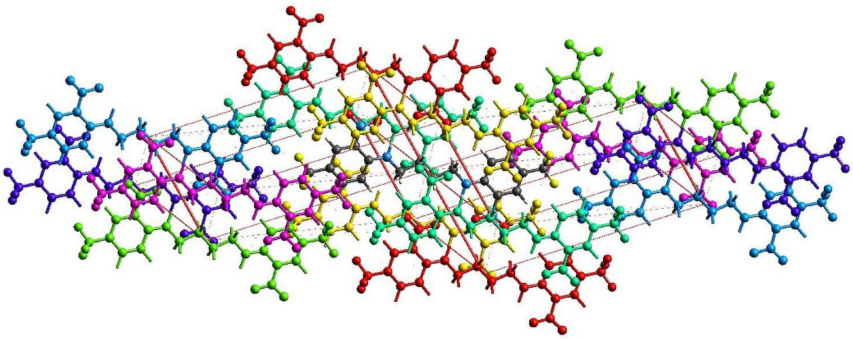

b

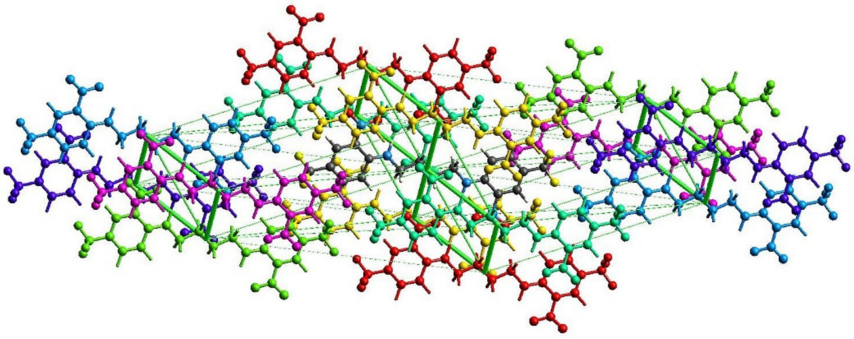

c

| | R | Electron Density | E_ele | E_pol | E_dis | E_rep | E_tot |
|---|---|---|---|---|---|---|---|
| | 8.56 | B3LYP/6-31G(d,p) | -33.5 | -8.7 | -29.5 | 22.8 | -53.5 |
| | 4.97 | B3LYP/6-31G(d,p) | -1.2 | -7.3 | -79.9 | 43.9 | -49.2 |
| | 17.00 | B3LYP/6-31G(d,p) | -9.5 | -1.0 | -12.5 | 0.0 | -21.6 |
| | 6.58 | B3LYP/6-31G(d,p) | -15.0 | -7.8 | -73.9 | 41.3 | -60.5 |

| | | | | | | | |
|---|---|---|---|---|---|---|---|
| | 20.81 | B3LYP/6-31G(d,p) | 0.3 | -0.1 | -5.8 | 0.0 | -4.8 |
| | 20.84 | B3LYP/6-31G(d,p) | 1.5 | -0.1 | -7.2 | 0.0 | -4.8 |
| | 16.80 | B3LYP/6-31G(d,p) | -3.1 | -0.5 | -12.6 | 0.0 | -14.7 |

**Figure S5.** Interactions energy (total – a, electrostatic – b, dispersion – c) in the crystal network of Put-FNBT are given in kJ/mol. The tube diameter depends on the interactions energy. The color codding and the interactions energy are given in the table (d); R is the distance between molecular centroids in Å.

**Table S3.** Linear regression calibration parameters of BAs determination by RP-HPLC method after derivatives with FNBT (n =3[a]).

| | t ± SD [min] | $R^2$ | DL [mg·L$^{-1}$] | QL [mg·L$^{-1}$] |
|---|---|---|---|---|
| Histamine | 6.73 ±0.07 | 0.9991 | 1.01 | 3.86 |
| Tyramine | 9.71 ±0.02 | 0.999 | 1.00 | 3.34 |
| Tryptamine | 13.84 ±0.02 | 0.9990 | 1.16 | 3.87 |
| Phenylethylamine | 15.93 ±0.03 | 0.9995 | 0.66 | 2.19 |

Where: *t- retention time; SD - standard deviation,* $R^2$ *- coefficient of determination; n- number of samples; DL (Detection Limit) [mg·L*$^{-1}$*] =* $(3\times s_{y/x})\times b^{-1}$*; QL (Quantification Limit) [mg·L*$^{-1}$*] =* $(10\times s_{y/x})\times b^{1-}$*;* [a]*- each concentration level was analyzed in triplicate*

**Table S4.** Results of aromatic and heterocyclic biogenic amines determination **(X ± SD) [mg·L$^{-1}$]** in tested wine samples.

| Wine sample | **Him** | **Tym** | **Try** | **Phen** |
|---|---|---|---|---|
| Cascade, sweet red wine | 7.04±0.05 | 1.24±0.01 | 0.10±0.01 | 0.78±0.01 |
| Maréchal Foch, sweet red wine | 8.62±0.04 | 1.90±0.02 | nd | 1.55±0.01 |
| Agat Donskoy, sweet, red wine (Enovini yeast) | 12.4±0.06 | 3.35±0.03 | 0.11±0.01 | 2.96±0.02 |
| Agat Donskoy, sweet red wine (Bordeaux yeast) | 8.70±0.19 | 0.89±0.01 | 0.025±0.004 | 0.43±0.01 |
| Aurora and Arcadia, sweet white wine | 4.90±0.02 | 7.02±0,14 | 0.14±0.03 | 2.94±0.01 |
| Aurora, semi-sweet white wine | 3.63±0.02 | nd | 0.94±0.03 | 0.83±0.02 |
| elderberry wine | 5.27±0.04 | 2.06±0.01 | nd | 2.01±0.03 |
| chokeberry wine | 2.81±0.01 | nd | nd | 0.51±0.01 |
| apple wine | 4.11±0.03 | 2.51±0.04 | nd | 0.41±0.01 |

*Where: X - mean value; SD – standard deviation, number of independent sample = 3.*
