# Supplementary material for: The Selection of the Best Derivatization Reagents for the Determination of Polyamines in Home-Made Wine Samples
Source: Materials (Basel). 2023 Feb 9;16(4):1474. doi: 10.3390/ma16041474 (PMC9960030; doi:10.3390/ma16041474)

checkCIF/PLATON report

Structure factors have been supplied for datablock(s) 14be55-p-1_a

THIS REPORT IS FOR GUIDANCE ONLY. IF USED AS PART OF A REVIEW PROCEDURE FOR PUBLICATION, IT SHOULD NOT REPLACE THE EXPERTISE OF AN EXPERIENCED CRYSTALLOGRAPHIC REFEREE.

No syntax errors found. CIF dictionary Interpreting this report

Datablock: 14be55-p-1_a

```
Bond precision:   C-C = 0.0022 A          Wavelength=0.79990

Cell:            a=7.9750(16)       b=11.781(2)       c=17.767(4)
                 alpha=75.65(3)     beta=82.14(3)     gamma=87.48(3)
Temperature:     100 K

                Calculated                   Reported
Volume          1601.9(6)                    1601.9(6)
Space group     P -1                         P -1
Hall group      -P 1                         -P 1
Moiety formula  C28 H22 F9 N9 O12            C28 H22 F9 N9 O12
Sum formula     C28 H22 F9 N9 O12            C28 H22 F9 N9 O12
Mr              847.55                       847.54
Dx,g cm-3       1.757                        1.757
Z               2                            2
Mu (mm-1)       0.227                        0.232
F000            860.0                        860.0
F000'           860.97
h,k,lmax        9,14,22                      9,14,22
Nref            6540                         5885
Tmin,Tmax       0.986,0.993                  0.962,0.993
Tmin'           0.961

Correction method= # Reported T Limits: Tmin=0.962 Tmax=0.993
AbsCorr = MULTI-SCAN

Data completeness= 0.900          Theta(max)= 29.995

R(reflections)= 0.0426( 5436)                       wR2(reflections)=
                                                    0.1192( 5885)
S = 1.063                   Npar= 550
```

The following ALERTS were generated. Each ALERT has the format
test-name_ALERT_alert-type_alert-level.
Click on the hyperlinks for more details of the test.

Alert level A

```
PLAT029_ALERT_3_A _diffrn_measured_fraction_theta_full value Low .      0.919 Why?
```

Author Response: The data were collected on MX14-2 beamline (Bessy II, Berlin) and spindle axis rotation was the only available strategy resulting in slightly lowered completeness for triclinic and sometimes also monoclinic crytals.

Alert level B

```
PLAT430_ALERT_2_B Short Inter D...A Contact  O18      ..O18      .       2.78 Ang.
                                          1-x,-y,-z  =      2_655 Check
PLAT911_ALERT_3_B Missing FCF Refl Between Thmin & STh/L=    0.600        473 Report
```

Alert level C

```
PLAT213_ALERT_2_C Atom F10             has ADP max/min Ratio .....        3.4 prolat
PLAT213_ALERT_2_C Atom F11             has ADP max/min Ratio .....        3.2 prolat
PLAT213_ALERT_2_C Atom F12             has ADP max/min Ratio .....        3.2 prolat
```

Alert level G

```
ABSMU01_ALERT_1_G  Calculation of _exptl_absorpt_correction_mu
                not performed for this radiation type.
PLAT003_ALERT_2_G Number of Uiso or Uij Restrained non-H Atoms ...          2 Report
PLAT007_ALERT_5_G Number of Unrefined Donor-H Atoms .............          2 Report
PLAT066_ALERT_1_G Predicted and Reported Tmin&Tmax Range Identical          ? Check
PLAT092_ALERT_4_G Check: Wavelength Given is not Cu,Ga,Mo,Ag,In Ka    0.79990 Ang.
PLAT154_ALERT_1_G The s.u.'s on the Cell Angles are Equal ..(Note)       0.03 Degree
PLAT186_ALERT_4_G The CIF-Embedded .res File Contains ISOR Records          2 Report
PLAT242_ALERT_2_G Low    'MainMol' Ueq as Compared to Neighbors of        C3A Check
PLAT242_ALERT_2_G Low    'MainMol' Ueq as Compared to Neighbors of       C20A Check
PLAT242_ALERT_2_G Low    'MainMol' Ueq as Compared to Neighbors of       C34A Check
PLAT300_ALERT_4_G Atom Site Occupancy of F7         Constrained at        0.7 Check
PLAT300_ALERT_4_G Atom Site Occupancy of F8         Constrained at        0.7 Check
PLAT300_ALERT_4_G Atom Site Occupancy of F9         Constrained at        0.7 Check
PLAT300_ALERT_4_G Atom Site Occupancy of F10        Constrained at        0.3 Check
PLAT300_ALERT_4_G Atom Site Occupancy of F11        Constrained at        0.3 Check
PLAT300_ALERT_4_G Atom Site Occupancy of F12        Constrained at        0.3 Check
PLAT301_ALERT_3_G Main Residue  Disorder ..............(Resd  1  )         5% Note
PLAT432_ALERT_2_G Short Inter X...Y Contact  O22      ..C18      .       2.97 Ang.
                                           -x,-y,-z  =      2_555 Check
PLAT432_ALERT_2_G Short Inter X...Y Contact  O22      ..C19      .       2.98 Ang.
                                           -x,-y,-z  =      2_555 Check
PLAT434_ALERT_2_G Short Inter HL..HL Contact F1       ..F7       .       2.76 Ang.
                                        1-x,1-y,1-z  =      2_666 Check
PLAT434_ALERT_2_G Short Inter HL..HL Contact F1       ..F12      .       2.80 Ang.
```

```
                                                  1+x,1+y,z  =      1_665 Check
PLAT767_ALERT_4_G INS Embedded LIST 6 Instruction Should be LIST 4     Please Check
PLAT860_ALERT_3_G Number of Least-Squares Restraints ............         12 Note
PLAT883_ALERT_1_G No Info/Value for _atom_sites_solution_primary .     Please Do !
PLAT898_ALERT_4_G Second Reported H-M Symbol in CIF Ignored ......          ! Check
PLAT912_ALERT_4_G Missing # of FCF Reflections Above STh/L=  0.600        183 Note
PLAT941_ALERT_3_G Average HKL Measurement Multiplicity ..........         3.4 Low
PLAT965_ALERT_2_G The SHELXL WEIGHT Optimisation has not Converged     Please Check
PLAT978_ALERT_2_G Number C-C Bonds with Positive Residual Density.          7 Info
PLAT984_ALERT_1_G The  C-f'=    0.0033 Deviates from the B&C-Value     0.0043 Check
PLAT984_ALERT_1_G The  F-f'=    0.0186 Deviates from the B&C-Value     0.0225 Check
PLAT984_ALERT_1_G The  N-f'=    0.0063 Deviates from the B&C-Value     0.0082 Check
PLAT984_ALERT_1_G The  O-f'=    0.0113 Deviates from the B&C-Value     0.0142 Check
```

---

1 **ALERT level A** = Most likely a serious problem - resolve or explain
2 **ALERT level B** = A potentially serious problem, consider carefully
3 **ALERT level C** = Check. Ensure it is not caused by an omission or oversight
33 **ALERT level G** = General information/check it is not something unexpected

8 ALERT type 1 CIF construction/syntax error, inconsistent or missing data
14 ALERT type 2 Indicator that the structure model may be wrong or deficient
5 ALERT type 3 Indicator that the structure quality may be low
11 ALERT type 4 Improvement, methodology, query or suggestion
1 ALERT type 5 Informative message, check

It is advisable to attempt to resolve as many as possible of the alerts in all categories. Often the minor alerts point to easily fixed oversights, errors and omissions in your CIF or refinement strategy, so attention to these fine details can be worthwhile. In order to resolve some of the more serious problems it may be necessary to carry out additional measurements or structure refinements. However, the purpose of your study may justify the reported deviations and the more serious of these should normally be commented upon in the discussion or experimental section of a paper or in the "special_details" fields of the CIF. checkCIF was carefully designed to identify outliers and unusual parameters, but every test has its limitations and alerts that are not important in a particular case may appear. Conversely, the absence of alerts does not guarantee there are no aspects of the results needing attention. It is up to the individual to critically assess their own results and, if necessary, seek expert advice.

Publication of your CIF in IUCr journals

A basic structural check has been run on your CIF. These basic checks will be run on all CIFs submitted for publication in IUCr journals (*Acta Crystallographica*, *Journal of Applied Crystallography*, *Journal of Synchrotron Radiation*); however, if you intend to submit to *Acta Crystallographica Section C* or *E* or *IUCrData*, you should make sure that full publication checks are run on the final version of your CIF prior to submission.

Publication of your CIF in other journals

Please refer to the *Notes for Authors* of the relevant journal for any special instructions relating to CIF submission.

PLATON version of 19/02/2022; check.def file version of 19/02/2022

Datablock 14be55-p-1_a - ellipsoid plot

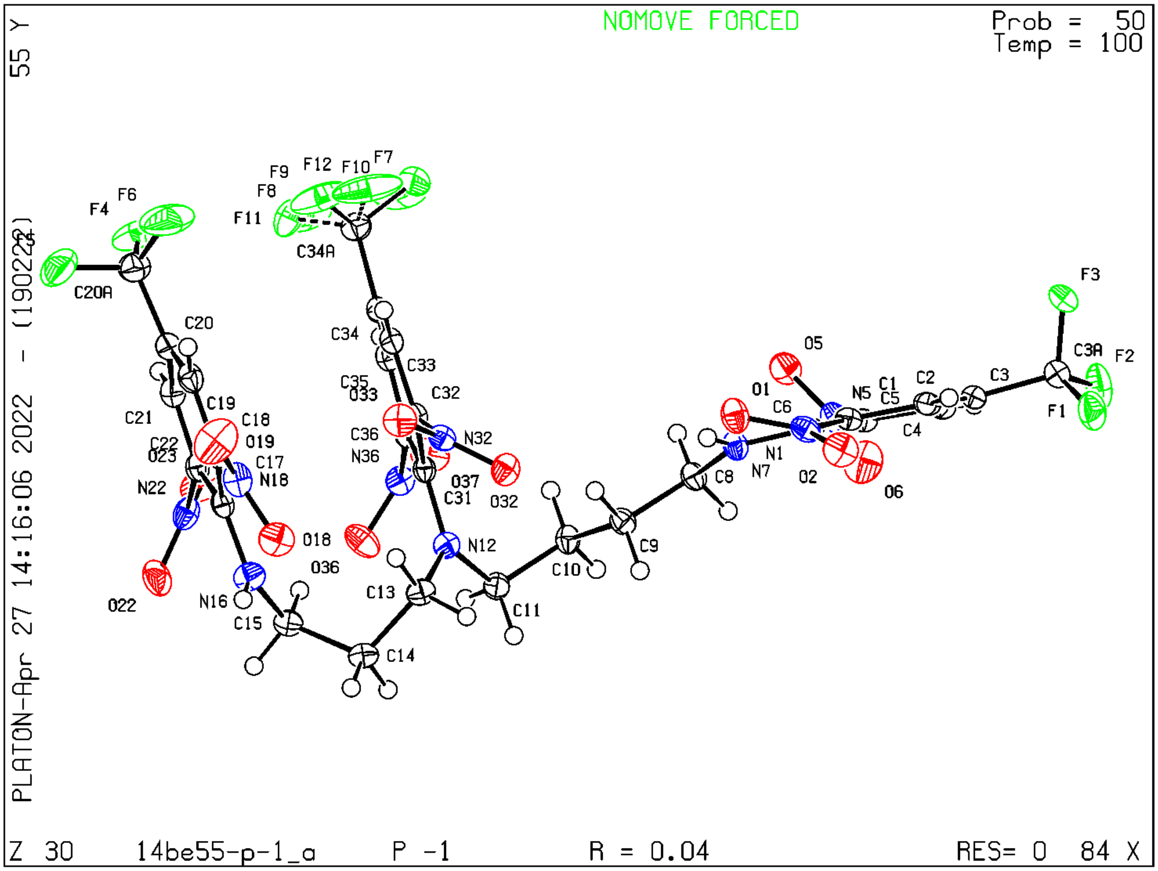

Supplement: Supplementary file 1 [file Spd-CNBF-checkcif.pdf › materials-2144164-supplementary/materials-2144164-supplementary/Supplementary + cif/Spd-CNBF-checkcif.pdf]
